# Supplementary material for: Zinc inhibits lethal inflammatory shock by preventing microbe‐induced interferon signature in intestinal epithelium
Source: EMBO Mol Med. 2020 Sep 11;12(10):e11917. doi: 10.15252/emmm.201911917 (PMC7539219; doi:10.15252/emmm.201911917)
Supplement: Supplementary file 1 — Appendix [file EMMM-12-e11917-s001.pdf]

Appendix

Table of content

Appendix Figures

Appendix Figure S1
Appendix Figure S2
Appendix Figure S3
Appendix Figure S4
Appendix Figure S5
Appendix Figure S6
Appendix Figure S7
Appendix Figure S8
Appendix Figure S9
Appendix Figure S10A
Appendix Figure S10B

Appendix Tables

Appendix Table S1
Appendix Table S2
Appendix Table S3
Appendix Table S4
Appendix Table S5
Appendix Table S6
Appendix Table S7
Appendix Table S8
Appendix Table S9
Appendix Table S10
Appendix Table S11
Appendix Table S12
Appendix Table S13
Appendix Table S14
Appendix Table S15
Appendix Table S16

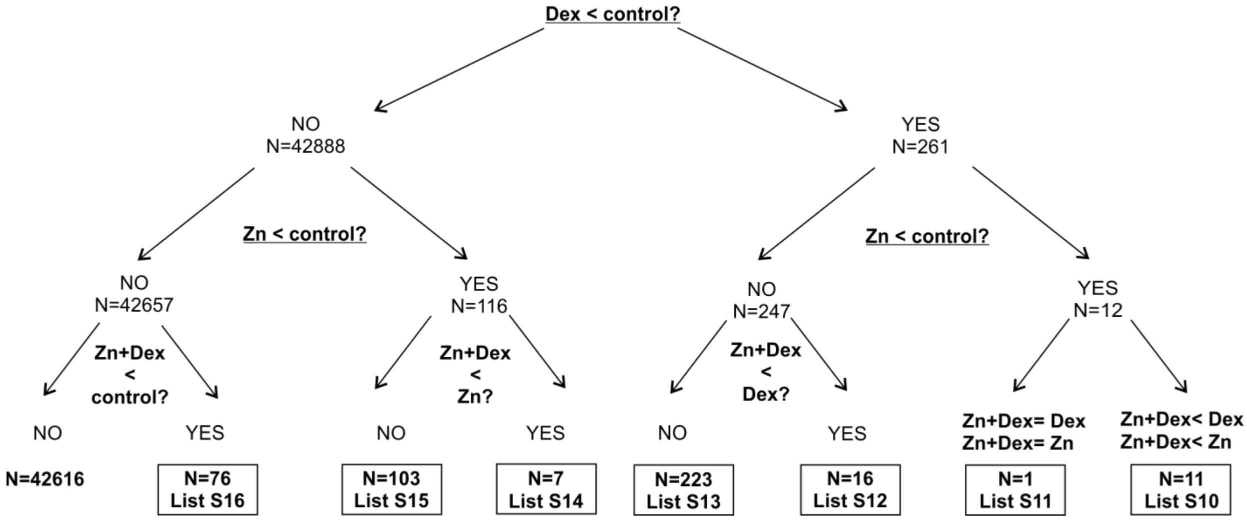

Appendix figure S1: Scheme of numbers of genes down-regulated in $ZnSO_4$, Dex or $ZnSO_4$+Dex treated mice (LFC<-0.8, p<0.05). Significant expression of genes in the RNA sequencing was assessed with a Wald test with negative binomial distribution in DESEQ2.

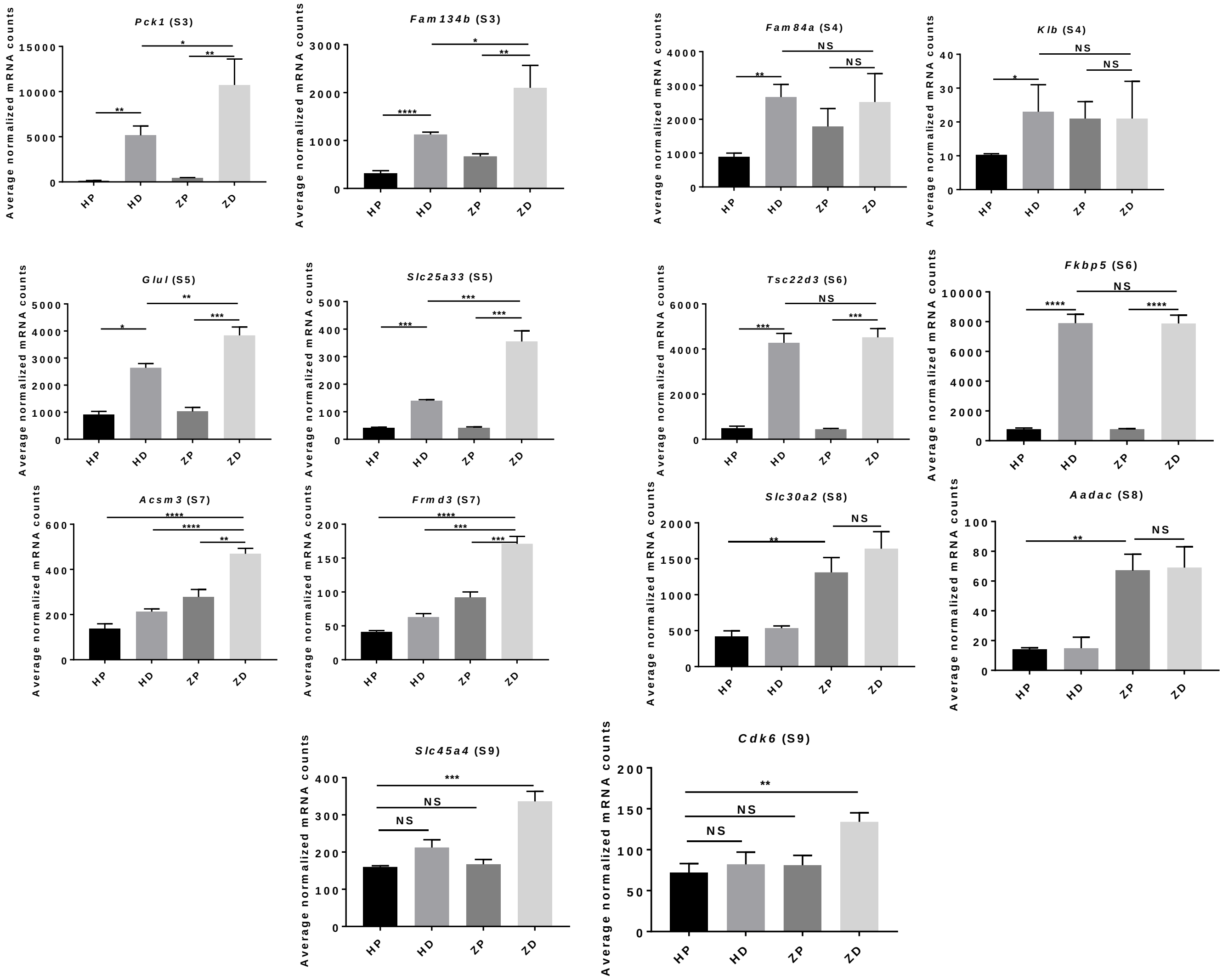

<u>Appendix figure S2:</u> Examples of genes belonging to the arbitrary-deficed groups of Fig. 2C. We provide two example genes for each category (S3 to S9). Statistical analysis were done using One-way ANOVA with multiple comparison. Remark the gene Slc30a2 in groups S8.

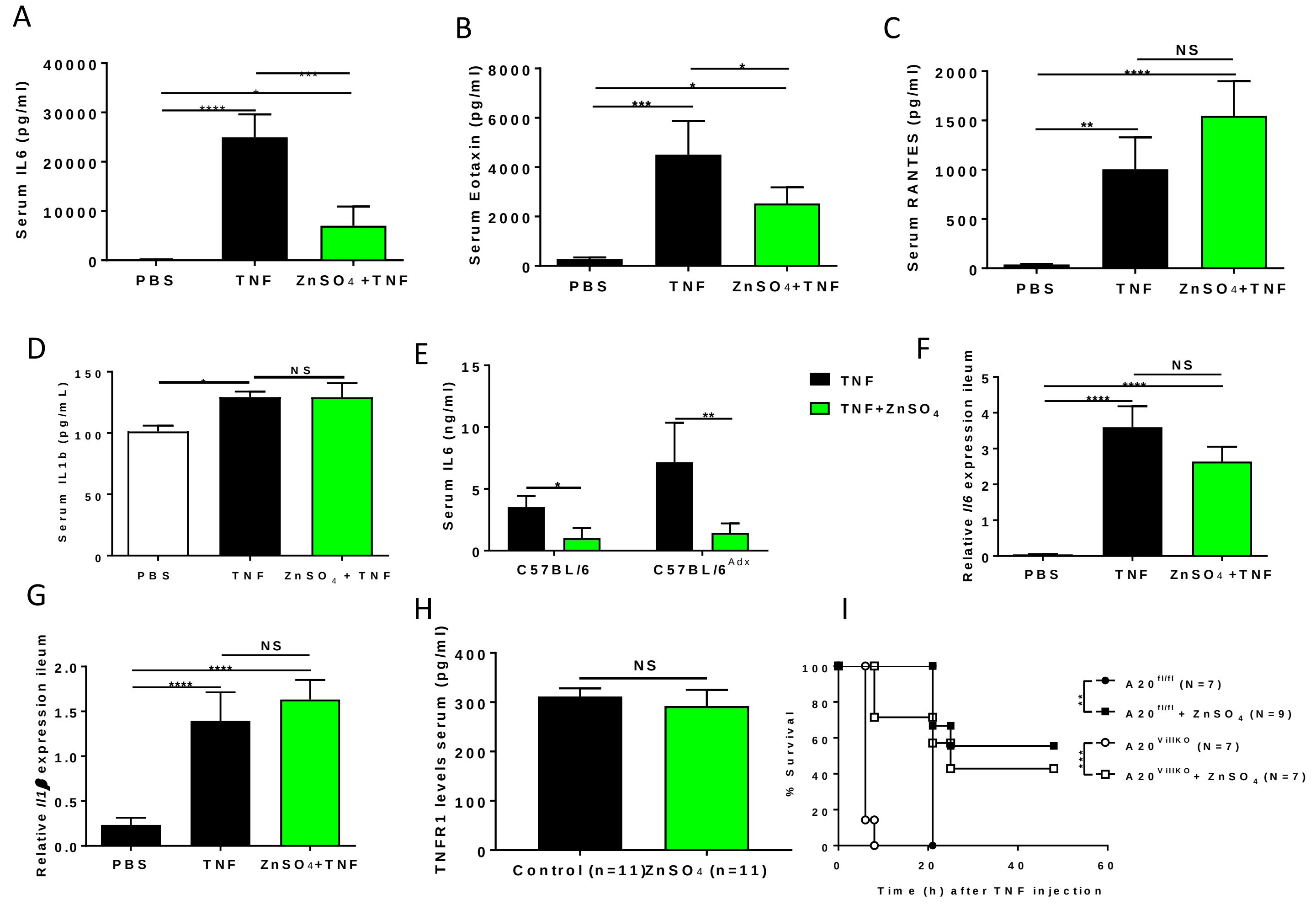

Appendix figure S3: Prophylactic zinc treatment does not induce a general decrease in TNF-induced inflammatory cytokines and chemokines. (A-C) C57BL/6J mice received control or 25 mM ZnSO4 drinking water for 7 days and were injected i.p. with 50 µg TNF, solved in 200 µL sterile PBS, per 20 g bodyweight or with PBS only. 6 h after TNF serum was taken by retro-orbital bleeding and ileum was isolated. Serum IL6 (A), Eotaxin (B), RANTES (C), IL1b (D) levels were determined with bioplex. (E) IL6 levels in the serum of C57BL/6J naïve and adrenalectomized (Adx) mice (determined with a 7TD1 bioassay) that were treated for 7 days with 25 mM ZnSO4 in the drinking water and challenged, i.p., with 30 µg (naïve) or 2 µg (Adx) TNF, solved in sterile PBS, per 20 g bodyweight (N= min. 3 per group). (F-G) Gene-expression of Il6 and Il1β (i) from the experiment of (A-C): mRNAs were determined by RT-qPCR in the ileum. Rpl and Gapdh were used as house-keeping genes. Results represent one experiment (N=min. 4 per group). Data are shown as mean +/- SD and were analyzed with a one-way ANOVA followed by a Tukey multiple comparisons test. (H) 1 week of 25 mM ZnSO4 in the drinking water had no effect on soluble TNFR1 serum levels (I) We found that the absence of the anti-inflammatory protein A20 in the IECs had no diminishing effect on the zinc protection against TNF (Fig. S3H). It must be noted that Tnfaip3 gene expression was found by RNASEQ to be unchanged by zinc in IECs but was induced 7.4-fold in liver (Supplemental Table S1 and S2). *p: ≤ 0.05, **p: ≤ 0.01, ***p: ≤ 0.001, [illegible]

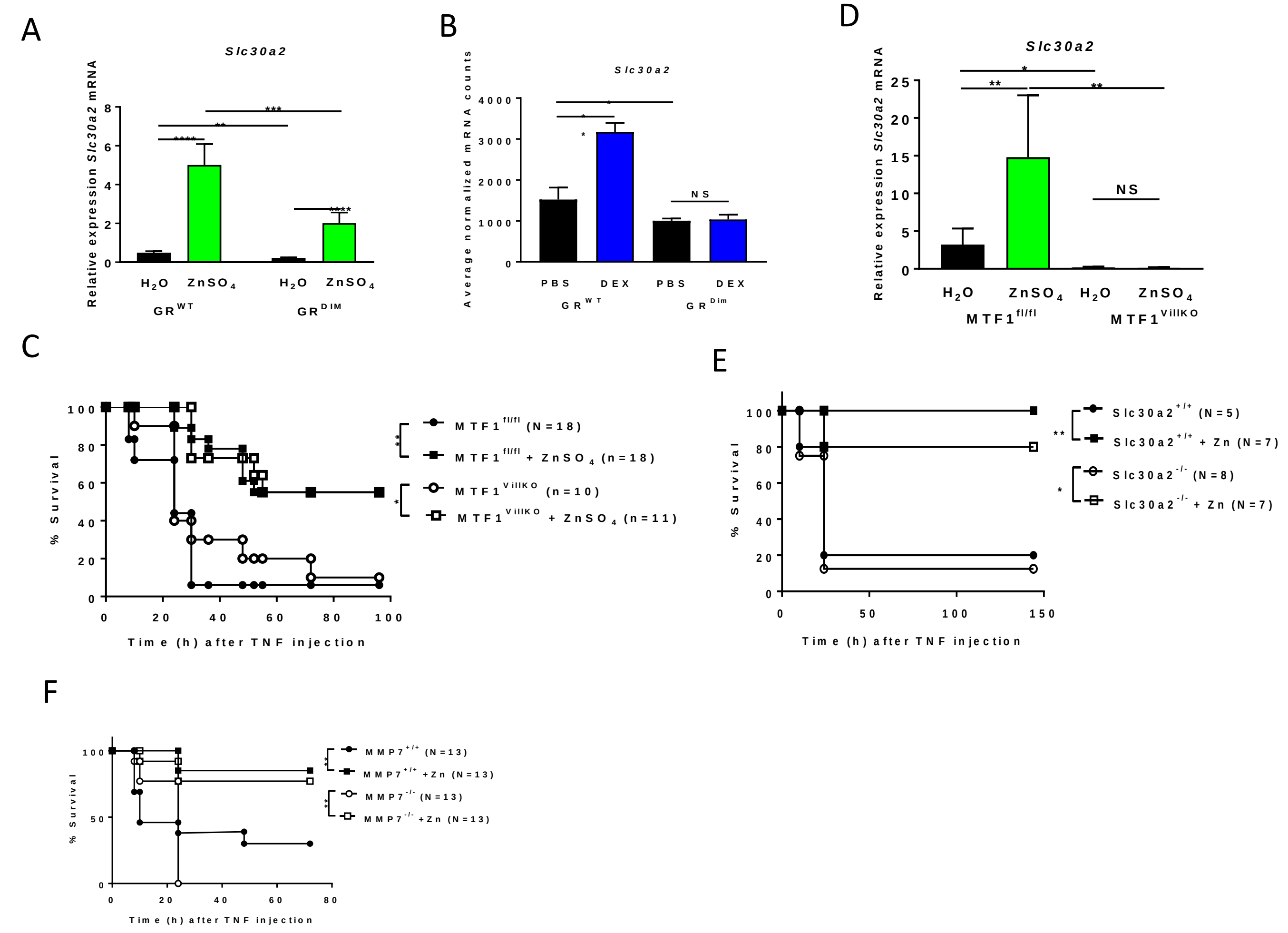

<u>Appendix figure S4</u>: Based on Guo et al., PNAS, 107, 2818, 2010, showing that GR and zinc co-induce Slc30a2, we studied expression of this gene and found that *Slc30a2* was induced by zinc in ileum samples of both GR^{WT} and GR^{Dim} mice, but its expression was significantly less in the GR^{Dim} mice compared to the GR^{WT} mice at both the basal level and after zinc administration (log fold change 1.9 in GR^{WT} and 1.8 in GR^{Dim}). These results were obtained by RT-qPCR (A). Bulk RNASEQ of ileum samples of GR^{WT} and GR^{Dim} mice after i.p. injection of 10 mg/kg Dex confirms that this gene is a true GR dimer dependent GRE gene (B). Since the impact of zinc on *Slc30a2* was shown to depend on MTF1 (C), the major transcription factor responding to zinc, we studied the role of MTF1 in zinc-induced protection against TNF and generated $Slc30a2^{-/-}$ mice. We observed that zinc conferred significant protection against TNF in control $MTF1^{fl/fl}$ mice as well as in $MTF1^{VillKO}$ mice (D), despite strong reduction in MTF1 expression in the latter mice (C). Furthermore, $Slc30a2^{-/-}$ mice, generated in a C57BL/6J background by CRISPR/Cas technology were found to be protected against TNF by ZnSO4 (E). Finally, $MMP7^{-/-}$ mice were equally protected by zinc against TNF, thereby ruling out a role of the contribution of the axis of GR/MTF1-ZnT2-MMP7 in IECs.

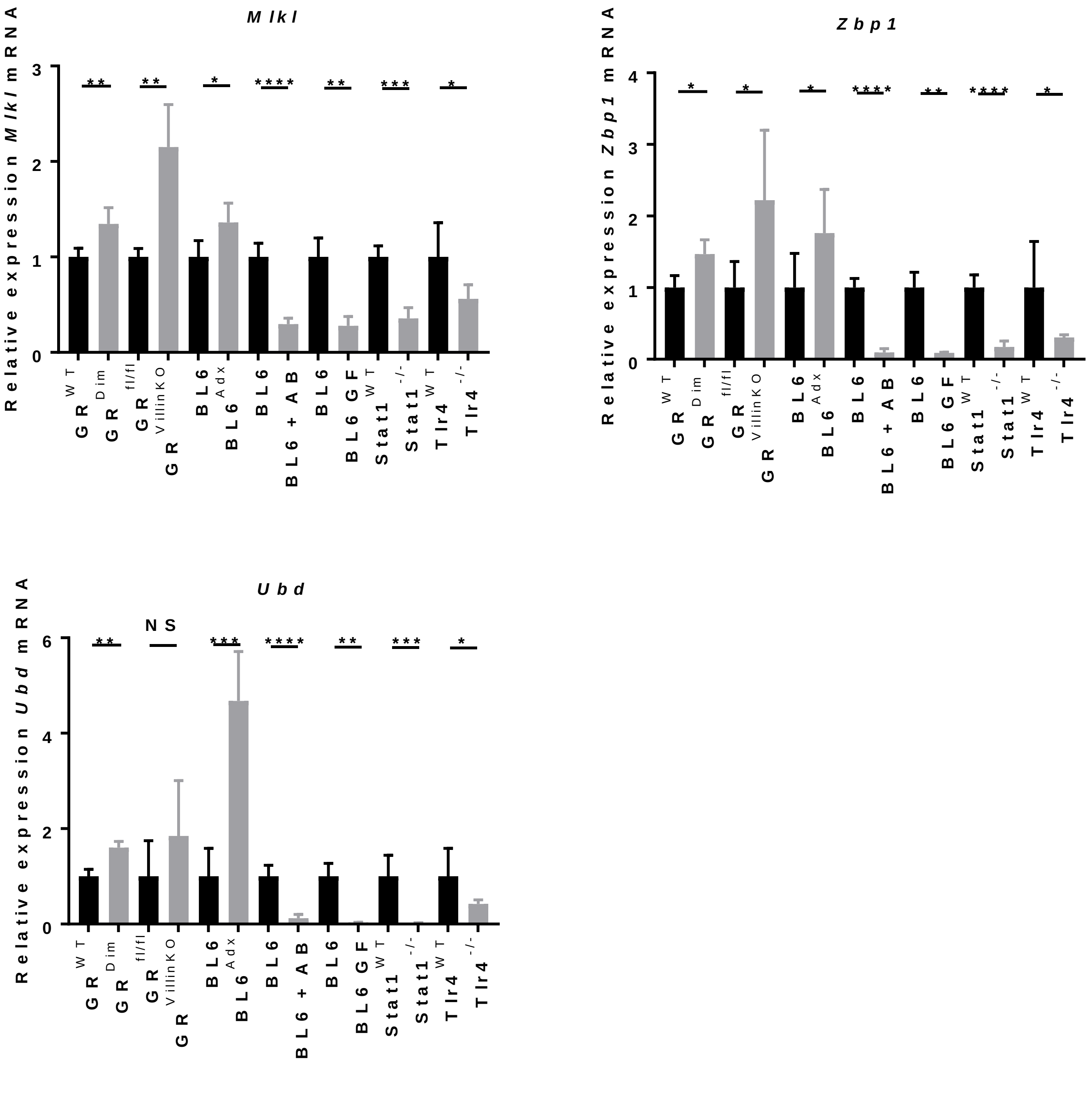

Appendix figure S5: Expression levels of three ISRE/IRF genes (*Mlkl*, *Zbp1* and *Ubd*), the two former of which are essential in the sensitivity of cells for TNF-induced necroptosis, on the mRNA level, determined by RT-qPCR. The relative expression levels of the control group in each couple of mouse groups (controls and mutant or control and treated) was set as 1 to allow to present these expression levels in one overview. It is clear that the mouse groups, which can no longer be protected by zinc (against TNF) have either significantly increased ISRE/IRF gene expressions, or extremely low ones. Data are shown as mean +/- SD. p-value was analyzed by means of a Student's t-test (unpaired, two-tailed).

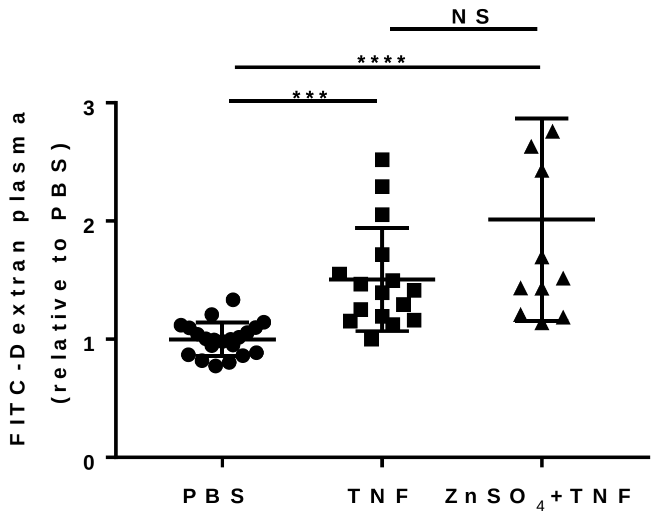

Appendix figure S6: Intestinal permeability assessed by FITC-dextran, 8h after injection of a lethal dose of TNF (35 ug/mouse) in mice pretreated by normal drinking water or $ZnSO_4$ - containing drinking water. One-way ANOVA test.

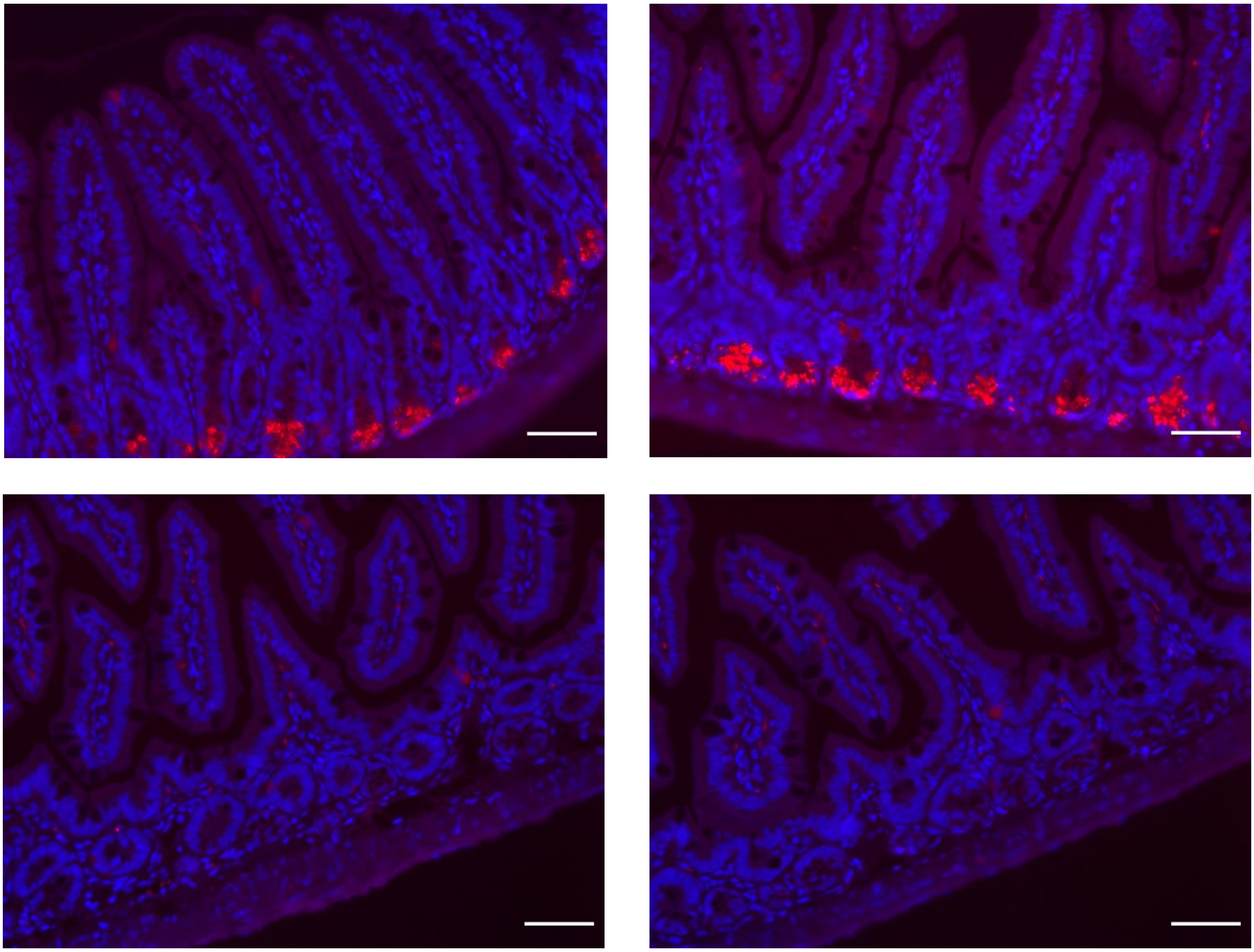

<u>Appendix figure S7: MMP-7</u> Immunohistochemistry on ileum biopsies of two MMP7 +/+ mice (top) and two MMP7-/- mice (bottom). Scale bars: 50 µm.

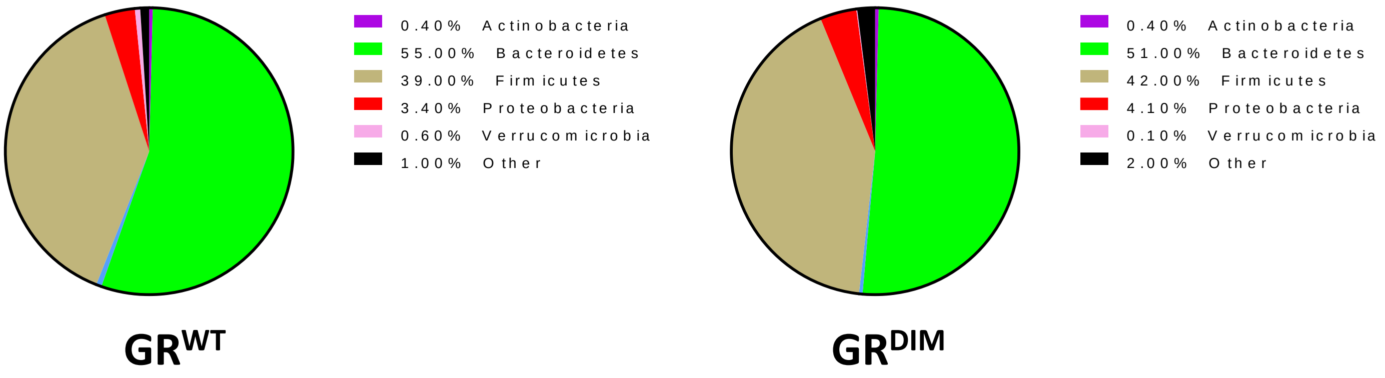

Appendix figure S8: Comparison of the fecal microbiota composition of GR^{WT} (N=40) and GR^{Dim} mice (N=40) by 16S rRNA gene sequencing. Taxonomy on the identified 16S rRNA OTUs was assigned by Silva and analysis was performed with Qiime1. Relative bacterial abundances at the phylum level are displayed. It is clear that the differences between GR^{WT} and GR^{Dim} mice are only very minor.

A

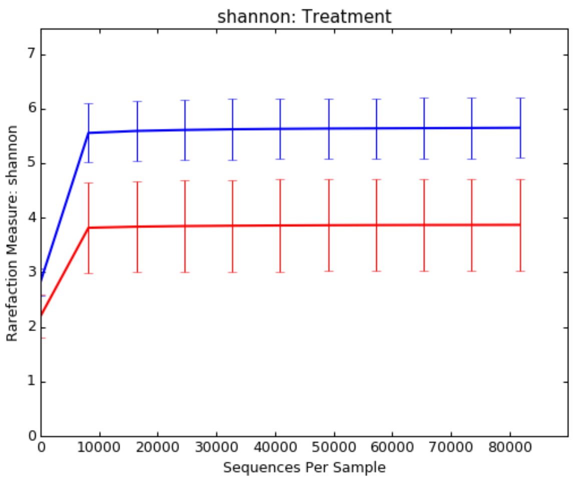

B

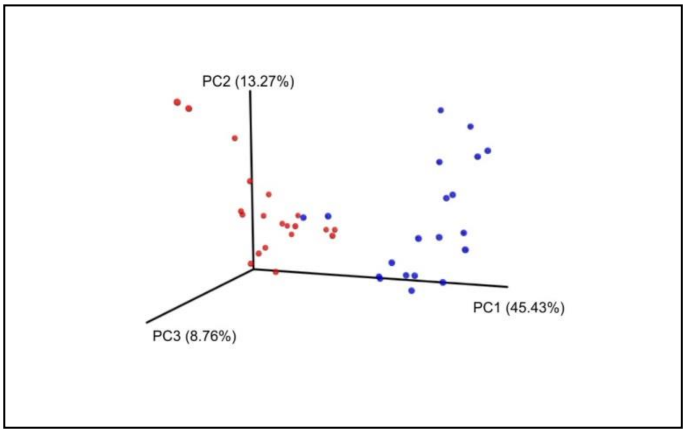

Appendix figure S9: Alpha and beta diversity of the fecal microbiota of C57BL/6J mice that received control (N=20) or 25 mM ZnSO4 (N=20) drinking water for 7 days. 16S rRNA sequencing was performed on feces. Taxonomy on the identified 16S rRNA OTUs was assigned by Silva and analysis was performed with Qiime1. (A) α-diversity determined by use of Shannon Wiender Rarefaction curves (control= blue, zinc=red). (B) Principal Components Plot (PCA) of individual samples analyzed by Bray-Curtis dissimilarity (control=blue, zinc=red).

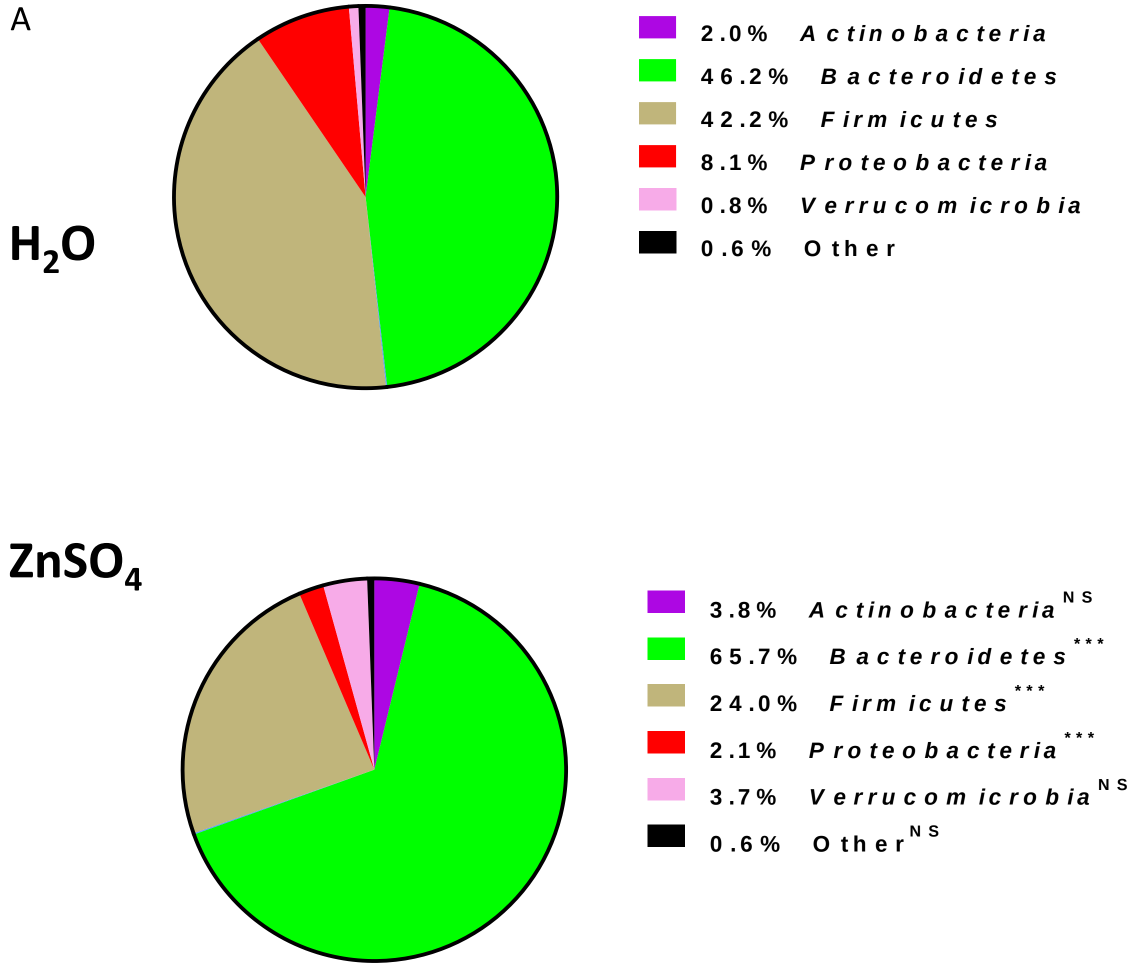

Appendix figure S10A: Relative bacterial abundances at the phylum level in the feces of C57BL/6J mice treated with water (N=20) or 25 mM $ZnSO_4$ (N=20) drinking water for 7 days. 16S rRNA sequencing was performed and taxonomy on the identified 16S rRNA OTUs was assigned by Silva and analysis was performed with Qiime1.

B

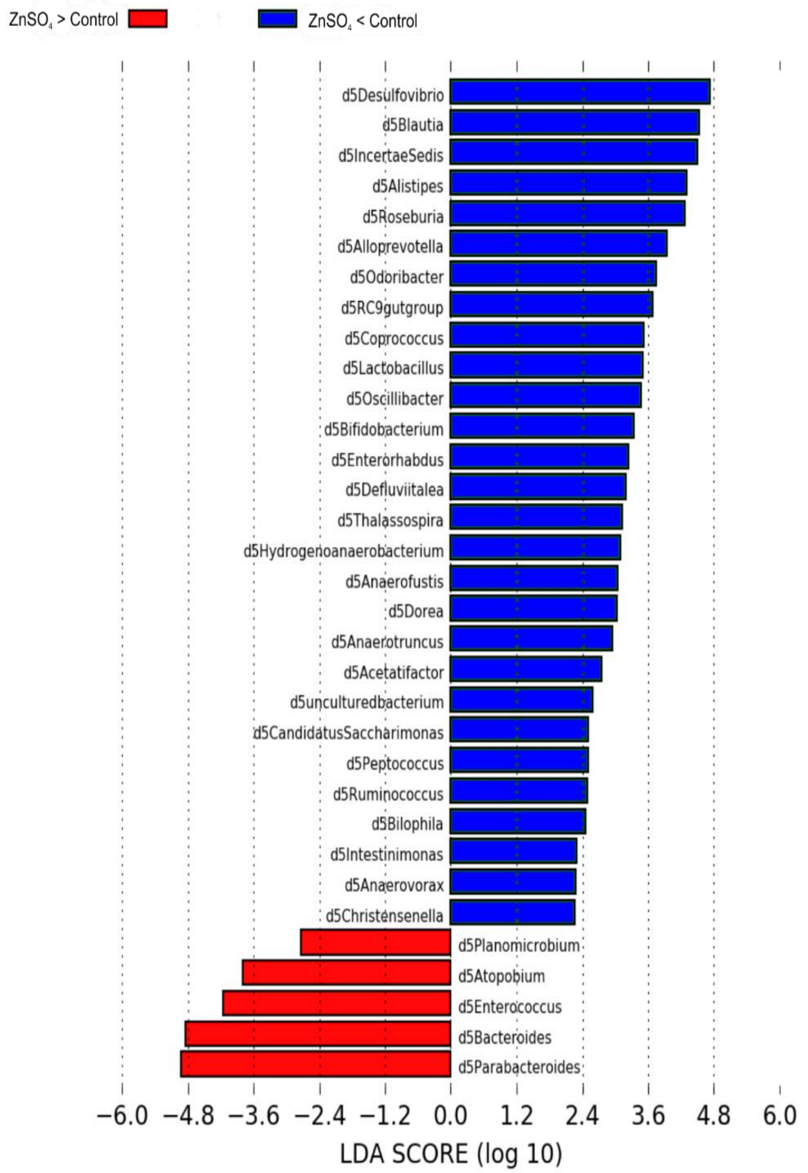

<u>Appendix figure S10</u>B: Significant different taxonomic features between zinc-treated feces (red) and control feces (blue) by use of linear discriminant analysis (LDA) on the samples and data of Fig S10A. Significances of the differences are calculated by chi^2 with multiple testing correction, and are indicated in the lower panel.

Appendix Table S1

List of genes induced and reduced by 1 week 25 mM $ZnSO_4$ in the drinking water in Ileum by RNASEQ.

LFC between $ZnSO_4$ and water.

LFC > 0.8 or < -0.8 and $p<0.05$ are cut-offs.

Ensgene	Symbol	HP	ZP	LFC	pval	Links
ENSMUSG00000031762	Mt2	220.29±91.05	9444.61±2696.81	5.422	2.435e-43	pubmed
ENSMUSG00000031765	Mt1	457.96±106.14	12873.4±3467.96	4.813	6.208e-72	pubmed
ENSMUSG00000053219	Raet1e	13.8±3.49	92.78±25.56	2.748	8.344e-13	pubmed
ENSMUSG00000056035	Cyp3a11	316.42±51.16	1734.75±1004.36	2.455	1.244e-03	pubmed
ENSMUSG00000040134	Rdh7	36.6±7.64	177.25±37.8	2.272	1.500e-27	pubmed
ENSMUSG00000027761	Aadac	14.06±1.14	66.72±11.42	2.249	6.180e-18	pubmed
ENSMUSG00000025196	Cpn1	17.95±5.32	77.77±19.28	2.111	1.902e-16	pubmed
ENSMUSG00000054417	Cyp3a44	55.61±7.08	235.19±163.48	2.081	2.995e-02	pubmed
ENSMUSG00000003555	Cyp17a1	1.38±1.47	5.9±1.44	2.073	4.003e-02	pubmed
ENSMUSG00000040283	Btnl9	2.55±1.13	10.25±2.62	1.988	2.488e-03	pubmed
ENSMUSG00000057074	Ces1g	29.54±5.94	109.29±12.68	1.888	2.087e-14	pubmed
ENSMUSG00000025197	Cyp2c44	2.58±1.78	9.37±4.56	1.854	7.268e-03	pubmed
ENSMUSG00000027513	Pck1	122.88±36.04	441.46±40.49	1.845	1.018e-04	pubmed
ENSMUSG00000027876	Reg4	585.74±149.44	2075.33±651.98	1.825	2.064e-08	pubmed
ENSMUSG00000004038	Gstm3	57.25±13	196.5±79.41	1.779	1.794e-03	pubmed
ENSMUSG00000028427	Aqp7	53.27±8.6	180.31±15.67	1.758	1.907e-07	pubmed
ENSMUSG00000028755	Cda	85.36±12.83	280.88±50.74	1.719	4.950e-15	pubmed
ENSMUSG00000028836	Slc30a2	416.98±79.93	1311.13±207.12	1.653	7.846e-15	pubmed
ENSMUSG00000062410	Hsd3b3	142.47±19.6	446.08±59.79	1.646	2.807e-20	pubmed
ENSMUSG00000041012	Cmtm8	2.56±0.84	7.94±0.49	1.624	2.162e-02	pubmed
ENSMUSG00000074639	BC089597	4.66±1.09	14.31±1.69	1.609	7.050e-04	pubmed
ENSMUSG00000019232	Etnppl	13.52±6.05	40.44±19.61	1.575	3.026e-04	pubmed
ENSMUSG00000024818	Slc25a45	68.25±20.57	197.47±32.41	1.533	3.878e-10	pubmed
ENSMUSG00000040147	Maob	170.22±34	491.25±65.31	1.527	3.246e-28	pubmed
ENSMUSG00000029304	Spp1	4.01±2.23	11.38±4.63	1.510	1.420e-02	pubmed
ENSMUSG00000071604	Fam189a2	82.72±7.16	235.11±37.8	1.506	1.219e-15	pubmed
ENSMUSG00000067231	Cyp2c65	338.06±34.75	927.04±15.74	1.455	7.081e-05	pubmed
ENSMUSG00000048440	Cyp4f16	547.4±40.78	1487.65±76.26	1.442	8.296e-12	pubmed
ENSMUSG00000022445	Cyp2d26	363.68±123.34	967.9±159.74	1.412	4.109e-07	pubmed
ENSMUSG00000071551	Akr1c19	246.13±18.99	646.48±119.64	1.393	6.611e-12	pubmed
ENSMUSG00000021367	Edn1	14.26±2.04	36.81±6.82	1.366	1.025e-03	pubmed
ENSMUSG00000073940	Hbb-bt	16.13±1.43	41.02±23.08	1.349	6.992e-03	pubmed
ENSMUSG00000063730	Hsd3b2	36.03±2.36	90.57±13.92	1.330	4.875e-07	pubmed
ENSMUSG00000026489	Adck3	175.81±13.56	435.94±46.01	1.310	7.895e-07	pubmed
ENSMUSG00000030905	Crym	34.91±5.22	86.39±11.37	1.307	2.318e-05	pubmed
ENSMUSG00000031845	Bco1	68.83±2.46	169.88±46.36	1.303	7.641e-06	pubmed
ENSMUSG00000021751	Acox2	70.18±11.28	173.06±15.68	1.301	3.944e-17	pubmed
ENSMUSG00000030110	Ret	125.49±5.79	307.55±71.23	1.293	4.050e-08	pubmed
ENSMUSG00000069919	Hba-a1	27.34±8.95	66.41±28.87	1.281	1.288e-02	pubmed
ENSMUSG00000040584	Abcb1a	3358.22±520.93	8097.03±1729.38	1.270	2.236e-04	pubmed
ENSMUSG00000027870	Hao2	38.5±10.58	92.89±10.22	1.270	6.535e-03	pubmed
ENSMUSG00000052305	Hbb-bs	95.02±16.95	227.37±109.13	1.259	3.136e-03	pubmed
ENSMUSG00000032348	Gsta4	141.21±33.14	333.84±32.32	1.240	4.392e-08	pubmed
ENSMUSG00000031613	Hpgd	1147.95±118.13	2710.95±518.26	1.240	5.535e-16	pubmed
ENSMUSG00000025474	Tubgcp2	123.63±14.56	290.9±76.79	1.234	4.875e-07	pubmed
ENSMUSG00000026278	Bok	98.54±12.68	229.46±17.24	1.219	6.537e-09	pubmed
ENSMUSG00000058135	Gstm1	222.18±7.05	512.24±59.36	1.205	2.031e-14	pubmed
ENSMUSG00000026433	Rab29	75.35±1.07	172.39±41.49	1.193	2.543e-08	pubmed
ENSMUSG00000071178	Serpina1b	152.29±13.27	344.01±48.7	1.175	7.934e-14	pubmed
ENSMUSG00000049122	Frmd3	41.15±2.31	91.7±7.84	1.154	1.901e-07	pubmed
ENSMUSG00000045019	Acer1	80.5±11.5	179.18±43.09	1.154	1.782e-05	pubmed
ENSMUSG00000028003	Lrat	134.3±17.44	298.58±38.28	1.152	1.653e-08	pubmed
ENSMUSG00000020889	Nr1d1	34.93±4.09	77.55±11.53	1.150	3.146e-05	pubmed
ENSMUSG00000036814	Slc6a20a	830.83±46.33	1838.02±41.28	1.145	4.834e-09	pubmed
ENSMUSG00000035112	Wnk4	34.14±2.04	73.93±1.47	1.114	6.525e-06	pubmed
ENSMUSG00000030155	Clec2e	372.27±76.21	805.73±154.05	1.114	9.239e-04	pubmed
ENSMUSG00000005125	Ndrg1	614.01±98.02	1325.68±154.9	1.111	6.074e-13	pubmed

Ensgene	Symbol	HP	ZP	LFC	pval	Links
ENSMUSG00000034687	Fras1	24.16±2.61	51.9±1.79	1.105	1.115e-03	pubmed
ENSMUSG00000000440	Pparg	59.49±4.68	127.59±21.47	1.100	2.474e-03	pubmed
ENSMUSG00000026870	Cutal	127.56±3.45	273.29±20.63	1.099	2.372e-13	pubmed
ENSMUSG00000033715	Akr1c14	351.36±66.91	750.61±47.11	1.095	4.802e-11	pubmed
ENSMUSG00000032500	Dclk3	30.45±2.6	64.95±5.41	1.093	2.977e-04	pubmed
ENSMUSG00000043789	Vwce	6.79±0.3	14.33±2.7	1.074	3.570e-02	pubmed
ENSMUSG00000022270	Fam134b	318.54±52.5	667.67±55.09	1.068	5.661e-07	pubmed
ENSMUSG00000021557	Agtpbp1	97.31±14.46	203.19±63.06	1.062	2.118e-04	pubmed
ENSMUSG00000030621	Me3	58.31±11.91	121.87±7.39	1.062	2.023e-04	pubmed
ENSMUSG00000001025	S100a6	871.41±127.29	1811.84±466.65	1.056	5.609e-04	pubmed
ENSMUSG00000021213	Akr1c13	376.04±48.86	778.61±139.85	1.050	2.245e-09	pubmed
ENSMUSG00000034528	Hsd17b13	66.68±6.68	136.94±33.06	1.037	1.559e-07	pubmed
ENSMUSG00000054630	Ugt2b5	90.85±10.43	186.04±8.21	1.032	1.418e-14	pubmed
ENSMUSG00000053279	Aldh1a1	1638.06±175.89	3322.95±454.49	1.020	2.882e-06	pubmed
ENSMUSG00000025194	Abcc2	437.91±90.26	885.63±369.24	1.016	1.302e-02	pubmed
ENSMUSG00000056162	Cndp1	109.16±21.23	220.28±40.94	1.012	1.422e-03	pubmed
ENSMUSG00000030935	Acsm3	137.68±20.97	277.96±32.81	1.011	1.065e-11	pubmed
ENSMUSG00000020607	Fam84a	893.47±109.87	1788.49±529.08	1.001	1.185e-02	pubmed
ENSMUSG00000039063	Echdc3	8.44±1.61	16.79±0.95	0.993	1.876e-02	pubmed
ENSMUSG00000022537	Tmem44	24.91±6.69	49.65±3.29	0.991	7.449e-03	pubmed
ENSMUSG00000029195	Klb	10.3±0.31	20.49±5.24	0.989	3.405e-02	pubmed
ENSMUSG00000055737	Ghr	254.42±12.37	501.35±84.98	0.978	7.230e-09	pubmed
ENSMUSG00000020019	Ntn4	149.68±5.87	294.49±7.35	0.977	4.114e-19	pubmed
ENSMUSG00000018217	Pmp22	4492.32±1095.31	8792.19±1069.09	0.969	3.333e-03	pubmed
ENSMUSG00000021379	Id4	76.7±15.89	150.15±36.74	0.968	2.086e-02	pubmed
ENSMUSG00000079164	Tlr5	12.85±3.08	25.1±4.49	0.966	3.098e-02	pubmed
ENSMUSG00000030834	Abcc6	37.56±6.29	72.72±3.92	0.950	3.733e-07	pubmed
ENSMUSG00000028780	Sema3c	225.32±16.49	435.16±73.91	0.949	1.172e-06	pubmed
ENSMUSG00000009614	Sardh	35.99±5.36	68.98±3.12	0.937	1.946e-04	pubmed
ENSMUSG00000068874	Selenbp1	270.24±40.52	516.1±108.74	0.933	5.634e-04	pubmed
ENSMUSG00000025176	Hoga1	7.96±1.76	15.17±1.13	0.932	4.698e-02	pubmed
ENSMUSG00000033721	Vav3	52.85±11.69	100.62±24.59	0.931	4.147e-03	pubmed
ENSMUSG00000070704	Ugt2b36	68.19±7.82	130.28±22.3	0.931	1.805e-08	pubmed
ENSMUSG00000030711	Sult1a1	56.45±7.47	107.34±19.68	0.926	3.243e-05	pubmed
ENSMUSG00000032788	Pdxk	403.81±21.77	762.08±66.43	0.916	7.486e-11	pubmed
ENSMUSG00000041143	Tmco4	283.27±6.78	532.8±128.3	0.911	2.390e-05	pubmed
ENSMUSG00000027792	Bche	451.44±28.64	849.14±152.76	0.911	2.785e-06	pubmed
ENSMUSG00000025515	Muc2	8061.56±171.28	15125.58±2949.21	0.908	3.026e-04	pubmed
ENSMUSG00000027495	Fam210b	238.95±13.91	448.58±60.5	0.908	3.012e-09	pubmed
ENSMUSG00000021125	Arg2	419.63±86.2	781.91±152.15	0.898	4.421e-03	pubmed
ENSMUSG00000037664	Cdkn1c	40.27±0.79	74.88±19.1	0.894	3.883e-03	pubmed
ENSMUSG00000029490	Mfsd7a	52.09±12.21	96.56±16.31	0.890	6.217e-03	pubmed
ENSMUSG00000033720	Sfxn5	10.78±3.24	19.94±4.16	0.889	2.780e-02	pubmed
ENSMUSG00000000531	Grasp	20.59±1.48	38.11±3.33	0.888	2.568e-02	pubmed
ENSMUSG00000044986	Tst	166.18±6.43	306.03±50.69	0.880	1.805e-08	pubmed
ENSMUSG00000028088	Fmo5	1088.31±158.87	2002.69±151.6	0.880	6.820e-06	pubmed
ENSMUSG00000034353	Ramp1	116.37±21.61	213.35±26.1	0.872	5.850e-06	pubmed
ENSMUSG00000034993	Vat1	858.07±68.9	1568.99±139.87	0.870	3.399e-10	pubmed
ENSMUSG00000026659	Dusp12	79.77±15.44	145.81±3.68	0.868	1.434e-06	pubmed
ENSMUSG00000036334	Igsf10	84.08±8.98	152.89±38.33	0.863	3.014e-03	pubmed
ENSMUSG00000002565	Scin	2177.19±488.72	3954.4±304.56	0.861	3.132e-05	pubmed
ENSMUSG00000003352	Cacnb3	261.48±13.5	474.51±62.62	0.859	2.535e-06	pubmed
ENSMUSG00000028967	Errfi1	236.89±46.95	429.34±35.68	0.858	1.133e-02	pubmed
ENSMUSG00000054619	Mettl7a1	218.62±11.62	396.57±30.17	0.858	6.574e-10	pubmed
ENSMUSG00000021136	Smoc1	45.03±10.13	81.4±12.75	0.854	2.622e-04	pubmed
ENSMUSG00000028124	Gclm	785.47±45.54	1418.08±183.96	0.852	2.686e-08	pubmed
ENSMUSG00000044359	P2ry4	148.79±17.65	268.45±40.74	0.851	4.221e-05	pubmed

Ensgene	Symbol	HP	ZP	LFC	pval	Links
ENSMUSG00000036473	Tbc1d24	76.64±13.04	138.17±9.6	0.849	2.137e-04	pubmed
ENSMUSG00000051098	Mblac2	28.4±8.02	51.22±4.76	0.847	1.023e-02	pubmed
ENSMUSG00000039114	Nrn1	25.09±2.69	45.11±12.15	0.845	5.865e-03	pubmed
ENSMUSG00000027187	Cat	1228.72±29.64	2205.47±193.78	0.844	2.218e-15	pubmed
ENSMUSG00000031214	Ophn1	58.61±7.79	105.18±11.25	0.842	9.756e-04	pubmed
ENSMUSG00000023092	Fhl1	695.29±110.99	1243.24±147.13	0.838	1.734e-06	pubmed
ENSMUSG00000068762	Gstm6	15.01±5.01	26.75±1.01	0.836	1.649e-02	pubmed
ENSMUSG00000069727	Gm5595	27.64±3.1	49.32±6.92	0.834	1.175e-02	pubmed
ENSMUSG00000051483	Cbr1	361.87±31.82	644.49±76.83	0.832	6.648e-09	pubmed
ENSMUSG00000042359	Osbpl6	187.63±20.96	333.99±25.81	0.831	1.674e-05	pubmed
ENSMUSG00000013523	Bcas1	424.61±15.61	753.76±90.59	0.828	5.704e-06	pubmed
ENSMUSG00000022822	Abcc5	103.49±12.84	183.73±25	0.828	2.169e-05	pubmed
ENSMUSG00000044252	Osbpl1a	91.03±2.07	161.2±4.01	0.824	6.290e-09	pubmed
ENSMUSG00000037949	Ano10	471.28±41.85	833.07±72.9	0.822	6.665e-07	pubmed
ENSMUSG00000042500	Ago4	56.34±8.73	99.01±2.64	0.815	6.538e-04	pubmed
ENSMUSG00000022098	Bmp1	155.72±9.08	273.12±20.41	0.811	1.069e-11	pubmed
ENSMUSG00000018102	Hist1h2bc	546.93±62.53	959.36±84.66	0.810	3.159e-06	pubmed
ENSMUSG00000031790	Mmp15	306.78±10.88	537.84±22.41	0.810	4.706e-10	pubmed
ENSMUSG00000032377	Plscr4	53.44±8.17	93.71±5.69	0.809	1.599e-04	pubmed
ENSMUSG00000020183	Cpm	353.93±91.64	619.09±114.73	0.807	1.520e-02	pubmed
ENSMUSG00000010307	Tmem86a	248.56±19.44	433.82±37.78	0.803	3.367e-03	pubmed
ENSMUSG00000044461	Shisa2	122.27±18.62	213.25±10.43	0.802	1.158e-03	pubmed
ENSMUSG00000022938	Fam3b	869.14±69.11	497.04±33.16	-0.806	2.719e-03	pubmed
ENSMUSG00000054200	Ffar4	103.42±10.77	59.15±5.66	-0.806	1.506e-02	pubmed
ENSMUSG00000032093	Cd3e	100.89±23.04	57.33±11.42	-0.814	3.155e-02	pubmed
ENSMUSG00000073555	Gm4951	35.26±8.11	19.99±5.29	-0.818	1.399e-02	pubmed
ENSMUSG00000021509	Slc25a48	1103.71±259.5	621.14±110.29	-0.829	3.521e-02	pubmed
ENSMUSG00000016024	Lbp	95.07±17.21	53.15±9.24	-0.836	6.161e-04	pubmed
ENSMUSG00000040253	Gbp7	318.12±48.74	177.69±22.96	-0.840	4.096e-06	pubmed
ENSMUSG00000026149	Tm4sf20	2207±479.4	1227.02±123.77	-0.847	1.946e-04	pubmed
ENSMUSG00000031584	Gsr	2682.05±559.99	1479.69±169.67	-0.858	2.018e-08	pubmed
ENSMUSG00000002992	Apoc2	228.87±3.92	126.18±38.13	-0.861	2.149e-04	pubmed
ENSMUSG00000005947	Itgae	197.23±31.43	108.39±4.68	-0.863	1.272e-03	pubmed
ENSMUSG00000002033	Cd3g	142.41±33.5	78.1±18.5	-0.865	1.717e-02	pubmed
ENSMUSG00000078921	Tgtp2	46.23±6.02	25.29±4.46	-0.866	1.059e-02	pubmed
ENSMUSG00000024353	Mzb1	341.34±78.75	186.67±60.38	-0.870	2.028e-02	pubmed
ENSMUSG00000079507	H2-Q2	427.12±36.68	233.56±68.39	-0.871	1.740e-02	pubmed
ENSMUSG00000049608	Gpr55	191.35±7.38	104.57±18.2	-0.871	1.646e-03	pubmed
ENSMUSG00000034459	Ifit1	446.31±98.12	242.63±56.44	-0.880	3.861e-03	pubmed
ENSMUSG00000026117	Zap70	63.47±19.94	34.28±8.9	-0.885	1.896e-02	pubmed
ENSMUSG00000055415	Atp10b	2042.16±242.02	1105.93±69.78	-0.885	2.928e-11	pubmed
ENSMUSG00000056737	Capg	296.32±61.78	159.81±49.64	-0.890	8.232e-03	pubmed
ENSMUSG00000044165	Bcl2l15	1103.66±200.26	595.13±87.18	-0.891	8.819e-05	pubmed
ENSMUSG00000029530	Ccr9	130.39±17.61	70.09±9.69	-0.895	9.997e-03	pubmed
ENSMUSG00000060950	Trmt61a	64.95±5.8	34.94±7.24	-0.896	3.862e-03	pubmed
ENSMUSG00000057346	Apol9a	71.71±20.35	38.44±12	-0.900	1.841e-02	pubmed
ENSMUSG00000019872	Smpdl3a	1719.56±147.45	919.72±69.82	-0.903	4.084e-14	pubmed
ENSMUSG00000028864	Hgf	62.59±16.75	33.31±0.97	-0.909	9.781e-04	pubmed
ENSMUSG00000060586	H2-Eb1	4261.77±970.81	2253.66±491.77	-0.919	5.345e-04	pubmed
ENSMUSG00000038751	Ptk6	681.24±127.01	358.92±25.05	-0.925	1.454e-02	pubmed
ENSMUSG00000021850	1700011H14Rik	310.72±14.06	163.5±14.45	-0.926	5.512e-08	pubmed
ENSMUSG00000023272	Creld2	547.95±101.62	285.64±22.74	-0.940	4.802e-02	pubmed
ENSMUSG00000028270	Gbp2	535.29±130.31	277.39±48.63	-0.948	1.309e-03	pubmed
ENSMUSG00000054169	Ceacam10	320.44±32.78	164.7±34.82	-0.960	3.309e-03	pubmed
ENSMUSG00000031410	Nxf7	93.5±7.03	47.55±2.52	-0.975	4.046e-04	pubmed
ENSMUSG00000044827	Tlr1	155.67±31.73	79.02±23.87	-0.977	6.677e-03	pubmed
ENSMUSG00000069792	Wfdc17	29.98±2.54	15.24±2.39	-0.980	2.213e-03	pubmed

Ensgene	Symbol	HP	ZP	LFC	pval	Links
ENSMUSG00000072620	Slfn2	215.25±77.57	107.18±51.27	-1.004	1.787e-02	pubmed
ENSMUSG00000034438	Gbp8	155.75±11.57	76.55±13.55	-1.023	5.597e-05	pubmed
ENSMUSG00000078920	Ifi47	641.49±125.77	311.53±29.08	-1.042	5.325e-06	pubmed
ENSMUSG00000033213	AA467197	701.92±61.22	340.12±6.91	-1.045	7.070e-05	pubmed
ENSMUSG00000061762	Tac1	170.63±34.71	82.2±6.51	-1.054	4.021e-05	pubmed
ENSMUSG00000073421	H2-Ab1	6155.39±893.8	2963.28±486.3	-1.055	9.762e-06	pubmed
ENSMUSG00000037649	H2-DMa	741.51±116.46	353.77±65.79	-1.067	2.458e-08	pubmed
ENSMUSG00000055413	H2-Q5	54.42±8.98	25.83±10.64	-1.070	1.552e-02	pubmed
ENSMUSG00000030107	Usp18	81.01±26.09	38.56±3.8	-1.070	3.204e-03	pubmed
ENSMUSG00000037321	Tap1	866.34±176.71	411.18±45.48	-1.075	1.977e-06	pubmed
ENSMUSG00000029657	Hsph1	781.37±303.46	370.5±22.75	-1.077	3.766e-02	pubmed
ENSMUSG00000024610	Cd74	15707.85±2737.99	7445.84±1309.47	-1.077	3.181e-06	pubmed
ENSMUSG00000058624	Gda	5659.12±494.73	2674.6±352.3	-1.081	3.573e-07	pubmed
ENSMUSG00000039699	Batf2	270.42±8.12	127.48±23.56	-1.085	1.805e-08	pubmed
ENSMUSG00000070348	Ccnd1	1586.11±197.49	747.62±44.8	-1.085	3.315e-04	pubmed
ENSMUSG00000036216	Leap2	23.13±7.44	10.87±5.92	-1.090	4.471e-02	pubmed
ENSMUSG00000024887	Asah2	1024.61±276.01	479.78±202.39	-1.095	5.264e-03	pubmed
ENSMUSG00000050982	Apol10a	3336.26±168.28	1559.24±232.11	-1.097	2.011e-05	pubmed
ENSMUSG00000028737	Aldh4a1	151.22±26.51	70.45±14.83	-1.104	1.956e-07	pubmed
ENSMUSG00000025163	Cd7	156.05±14	72.36±7.49	-1.109	1.214e-05	pubmed
ENSMUSG00000028028	Alpk1	248.23±21.35	114.34±13.5	-1.118	3.709e-08	pubmed
ENSMUSG00000074345	Tnfaip8l3	171.3±34.89	78.86±2.4	-1.119	9.239e-04	pubmed
ENSMUSG00000024338	Psmb8	1302.56±164.76	599.02±32.78	-1.120	5.237e-12	pubmed
ENSMUSG00000037820	Tgm2	2762.53±206.12	1262.11±39.23	-1.130	3.392e-13	pubmed
ENSMUSG00000041481	Serpina3g	201.59±89.11	91.77±31.07	-1.134	7.550e-03	pubmed
ENSMUSG00000035042	Ccl5	289.89±69.64	131.69±19.91	-1.139	1.647e-03	pubmed
ENSMUSG00000053977	Cd8a	189.32±24.18	85.21±13.08	-1.151	4.878e-05	pubmed
ENSMUSG00000063206	Gm15315	930.72±141.35	414.86±101.64	-1.166	1.372e-03	pubmed
ENSMUSG00000042677	Zc3h12a	1062.44±117.28	471.9±40.47	-1.171	1.663e-03	pubmed
ENSMUSG00000096727	Psmb9	565.73±60.14	250.78±16.17	-1.173	1.556e-09	pubmed
ENSMUSG00000029084	Cd38	2460.28±297.53	1075.49±93.8	-1.194	1.209e-17	pubmed
ENSMUSG00000030786	Itgam	374.71±2.1	163.52±7.21	-1.196	5.350e-12	pubmed
ENSMUSG00000042808	Gpx2	4728.82±871	2047.79±240.54	-1.207	4.687e-12	pubmed
ENSMUSG00000054072	Iigp1	368.29±94.76	158.97±14.73	-1.211	1.572e-08	pubmed
ENSMUSG00000021553	Slc28a3	233.28±32.25	100.24±2.74	-1.218	3.020e-14	pubmed
ENSMUSG00000037095	Lrg1	76.05±34.2	32.46±7.97	-1.225	1.302e-02	pubmed
ENSMUSG00000047228	BC048546	15.4±4.33	6.57±0.32	-1.230	2.980e-02	pubmed
ENSMUSG00000036594	H2-Aa	7573.16±1230.06	3210.28±691.17	-1.238	4.662e-07	pubmed
ENSMUSG00000026880	Stom	2986.99±792.43	1251.77±125.8	-1.255	1.284e-07	pubmed
ENSMUSG00000031551	Ido1	470.09±72.24	195.8±14.69	-1.264	9.407e-09	pubmed
ENSMUSG00000022847	Thpo	34.69±9.8	14.4±4.73	-1.265	2.344e-04	pubmed
ENSMUSG00000068227	Il2rb	98.6±36.31	39.78±15.22	-1.306	1.420e-03	pubmed
ENSMUSG00000016496	Cd274	185.29±63.48	74.82±10.36	-1.307	6.069e-05	pubmed
ENSMUSG00000069874	Irgm2	780.32±235.97	313.31±53.28	-1.316	1.023e-07	pubmed
ENSMUSG00000039217	Il18	231.16±41.08	91.18±29.16	-1.344	4.584e-09	pubmed
ENSMUSG00000037145	2210407C18Rik	643.49±77.62	253.52±45.06	-1.344	3.247e-05	pubmed
ENSMUSG00000079547	H2-DMb1	620.24±35.1	244.3±11.22	-1.344	3.169e-09	pubmed
ENSMUSG00000074151	Nlrc5	236.19±42.88	92.87±24.05	-1.344	5.262e-09	pubmed
ENSMUSG00000026773	Pfkfb3	633.66±114.44	248.84±62.75	-1.348	2.854e-05	pubmed
ENSMUSG00000043592	Unc5cl	443.59±80.62	171.45±6.12	-1.371	1.193e-15	pubmed
ENSMUSG00000013653	1810065E05Rik	707.06±198.62	264.89±83.67	-1.416	2.622e-04	pubmed
ENSMUSG00000090942	F830016B08Rik	14.56±2.57	5.41±1.33	-1.418	5.665e-03	pubmed
ENSMUSG00000029798	Herc6	245.95±34.54	89.95±1.67	-1.450	4.238e-19	pubmed
ENSMUSG00000079362	Gbp6	340.01±67.38	119.13±16.18	-1.513	8.631e-10	pubmed
ENSMUSG00000055312	Them7	6.59±1.85	2.28±0.68	-1.532	4.914e-02	pubmed
ENSMUSG00000000805	Car4	1141.2±516.06	388.31±80.89	-1.555	3.926e-03	pubmed
ENSMUSG00000079180	Mptx2	5424.81±1186.22	1841.32±332.23	-1.559	4.016e-02	pubmed

Ensgene	Symbol	HP	ZP	LFC	pval	Links
ENSMUSG00000000628	Hk2	893.81±91.77	300.02±26.56	-1.575	1.347e-21	pubmed
ENSMUSG00000095794	Igkv6-17	294.79±92.92	98.78±51.04	-1.578	1.832e-02	pubmed
ENSMUSG00000018387	Shroom1	51.34±10.04	16.82±5.78	-1.613	2.331e-06	pubmed
ENSMUSG00000076934	Iglv1	625.49±172.94	201.88±76.44	-1.631	1.059e-02	pubmed
ENSMUSG00000046688	Tifa	1113.79±234.52	354.61±36.19	-1.651	1.732e-13	pubmed
ENSMUSG00000058163	Gm5431	242.57±87.07	76.87±3.43	-1.657	5.964e-07	pubmed
ENSMUSG00000029417	Cxcl9	178.21±67.99	56.44±0.18	-1.658	1.168e-06	pubmed
ENSMUSG00000032033	Barx2	238.85±19.91	71.72±4.34	-1.736	2.721e-20	pubmed
ENSMUSG00000028655	Mfsd2a	59.63±11.68	17.6±4.38	-1.757	1.887e-07	pubmed
ENSMUSG00000028885	Smpdl3b	444.41±68.65	127.88±10.28	-1.797	3.211e-11	pubmed
ENSMUSG00000020826	Nos2	3155.72±477.64	906.43±220.92	-1.800	3.775e-04	pubmed
ENSMUSG00000078853	Igtp	566.62±179.19	161.54±14.69	-1.810	8.134e-12	pubmed
ENSMUSG00000061947	Serpina10	19.26±6.57	5.35±4.23	-1.820	8.706e-06	pubmed
ENSMUSG00000023132	Gzma	868.1±162.68	241.77±58.85	-1.845	3.886e-09	pubmed
ENSMUSG00000047517	Dmbt1	18537.56±7356.43	4775.3±1094.22	-1.957	8.817e-06	pubmed
ENSMUSG00000042102	Dmgdh	9.82±4.36	2.5±0.66	-1.979	2.488e-03	pubmed
ENSMUSG00000082292	Gm12250	133.56±33.84	33.85±3.43	-1.981	2.372e-13	pubmed
ENSMUSG00000066363	Serpina3f	44.62±19.81	10.89±1.21	-2.033	3.542e-06	pubmed
ENSMUSG00000076587	Igkv6-20	102.41±42.65	24.79±8.93	-2.046	1.949e-02	pubmed
ENSMUSG00000076514	Igkv17-121	176.6±102.24	41.52±11.54	-2.088	1.641e-03	pubmed
ENSMUSG00000070427	Il18bp	210.31±41.49	47.59±10.63	-2.140	5.392e-22	pubmed
ENSMUSG00000079339	Ifit1bl1	2556.61±547.59	575.42±50.98	-2.152	2.386e-29	pubmed
ENSMUSG00000041193	Pla2g5	213.02±45.98	47.58±5.69	-2.162	5.181e-19	pubmed
ENSMUSG00000093896	Ighv1-76	265.63±172.96	58.21±39.99	-2.191	3.506e-02	pubmed
ENSMUSG00000015437	Gzmb	381.85±107.82	79.21±17.87	-2.270	1.822e-10	pubmed
ENSMUSG00000076556	Igkv4-57	962.43±483.75	195.7±36.52	-2.298	6.040e-05	pubmed
ENSMUSG00000058952	Cfi	73.18±20.15	14.78±2.83	-2.310	1.387e-17	pubmed
ENSMUSG00000027514	Zbp1	581.74±123.71	117.19±14.21	-2.311	1.392e-21	pubmed
ENSMUSG00000079445	B3gnt7	740.06±189.24	148.44±19.23	-2.318	2.149e-22	pubmed
ENSMUSG00000068452	Duox2	3883.6±310.71	779.02±79.53	-2.318	3.356e-19	pubmed
ENSMUSG00000094694	Ighv1-9	978.4±69.18	194.48±120.15	-2.331	4.373e-02	pubmed
ENSMUSG00000020407	Upp1	3408.47±375	629.96±59.01	-2.436	3.446e-54	pubmed
ENSMUSG00000063388	BC023105	4.93±0.65	0.92±0.65	-2.438	2.951e-02	pubmed
ENSMUSG00000055978	Fut2	145.93±44.14	24.86±5.87	-2.552	1.610e-09	pubmed
ENSMUSG00000024863	Mbl2	13.15±2.08	2.04±0.58	-2.684	2.070e-05	pubmed
ENSMUSG00000036123	Slc9a3	150.72±65.46	23.1±1	-2.705	1.487e-11	pubmed
ENSMUSG00000074892	B3galt5	145.36±82.41	20.12±2.58	-2.852	3.528e-08	pubmed
ENSMUSG00000074768	Bhmt	55±37.85	7.55±3.23	-2.867	1.908e-03	pubmed
ENSMUSG00000063354	Slc39a4	1717.2±69.61	219.28±26.8	-2.969	9.445e-77	pubmed
ENSMUSG00000094689	Ighv1-81	276.54±139.58	33.98±7.18	-3.025	3.712e-04	pubmed
ENSMUSG00000030017	Reg3g	75905.19±24575.39	8753.08±1914.38	-3.116	1.088e-02	pubmed
ENSMUSG00000035186	Ubd	406.04±138.7	44.07±26.18	-3.202	8.782e-07	pubmed
ENSMUSG00000001670	Tat	200.35±53.22	21.07±3.3	-3.248	7.633e-29	pubmed
ENSMUSG00000027225	Duoxa2	1309.53±112.72	137.84±21	-3.248	3.645e-26	pubmed
ENSMUSG00000057465	Saa2	6.82±1.55	0.46±0.65	-3.906	2.608e-02	pubmed
ENSMUSG00000023176	Cpn2	4.71±3.7	0.23±0.32	-4.369	3.593e-02	pubmed
ENSMUSG00000074115	Saa1	2308.41±726.19	60.96±34.19	-5.243	3.357e-09	pubmed

Appendix Table S2

List of genes induced and reduced by 1 week 25 mM $ZnSO_4$ in the drinking water in liver by RNASEQ.

LFC between $ZnSO_4$ and water.

LFC > 0.8 or < -0.8 and $p<0.05$ are cut-offs.

Ensgene	Symbol	HP	ZP	LFC	pval	Links
ENSMUSG00000031762	Mt2	122.72±53.18	4802.4±1332.87	5.287	3.627e-39	pubmed
ENSMUSG00000031765	Mt1	432.27±127.79	6359.42±1485.07	3.879	7.249e-45	pubmed
ENSMUSG00000019850	Tnfaip3	49.44±5.56	366.06±405.11	2.889	1.390e-02	pubmed
ENSMUSG00000013643	Lypd8	5.86±3.43	30.19±18.44	2.351	3.336e-02	pubmed
ENSMUSG00000027995	Tlr2	51.02±4.36	182.07±154.13	1.833	2.478e-02	pubmed
ENSMUSG00000020205	Phlda1	412.02±60.13	1155.91±459.99	1.487	1.068e-04	pubmed
ENSMUSG00000034774	Dsg1c	49.9±17.37	127.65±29	1.360	1.390e-02	pubmed
ENSMUSG00000062209	Erbb4	59.07±7.33	130.55±27.85	1.139	1.390e-02	pubmed
ENSMUSG00000024036	Slc37a1	35.38±4.95	74.97±16.56	1.085	1.544e-03	pubmed
ENSMUSG00000027075	Slc43a1	70.73±20.34	139.03±17.93	0.980	3.121e-02	pubmed
ENSMUSG00000041782	Lad1	40.4±6.85	76.87±12.78	0.942	1.937e-02	pubmed
ENSMUSG00000025402	Nab2	232.32±40.62	435.56±45.29	0.908	1.390e-02	pubmed
ENSMUSG00000040891	Foxa3	585.27±14.43	1079.02±184.05	0.882	1.172e-07	pubmed
ENSMUSG00000050549	5730508B09Rik	39.46±7.27	71.62±4.9	0.874	3.662e-02	pubmed
ENSMUSG00000053886	Sh2d4a	182.69±25.02	330.36±38.24	0.856	2.714e-05	pubmed
ENSMUSG00000020638	Cmpk2	189.07±27.74	108.43±13.18	-0.806	2.217e-02	pubmed
ENSMUSG00000055116	Arntl	315.07±38.12	162.86±29.96	-0.950	1.973e-02	pubmed
ENSMUSG00000058672	Tubb2a	1147.91±348.42	531.95±83.69	-1.110	8.418e-03	pubmed
ENSMUSG00000034413	Neurl1b	30.48±9.99	11.35±1.28	-1.449	2.910e-02	pubmed
ENSMUSG00000063354	Slc39a4	834.31±83.58	292.16±28.13	-1.514	2.016e-17	pubmed
ENSMUSG00000026077	Npas2	54.16±17.93	18.14±10.58	-1.575	2.220e-02	pubmed
ENSMUSG00000026819	Slc25a25	2072.03±511.9	683.74±54.03	-1.600	1.303e-11	pubmed
ENSMUSG00000078650	G6pc	3287.74±812.29	997.36±229.66	-1.721	2.217e-02	pubmed

Appendix Table S3

List of genes derived from the RNASEQ experiment shown in Fig. 2. Definitions of the categories see Fig. 2.

Ensgene	Symbol	HP	HD	ZP	ZD	LFC HD-HP	FDR HD-HP	LFC ZP-HP	FDR ZP-HP	LFC ZD-HP	FDR ZD-HP	Ratio	Links
ENSMUSG00000010307	Tmem86a	248.56±19.44	1606.58±254.54	433.82±37.78	1769.18±361.05	2.692	0.00000	0.803	0.00337	2.831	0.00000	0.00000	pubmed
ENSMUSG00000020183	Cpm	353.93±91.64	828.45±58.67	619.09±114.73	1165.03±131.8	1.227	0.00002	0.807	0.01520	1.719	0.00000	0.00000	pubmed
ENSMUSG00000021125	Arg2	419.63±86.2	1291.32±235.37	781.91±152.15	1687.04±344.72	1.621	0.00000	0.898	0.00442	2.007	0.00000	0.00000	pubmed
ENSMUSG00000021367	Edn1	14.26±2.04	72.52±18.07	36.81±6.82	127±21.56	2.346	0.00000	1.366	0.00102	3.153	0.00000	0.00000	pubmed
ENSMUSG00000022270	Fam134b	318.54±52.5	1126.77±48.72	667.67±55.09	2102.44±466.16	1.823	0.00000	1.068	0.00000	2.722	0.00000	0.00000	pubmed
ENSMUSG00000027513	Pck1	122.88±36.04	5154.03±1034.41	441.46±40.49	10737.46±2863.54	5.391	0.00000	1.845	0.00010	6.450	0.00000	0.00000	pubmed
ENSMUSG00000028967	Errfi1	236.89±46.95	2250.13±108.81	429.34±35.68	2529.15±274.07	3.247	0.00000	0.858	0.01133	3.416	0.00000	0.00000	pubmed
ENSMUSG00000030711	Sult1a1	56.45±7.47	261.12±31.17	107.34±19.68	357.78±17.02	2.210	0.00000	0.926	0.00003	2.665	0.00000	0.00000	pubmed
ENSMUSG00000031613	Hpgd	1147.95±118.13	4976.43±242.6	2710.95±518.26	7334.91±1068.46	2.116	0.00000	1.240	0.00000	2.676	0.00000	0.00000	pubmed
ENSMUSG00000031765	Mt1	457.96±106.14	812.82±89.69	12873.4±3467.96	17699.15±1296.21	0.828	0.01648	4.813	0.00000	5.272	0.00000	0.00000	pubmed
ENSMUSG00000031845	Bco1	68.83±2.46	315.24±32.05	169.88±46.36	768.86±201.86	2.196	0.00000	1.303	0.00001	3.481	0.00000	0.00000	pubmed
ENSMUSG00000037664	Cdkn1c	40.27±0.79	108.04±1.09	74.88±19.1	130.04±15.18	1.424	0.00000	0.894	0.00388	1.690	0.00000	0.00000	pubmed
ENSMUSG00000039114	Nrn1	25.09±2.69	53.46±4.76	45.11±12.15	93.47±11.01	1.093	0.00006	0.845	0.00586	1.901	0.00000	0.00000	pubmed
ENSMUSG00000040134	Rdh7	36.6±7.64	79.73±18.01	177.25±37.8	254.82±39.67	1.121	0.00000	2.272	0.00000	2.795	0.00000	0.00000	pubmed

Appendix Table S4

List of genes derived from the RNASEQ experiment shown in Fig. 2. Definitions of the categories see Fig. 2.

Ensgene	Symbol	HP	HD	ZP	ZD	LFC HD-HP	FDR HD-HP	LFC ZP-HP	FDR ZP-HP	LFC ZD-HP	FDR ZD-HP	Ratio	Links
ENSMUSG00000020607	Fam84a	893.47±109.87	2658.82±371.02	1788.49±529.08	2512.53±837.34	1.573	0.00000	1.001	0.01185	1.492	0.00001	0.00001	pubmed
ENSMUSG00000021751	Acox2	70.18±11.28	145.72±13.26	173.06±15.68	192.56±32	1.054	0.00000	1.301	0.00000	1.453	0.00000	0.00000	pubmed
ENSMUSG00000025176	Hoga1	7.96±1.76	27.4±5.45	15.17±1.13	22.89±8.59	1.784	0.00000	0.932	0.04698	1.508	0.00003	0.00003	pubmed
ENSMUSG00000029195	Klb	10.3±0.31	22.91±7.9	20.5±5.24	21.2±10.55	1.153	0.00696	0.989	0.03405	1.054	0.00957	0.00957	pubmed
ENSMUSG00000040283	Btnl9	2.55±1.13	7.45±3.56	10.25±2.62	6.43±2.01	1.534	0.03551	1.988	0.00249	1.337	0.05139	0.05139	pubmed
ENSMUSG00000056035	Cyp3a11	316.42±51.16	1575.71±692.53	1734.75±1004.36	3006.13±1765.84	2.316	0.00167	2.455	0.00124	3.248	0.00000	0.00000	pubmed

Appendix Table S5

List of genes derived from the RNASEQ experiment shown in Fig. 2. Definitions of the categories see Fig. 2.

Ensgene	Symbol	HP	HD	ZP	ZD	LFC HD-HP	FDR HD-HP	LFC ZP-HP	FDR ZP-HP	LFC ZD-HP	FDR ZD-HP	Ratio	Links
ENSMUSG00000003134	Tbc1d8	175.78±14.58	319.78±50.73	209.41±2.51	472.72±48.04	0.863	0.00002	0.253	0.41952	1.427	0.00000	0.00000	pubmed
ENSMUSG00000004655	Aqp1	1316.18±150.99	3914.82±236.7	1910.29±367.36	5564.57±82.39	1.572	0.00000	0.537	0.00056	2.080	0.00000	0.00000	pubmed
ENSMUSG00000025978	Rftn2	40.24±2.33	91.99±8.54	58.62±7.15	133.47±6.81	1.193	0.00000	0.543	0.06570	1.730	0.00000	0.00000	pubmed
ENSMUSG00000026473	Glul	912.92±119.01	2640.61±157.95	1029.23±149.46	3840.62±307.12	1.533	0.00000	0.173	0.44929	2.073	0.00000	0.00000	pubmed
ENSMUSG00000028982	Slc25a33	41.02±2.95	140.24±4.11	42.16±3.28	354.75±38.8	1.774	0.00000	0.040	0.96232	3.113	0.00000	0.00000	pubmed
ENSMUSG00000029999	Tgfa	265.66±37.13	875.77±56.05	332.48±12.26	1356.1±247.04	1.721	0.00000	0.323	0.15390	2.351	0.00000	0.00000	pubmed
ENSMUSG00000030259	Rassf8	58.84±7.44	115.88±14.98	97.41±18.15	215.38±21.27	0.977	0.00000	0.724	0.00120	1.870	0.00000	0.00000	pubmed
ENSMUSG00000031725	Ces1f	555.52±123.77	1057.47±56.18	882.02±98.7	1624.99±95.96	0.929	0.00000	0.667	0.00006	1.549	0.00000	0.00000	pubmed
ENSMUSG00000031891	Hsd11b2	203.58±38.4	582.72±23.86	310.3±71.31	887.44±100.2	1.517	0.00000	0.608	0.04618	2.124	0.00000	0.00000	pubmed
ENSMUSG00000032226	Gcnt3	604.32±33.3	1142.07±50.91	879.46±120.22	1972.81±509.14	0.918	0.00007	0.541	0.05674	1.707	0.00000	0.00000	pubmed
ENSMUSG00000032350	Gclc	442.48±13.86	877.85±105.79	625.95±5.95	1429.59±261.08	0.988	0.00000	0.500	0.00523	1.692	0.00000	0.00000	pubmed
ENSMUSG00000032372	Plscr2	11.01±2.05	20.23±0.86	16.76±1.19	33.83±7.39	0.878	0.02512	0.608	0.19234	1.613	0.00000	0.00000	pubmed
ENSMUSG00000042116	Vwa1	144.45±11.59	375.1±53	247.77±23.4	590.48±131.16	1.377	0.00000	0.779	0.00519	2.031	0.00000	0.00000	pubmed
ENSMUSG00000044033	Ccdc141	27.53±4.72	70.76±2.67	35.68±5.92	134.36±15.92	1.359	0.00000	0.374	0.28492	2.285	0.00000	0.00000	pubmed
ENSMUSG00000061878	Sphk1	13.36±3.31	27.4±3.07	18.59±0.67	67.52±4.51	1.037	0.01547	0.478	1.00000	2.338	0.00000	0.00000	pubmed
ENSMUSG00000070644	Etnk2	3.06±1.36	10.34±5.09	5.26±2.35	20.26±5.5	1.769	0.00332	0.778	0.39325	2.723	0.00000	0.00000	pubmed
ENSMUSG00000094091	Gm21885	101.42±7.7	246.05±24.67	165.82±9.13	356.85±71.98	1.279	0.00000	0.709	0.00585	1.815	0.00000	0.00000	pubmed

Appendix Table S6

List of genes derived from the RNASEQ experiment shown in Fig. 2. Definitions of the categories see Fig. 2.

Ensgene	Symbol	HP	HD	ZP	ZD	LFC HD-HP	FDR HD-HP	LFC ZP-HP	FDR ZP-HP	LFC ZD-HP	FDR ZD-HP	Ratio	Links
ENSMUSG00000000686	Abhd15	17.99±3.15	56.6±8.55	14.09±1.99	56.97±10.5	1.653	0.00000	-0.357	0.52326	1.665	0.00000	0.00000	pubmed
ENSMUSG00000000731	Aire	0.95±1.35	122.22±46.2	1.1±1.55	100.26±18.84	7.018	0.00000	0.245	1.00000	6.733	0.00000	0.00000	pubmed
ENSMUSG00000000732	Icosl	153.7±50.11	1120.98±75.49	137.62±53.71	1105.89±319.37	2.867	0.00000	-0.159	0.82683	2.847	0.00000	0.00000	pubmed
ENSMUSG00000001095	Slc13a2	1684.39±101.52	3189.23±216.29	1692.71±388.28	3134.3±617.81	0.921	0.00064	0.007	0.99380	0.896	0.00055	0.00055	pubmed
ENSMUSG00000001225	Slc26a3	1010.21±142.54	1957.32±214.22	850.41±176.07	1480.45±358.38	0.954	0.00055	-0.249	0.57229	0.551	0.05937	0.05937	pubmed
ENSMUSG00000001240	Ramp2	48.32±7.47	93.31±10.46	63.03±7.75	103.44±7.1	0.952	0.00000	0.385	0.17634	1.101	0.00000	0.00000	pubmed
ENSMUSG00000001249	Hpn	9.56±1.96	18.34±7.4	9.96±1.92	11.54±5.95	0.936	0.01757	0.056	0.95368	0.293	0.56642	0.56642	pubmed
ENSMUSG00000001542	Ell2	705.8±19.23	1643.59±89.3	727.24±20.56	1637.17±301.78	1.220	0.00000	0.043	0.92136	1.214	0.00000	0.00000	pubmed
ENSMUSG00000001627	Ifrd1	418.6±80.24	881.56±35.44	505.64±163.83	794.57±158.55	1.074	0.00012	0.272	0.54153	0.924	0.00080	0.00080	pubmed
ENSMUSG00000002289	Angptl4	252.05±122.47	2574.17±404.91	376.55±184.9	3581.37±610.75	3.353	0.00000	0.579	0.52716	3.829	0.00000	0.00000	pubmed
ENSMUSG00000002769	Gnmt	4.43±0.78	29.44±16.71	2.28±1.44	35.81±6.51	2.730	0.00000	-0.969	0.41070	3.013	0.00000	0.00000	pubmed
ENSMUSG00000002831	Plin4	100.23±15.99	351.14±79.87	170.33±81.08	342.13±99.16	1.809	0.00000	0.765	0.13189	1.772	0.00000	0.00000	pubmed
ENSMUSG00000002944	Cd36	219.26±57.79	482.47±110.95	340.79±124.72	377.11±123.2	1.138	0.01410	0.636	0.28302	0.783	0.08996	0.08996	pubmed
ENSMUSG00000003134	Tbc1d8	175.78±14.58	319.78±50.73	209.41±2.51	472.72±48.04	0.863	0.00002	0.253	0.41952	1.427	0.00000	0.00000	pubmed
ENSMUSG00000003178	Mical3	46.99±3.58	93.38±9.72	51.71±1.81	89.86±7.2	0.990	0.00000	0.137	0.73772	0.933	0.00000	0.00000	pubmed
ENSMUSG00000003271	Sult2b1	966.04±36.8	1860.48±98.52	985.84±73.24	1742.12±401.28	0.946	0.00000	0.029	0.95686	0.850	0.00003	0.00003	pubmed
ENSMUSG00000003420	Fcgrt	641.62±93.92	1182.66±48.51	650.77±71.47	1380.85±208.09	0.882	0.00000	0.020	0.96204	1.106	0.00000	0.00000	pubmed
ENSMUSG00000003477	Inmt	1.41±0.03	6.61±5.49	3.91±2.57	3.16±2.38	2.243	0.00977	1.458	0.18866	1.210	0.22714	0.22714	pubmed
ENSMUSG00000003526	Prodh	379.13±27.87	939.91±109.61	288.49±39.19	781.76±30.26	1.310	0.00002	-0.394	0.39941	1.044	0.00069	0.00069	pubmed
ENSMUSG00000003882	Il7r	56.47±24.68	121.93±11.89	52.99±13.06	110.47±8.1	1.112	0.00436	-0.089	0.91458	0.970	0.01002	0.01002	pubmed
ENSMUSG00000003955	Fam162a	288.09±45.14	555.65±35.68	253.73±45.21	438.56±48.76	0.948	0.00000	-0.183	0.48562	0.606	0.00018	0.00018	pubmed
ENSMUSG00000004031	Brinp2	16.11±2.93	44.4±13.33	18.43±3.4	52.28±7.65	1.462	0.00119	0.191	1.00000	1.699	0.00004	0.00004	pubmed
ENSMUSG00000004328	Hif3a	17.07±2.64	424.98±41.36	26.39±13.38	483.03±60.69	4.637	0.00000	0.626	0.24277	4.822	0.00000	0.00000	pubmed
ENSMUSG00000004655	Aqp1	1316.18±150.99	3914.82±236.7	1910.29±367.36	5564.57±82.39	1.572	0.00000	0.537	0.00056	2.080	0.00000	0.00000	pubmed
ENSMUSG00000005514	Por	486.88±36.08	1181.84±104.09	571.22±109.26	1188.02±238.77	1.279	0.00000	0.230	0.45439	1.287	0.00000	0.00000	pubmed
ENSMUSG00000005836	Gata6	524.17±11.05	1118.04±48.8	491.45±64.73	862.84±230.81	1.093	0.00000	-0.093	0.83819	0.719	0.00154	0.00154	pubmed
ENSMUSG00000006050	Sra1	476.39±17.76	994.65±77.03	500.65±44.63	1139.17±105.63	1.062	0.00000	0.071	0.79372	1.258	0.00000	0.00000	pubmed
ENSMUSG00000006221	Hspb7	37.38±6.25	75.8±5.6	50.87±18.4	104.18±18.41	1.019	0.01652	0.443	0.45229	1.479	0.00005	0.00005	pubmed
ENSMUSG00000006542	Prkag3	1.66±1.47	20.24±4.83	3.39±0.48	23.35±5.71	3.621	0.00001	1.046	1.00000	3.827	0.00000	0.00000	pubmed
ENSMUSG00000006958	Chrd	12.89±3.58	27.86±9.55	9.53±5	27.48±4.99	1.114	0.02209	-0.435	1.00000	1.094	0.01416	0.01416	pubmed
ENSMUSG00000007097	Atp1a2	115.29±18.25	297.94±90.41	162.36±42.76	197.94±76.64	1.370	0.00104	0.494	0.41886	0.781	0.07785	0.07785	pubmed
ENSMUSG00000007682	Dio2	3.5±0.97	12.49±2.55	4.73±1.87	16.62±5.66	1.833	0.01633	0.436	1.00000	2.248	0.00071	0.00071	pubmed
ENSMUSG00000008540	Mgst1	1933.79±311.51	4038.64±250.69	1939.12±127.97	3837.88±115.29	1.063	0.00000	0.004	0.99341	0.989	0.00000	0.00000	pubmed
ENSMUSG00000008730	Hipk1	1351.71±49.39	3397.31±127.54	1328.44±35.67	3013.52±133.19	1.330	0.00000	-0.025	0.90657	1.157	0.00000	0.00000	pubmed
ENSMUSG00000008734	Gprc5b	43.23±8.14	81.58±14.39	60.64±6.41	115.24±11.72	0.918	0.01058	0.489	0.29781	1.417	0.00000	0.00000	pubmed
ENSMUSG00000008845	Cd163	35±19.07	91.88±20.41	30.55±7.85	68.52±18.99	1.394	0.00025	-0.197	0.79372	0.972	0.01327	0.01327	pubmed
ENSMUSG00000010122	Slc47a1	91.79±42.39	183.73±22.68	152.63±20.09	282.61±93.81	1.000	0.01434	0.733	0.12305	1.621	0.00000	0.00000	pubmed
ENSMUSG00000011034	Slc5a1	8470.27±319.08	16733.51±995.05	5876.56±466.92	12722.37±3481.73	0.982	0.00005	-0.527	0.08489	0.587	0.02171	0.02171	pubmed
ENSMUSG00000011171	Vipr2	18.75±1.85	48.66±3.51	16.44±4.89	57.11±10.08	1.377	0.00044	-0.185	1.00000	1.609	0.00001	0.00001	pubmed
ENSMUSG00000013076	Amotl1	158.05±9.46	295.55±23.09	188.13±23.46	312.83±54.32	0.903	0.00000	0.251	0.29973	0.987	0.00000	0.00000	pubmed
ENSMUSG00000014353	Tmem87b	827.74±35.05	1910.1±40.02	898.23±24.25	1859.43±280.95	1.206	0.00000	0.118	0.60402	1.167	0.00000	0.00000	pubmed
ENSMUSG00000014361	Mertk	646.59±44.08	1325.31±101.59	709.34±54.38	1131.37±322.75	1.035	0.00000	0.134	0.72890	0.807	0.00009	0.00009	pubmed
ENSMUSG00000014813	Stc1	8.89±0.28	20.75±2.28	18.86±9.01	44.93±6.93	1.222	0.04821	1.083	1.00000	2.336	0.00000	0.00000	pubmed
ENSMUSG00000015312	Gadd45b	136.6±30.65	583.04±80.97	96.78±20.31	598.3±147.58	2.094	0.00009	-0.497	0.57134	2.131	0.00003	0.00003	pubmed
ENSMUSG00000015342	Xk	468.31±21.1	869.54±33.69	649.31±93.08	1088.55±139.34	0.893	0.00000	0.471	0.02673	1.217	0.00000	0.00000	pubmed
ENSMUSG00000015468	Notch4	58.07±1.25	163.81±11.58	63.41±10.67	159.66±9.3	1.497	0.00000	0.125	0.71996	1.461	0.00000	0.00000	pubmed
ENSMUSG00000015850	Adamtsl4	66.88±10.77	130.86±12.2	75.03±3.37	144.44±7.83	0.970	0.00000	0.167	0.64854	1.113	0.00000	0.00000	pubmed
ENSMUSG00000017309	Cd300lg	31.21±8.27	60.05±9.28	49.29±5.15	63.08±1.98	0.948	0.00003	0.660	0.01161	1.018	0.00000	0.00000	pubmed
ENSMUSG00000017418	Arl5b	275.33±17.76	956.9±85.65	296.82±16.84	984.6±148.94	1.797	0.00000	0.108	0.70325	1.838	0.00000	0.00000	pubmed
ENSMUSG00000017688	Hnf4g	1919.49±257.15	5098.15±236.09	1872.3±107.5	5072.91±1254.81	1.409	0.00000	-0.036	0.94985	1.402	0.00000	0.00000	pubmed
ENSMUSG00000017707	Serinc3	7643.49±130.36	13680.59±454.65	8050.21±209.66	13782.91±1001.85	0.840	0.00000	0.075	0.52020	0.851	0.00000	0.00000	pubmed
ENSMUSG00000017718	Afmid	13.53±2.66	33.25±2.89	21.04±1.94	33.04±5.66	1.294	0.00007	0.635	0.15390	1.285	0.00004	0.00004	pubmed
ENSMUSG00000018846	Pank3	2346.91±385.8	4125.63±193.75	2618.65±164.42	4219.23±558.11	0.814	0.00000	0.158	0.53144	0.846	0.00000	0.00000	pubmed
ENSMUSG00000018900	Slc22a5	641.09±15.16	1792.41±173.07	620.34±35.24	1415.47±353.54	1.483	0.00000	-0.047	0.91979	1.142	0.00000	0.00000	pubmed
ENSMUSG00000018906	P4ha2	25.04±1.14	44.17±6.76	27.64±1.36	53.33±6.46	0.819	0.00923	0.143	0.80462	1.094	0.00006	0.00006	pubmed
ENSMUSG00000019368	Sec14l4	0.69±0.56	7.17±3.33	1.38±0.99	6.68±2.5	3.355	0.00211	0.954	0.64047	3.267	0.00167	0.00167	pubmed
ENSMUSG00000019577	Pdk4	107.42±90.78	496.68±145.12	78.04±11.31	646.13±91.84	2.209	0.02580	-0.461	0.77948	2.589	0.00330	0.00330	pubmed
ENSMUSG00000019970	Sgk1	2121.76±155.61	11729.94±193.1	3235.52±195.22	13714.45±2449.82	2.467	0.00000	0.609	0.00048	2.692	0.00000	0.00000	pubmed
ENSMUSG00000020010	Vnn3	5.7±4.2	16.23±4.55	3.4±0.08	13.94±2.22	1.530	0.00042	-0.725	0.39447	1.318	0.00231	0.00231	pubmed
ENSMUSG00000020017	Hal	1.88±1.21	8.73±7.85	2.72±0.06	15.33±3.71	2.229	0.00195	0.539	0.69794	3.029	0.00000	0.00000	pubmed
ENSMUSG00000020023	Tmcc3	1621.52±19.83	4347.11±163.57	1305.41±108.75	3352.55±1123.03	1.423	0.00000	-0.313	0.37434	1.048	0.00000	0.00000	pubmed
ENSMUSG00000020107	Anapc16	253.13±22.26	483.56±16.09	256.26±17.79	519.05±27.38	0.934	0.00000	0.018	0.95746	1.036	0.00000	0.00000	pubmed
ENSMUSG00000020108	Ddit4	231.45±58.34	4242.34±111.56	223.83±25	4490.81±333.43	4.197	0.00000	-0.048	0.93358	4.279	0.00000	0.00000	pubmed
ENSMUSG00000020123	Avpr1a	15.52±3.82	33.75±4.5	13.19±4.68	49.25±4.47	1.126	0.00174	-0.234	0.73661	1.671	0.00000	0.00000	pubmed
ENSMUSG00000020176	Grb10	92.11±11.17	173.7±11.55	109.38±10.59	194.83±39.79	0.915	0.00004	0.248	0.48404	1.082	0.00000	0.00000	pubmed
ENSMUSG00000020268	Lyrm7	22±0.11	63.6±9.37	18.82±1.81	55.29±12.91	1.531	0.00000	-0.226	0.72223	1.326	0.00002	0.00002	pubmed
ENSMUSG00000020300	Cpeb4	716.42±28.54	1323.33±26.95	650.85±34.02	1175.58±218.35	0.885	0.00003	-0.139	0.72393	0.714	0.00085	0.00085	pubmed
ENSMUSG00000020303	Stc2	25.89±4.39	74.44±10.79	29.89±4.36	73.71±6.65	1.521	0.00000	0.205	1.00000	1.508	0.00000	0.00000	pubmed
ENSMUSG00000020400	Tnip1	772.66±66.16	1636.97±101.49	704.78±35.12	1218.53±225.03	1.083	0.00000	-0.133	0.67846	0.657	0.00026	0.00026	pubmed
ENSMUSG00000020423	Btg2	904.03±165.57	1829.62±130.23	1065.78±114.33	2554.34±332.91	1.017	0.00656	0.238	0.69466	1.499	0.00001	0.00001	pubmed
ENSMUSG00000020838	Slc6a4	267.92±28.62	469.36±43.85	307.93±28.49	523.93±24.77	0.809	0.00000	0.200	0.41998	0.967	0.00000	0.00000	pubmed

Ensgene	Symbol	HP	HD	ZP	ZD	LFC HD-HP	FDR HD-HP	LFC ZP-HP	FDR ZP-HP	LFC ZD-HP	FDR ZD-HP	Ratio	Links
ENSMUSG00000020848	Doc2b	11.41±3.09	73.98±16.84	15.43±0.92	107.15±16.75	2.692	0.00000	0.430	0.52056	3.226	0.00000	0.00000	pubmed
ENSMUSG00000020893	Per1	127.63±7.26	540.65±22.93	127.12±2.88	576.68±41.06	2.083	0.00000	-0.006	0.99684	2.176	0.00000	0.00000	pubmed
ENSMUSG00000020937	Plcd3	15.23±3	50.01±7.15	17.42±2.78	42.21±3.49	1.716	0.00000	0.196	1.00000	1.473	0.00003	0.00003	pubmed
ENSMUSG00000021069	Pygl	81.11±26.64	141.6±17.83	103.52±14.33	105.21±42.02	0.805	0.01624	0.353	0.44456	0.380	0.29461	0.29461	pubmed
ENSMUSG00000021095	Gsc	0.48±0.34	44.95±6.59	0.46±0.33	88.83±23.55	6.584	0.00000	-0.046	1.00000	7.566	0.00000	0.00000	pubmed
ENSMUSG00000021118	Plek2	200.42±3.57	459.57±81.52	256.77±39.38	579.98±127.19	1.197	0.00000	0.357	0.33840	1.532	0.00000	0.00000	pubmed
ENSMUSG00000021120	Pigh	166.64±17.79	458.12±27.46	180.8±9.58	474.57±58.02	1.459	0.00000	0.117	0.71517	1.510	0.00000	0.00000	pubmed
ENSMUSG00000021238	Aldh6a1	236.82±26.44	427.94±59.45	333.57±52.49	505.08±75.29	0.853	0.00000	0.493	0.01576	1.093	0.00000	0.00000	pubmed
ENSMUSG00000021265	Slc25a29	18.42±4.27	41.82±3.99	23.47±5.49	43.41±2.17	1.180	0.00178	0.344	0.57222	1.233	0.00047	0.00047	pubmed
ENSMUSG00000021285	Ppp1r13b	449.86±25.52	954.64±78.17	478.65±18.97	968.73±182.66	1.085	0.00000	0.089	0.79463	1.106	0.00000	0.00000	pubmed
ENSMUSG00000021286	Zfyve21	328.05±23.63	759.91±37.27	385.89±34.72	828.94±78.62	1.212	0.00000	0.234	0.30959	1.337	0.00000	0.00000	pubmed
ENSMUSG00000021360	Gcnt2	498.68±59.2	1143.3±77.41	436.04±7.14	1084.4±262.72	1.197	0.00000	-0.194	0.57540	1.120	0.00000	0.00000	pubmed
ENSMUSG00000021364	Elovl2	3.72±1.36	14.3±9.75	2.49±0.29	16.3±6.11	1.940	0.00011	-0.587	0.60152	2.139	0.00000	0.00000	pubmed
ENSMUSG00000021453	Gadd45g	174.78±50.74	800.19±121.86	126.77±35.26	1054.07±196.75	2.195	0.00000	-0.463	0.50943	2.593	0.00000	0.00000	pubmed
ENSMUSG00000021492	F12	0.69±0.56	5.29±5.79	0.22±0.31	3.38±3.16	2.922	0.01202	-1.631	0.61916	2.319	0.04613	0.04613	pubmed
ENSMUSG00000021612	Slc6a18	27.14±1.03	68.2±7.64	16.92±3.58	43.27±20.64	1.329	0.00797	-0.680	1.00000	0.669	0.21797	0.21797	pubmed
ENSMUSG00000021750	Fam107a	15.75±4.28	100.37±9.26	16.6±2.53	124.53±5.08	2.677	0.00000	0.077	0.91345	2.989	0.00000	0.00000	pubmed
ENSMUSG00000021765	Fst	29.79±8.97	84.17±7.66	44.64±9.3	115.16±10.52	1.495	0.00000	0.578	0.18149	1.948	0.00000	0.00000	pubmed
ENSMUSG00000021943	Gdf10	10.53±1.47	25.03±7.32	20.79±8.9	25.01±8.27	1.249	0.01099	0.977	0.08141	1.254	0.00574	0.00574	pubmed
ENSMUSG00000022018	Rgcc	30.23±1.69	114.06±13.23	40.66±4.36	110.88±19.87	1.917	0.00000	0.427	0.31787	1.877	0.00000	0.00000	pubmed
ENSMUSG00000022304	Dpys	0.46±0.64	3.7±4.67	0.23±0.33	2.7±1.74	2.992	0.04807	-1.046	0.79387	2.557	0.07437	0.07437	pubmed
ENSMUSG00000022383	Ppara	185.5±35.03	668.3±28.2	170.62±8.54	688.92±105.33	1.849	0.00000	-0.121	0.72890	1.893	0.00000	0.00000	pubmed
ENSMUSG00000022464	Slc38a4	7.23±1.16	22.01±16.48	9.5±1.94	45.09±11.93	1.605	0.00432	0.393	0.68570	2.640	0.00000	0.00000	pubmed
ENSMUSG00000022512	Cldn1	10.28±2.26	31.85±12.78	10.46±3.36	32±20.16	1.631	0.01094	0.023	0.99166	1.643	0.00569	0.00569	pubmed
ENSMUSG00000022579	Gpihbp1	13.87±5.85	24.2±3.09	17.88±4.16	27.34±6.05	0.809	0.02055	0.374	0.44156	0.993	0.00090	0.00090	pubmed
ENSMUSG00000022602	Arc	4.94±1.23	25.8±9.05	4.52±1.33	31.83±6.48	2.389	0.00009	-0.122	1.00000	2.690	0.00000	0.00000	pubmed
ENSMUSG00000022684	Bfar	411.22±10.23	782.72±64.73	493.15±43.97	818.45±120.77	0.929	0.00000	0.262	0.18601	0.992	0.00000	0.00000	pubmed
ENSMUSG00000022766	Serpind1	0.46±0.64	4.49±4.67	0.69±0.56	5.37±3.47	3.273	0.02306	0.539	0.86560	3.556	0.00558	0.00558	pubmed
ENSMUSG00000022791	Tnk2	285.16±24.46	972.81±135.34	257.41±24.04	873.04±72.65	1.771	0.00000	-0.147	0.59611	1.614	0.00000	0.00000	pubmed
ENSMUSG00000022838	Eaf2	45.87±1.86	87.19±10.15	29.37±4.46	60.77±3.18	0.927	0.00031	-0.641	0.06113	0.406	0.17069	0.17069	pubmed
ENSMUSG00000022871	Fetub	1.41±0.03	7.68±7.48	1.56±1.23	3.39±2.92	2.458	0.00272	0.175	0.93940	1.318	0.17146	0.17146	pubmed
ENSMUSG00000022893	Adamts1	102.89±58.7	192.15±9.88	69.57±23.26	277.4±39.09	0.902	0.03377	-0.564	0.29196	1.432	0.00005	0.00005	pubmed
ENSMUSG00000023067	Cdkn1a	1654.27±71.43	5381.14±268.86	1591.27±114.63	5287.75±986.68	1.702	0.00000	-0.056	0.92412	1.676	0.00000	0.00000	pubmed
ENSMUSG00000023073	Slc10a2	3020.5±179.02	5762.91±120.6	2803.63±317.41	6043.55±1856.95	0.932	0.00028	-0.108	0.83013	1.001	0.00004	0.00004	pubmed
ENSMUSG00000023150	Ivns1abp	3492.62±243.92	6239.26±257.81	3442.46±230.35	6018.48±464.26	0.837	0.00000	-0.021	0.94584	0.785	0.00000	0.00000	pubmed
ENSMUSG00000023206	Il15ra	85.04±5.89	278.63±4.15	62.42±4.13	194.04±29.84	1.713	0.00000	-0.446	0.13135	1.191	0.00000	0.00000	pubmed
ENSMUSG00000023243	Kcnk5	574.11±23.33	1306.65±11.09	534.43±118.42	1102.11±201.45	1.186	0.00001	-0.103	0.84833	0.941	0.00035	0.00035	pubmed
ENSMUSG00000023262	Acy1	445.36±29.85	958.71±10.16	451.52±51.97	948.23±65.02	1.106	0.00000	0.019	0.95861	1.090	0.00000	0.00000	pubmed
ENSMUSG00000023915	Tnfrsf21	258.5±15.92	636.93±48.9	307.42±35.11	730.04±162.34	1.301	0.00000	0.250	0.41140	1.497	0.00000	0.00000	pubmed
ENSMUSG00000023951	Vegfa	712.87±126.02	1263.75±145.41	565.96±153.58	1086.18±290.58	0.826	0.00439	-0.333	0.41736	0.607	0.03521	0.03521	pubmed
ENSMUSG00000024039	Cbs	16.81±2.28	37.25±6.44	26.58±3.46	47.56±11.08	1.145	0.00002	0.655	0.05309	1.503	0.00000	0.00000	pubmed
ENSMUSG00000024136	Dnase1l2	10.08±3.24	40.19±5.01	9.07±0.62	50.6±7.13	1.997	0.00000	-0.151	1.00000	2.327	0.00000	0.00000	pubmed
ENSMUSG00000024222	Fkbp5	748.53±75.22	7694.77±587.44	728.83±56.84	7682.37±553.52	3.362	0.00000	-0.039	0.94239	3.359	0.00000	0.00000	pubmed
ENSMUSG00000024395	Lims2	94.26±15.75	170.79±22.22	119.64±23.69	215.04±15.16	0.856	0.00000	0.341	0.19223	1.189	0.00000	0.00000	pubmed
ENSMUSG00000024515	Smad4	1021.63±117.13	2070.83±38.05	771.08±27.93	1499.53±146.8	1.020	0.00000	-0.406	0.00301	0.554	0.00000	0.00000	pubmed
ENSMUSG00000024521	Pmaip1	434.3±17.34	2524.35±309.19	356.27±21.34	3365.7±1295.17	2.539	0.00000	-0.286	0.56263	2.954	0.00000	0.00000	pubmed
ENSMUSG00000024575	Pde6a	7.48±0.51	41.74±8.06	3.37±1.38	23.85±9.48	2.480	0.00001	-1.143	1.00000	1.671	0.00454	0.00454	pubmed
ENSMUSG00000024597	Slc12a2	2469.3±444.69	4529.97±217.04	2977.85±135.14	5010.15±315.27	0.875	0.00000	0.270	0.27199	1.021	0.00000	0.00000	pubmed
ENSMUSG00000024679	Ms4a6d	46.55±3.27	98.86±14.77	35.43±4.95	95.53±3.55	1.087	0.00000	-0.396	0.22301	1.038	0.00000	0.00000	pubmed
ENSMUSG00000024780	Cdc37l1	177.79±17.11	900.79±82	163.12±17.72	934.25±179.02	2.340	0.00000	-0.126	0.68310	2.393	0.00000	0.00000	pubmed
ENSMUSG00000024827	Gldc	0.92±0.85	6.38±1.68	2.27±0.36	8.71±1.39	2.770	0.00569	1.276	0.40269	3.226	0.00030	0.00030	pubmed
ENSMUSG00000024892	Pcx	357.07±110.28	670.35±130.35	479±193.97	428.48±134.05	0.909	0.01624	0.424	0.40611	0.264	0.56321	0.56321	pubmed
ENSMUSG00000024924	Vldlr	57.32±2.19	151.54±22.81	74.08±2.64	210.16±32.95	1.403	0.00009	0.370	0.51880	1.874	0.00000	0.00000	pubmed
ENSMUSG00000024975	Pdcd4	622.21±52.5	1240.28±46.93	652.99±14.81	1248.5±92.02	0.995	0.00000	0.070	0.76743	1.005	0.00000	0.00000	pubmed
ENSMUSG00000024990	Rbp4	32.37±8.49	80.77±34.62	43.33±11.65	56.07±30.9	1.320	0.01230	0.420	0.60079	0.797	0.14332	0.14332	pubmed
ENSMUSG00000025190	Got1	534.11±13.16	1284.55±135.38	438.11±43.89	1247.5±309.95	1.266	0.00000	-0.286	0.36487	1.224	0.00000	0.00000	pubmed
ENSMUSG00000025192	Entpd7	240.04±36.24	602.47±37.98	202.74±44.81	453.2±123.35	1.327	0.00001	-0.244	0.63570	0.916	0.00259	0.00259	pubmed
ENSMUSG00000025195	Dnmbp	548.23±10.48	1074.84±37.37	591.63±42.33	982.1±135.05	0.971	0.00002	0.110	0.80499	0.841	0.00017	0.00017	pubmed
ENSMUSG00000025372	Baiap2	49.59±7.64	86.39±10.41	57.27±5.44	113.22±8.84	0.801	0.00177	0.208	0.61803	1.191	0.00000	0.00000	pubmed
ENSMUSG00000025479	Cyp2e1	67.93±29.48	274.66±175.86	93.13±67.97	130.38±123.51	2.016	0.04611	0.455	0.77934	0.942	0.37700	0.37700	pubmed
ENSMUSG00000025936	Gm4956	8.89±0.76	20.23±3.58	9.75±3.43	16.29±0.86	1.186	0.02442	0.133	1.00000	0.873	0.09428	0.09428	pubmed
ENSMUSG00000025978	Rftn2	40.24±2.33	91.99±8.54	58.62±7.15	133.47±6.81	1.193	0.00000	0.543	0.06570	1.730	0.00000	0.00000	pubmed
ENSMUSG00000026073	Il1r2	29.12±10.77	57.44±4.56	18.69±5.44	50.69±4.72	0.981	0.02624	-0.635	1.00000	0.802	0.05673	0.05673	pubmed
ENSMUSG00000026090	2010300C02Rik	370.11±23.45	908.46±37.56	485.37±29.11	1119.87±116.02	1.295	0.00000	0.391	0.01490	1.597	0.00000	0.00000	pubmed
ENSMUSG00000026094	Stk17b	1722.11±335.48	4814.53±163.72	1673.22±229.41	3716.62±938.29	1.483	0.00000	-0.042	0.94125	1.110	0.00000	0.00000	pubmed
ENSMUSG00000026113	Inpp4a	223.51±24.8	554.62±44.17	250.68±4.09	625.58±42.68	1.310	0.00000	0.165	0.44492	1.483	0.00000	0.00000	pubmed
ENSMUSG00000026170	Cyp27a1	147.73±26.32	1042.69±119.71	191.54±17.73	1397.91±148.99	2.818	0.00000	0.374	0.06752	3.241	0.00000	0.00000	pubmed
ENSMUSG00000026380	Tfcp2l1	808.78±173.6	4449.57±337.74	890.26±28.37	4667.11±486.82	2.460	0.00000	0.138	0.66275	2.528	0.00000	0.00000	pubmed
ENSMUSG00000026473	Glul	912.92±119.01	2640.61±157.95	1029.23±149.46	3840.62±307.12	1.533	0.00000	0.173	0.44929	2.073	0.00000	0.00000	pubmed
ENSMUSG00000026579	F5	7.49±1.44	16.18±11.09	5.02±1.5	16.48±7.18	1.114	0.02739	-0.585	0.47538	1.153	0.01092	0.01092	pubmed
ENSMUSG00000026614	Slc30a10	374.94±28.47	762.45±76.6	233.18±48.22	603.24±313.93	1.024	0.00545	-0.685	0.12455	0.686	0.06612	0.06612	pubmed

Ensgene	Symbol	HP	HD	ZP	ZD	LFC HD-HP	FDR HD-HP	LFC ZP-HP	FDR ZP-HP	LFC ZD-HP	FDR ZD-HP	Ratio	Links
ENSMUSG00000026621	Marc1	8.2±3.21	18.32±6.67	11.46±5.6	11.8±5.75	1.164	0.00254	0.469	0.42111	0.551	0.22548	0.22548	pubmed
ENSMUSG00000026628	Atf3	204.81±40.59	434.08±65.92	195.85±49.1	338.11±70.46	1.083	0.00075	-0.064	0.93056	0.722	0.02891	0.02891	pubmed
ENSMUSG00000026657	Frmd4a	96.28±7.91	189.14±11.83	102.8±10.17	239.26±12.38	0.973	0.00000	0.094	0.79463	1.313	0.00000	0.00000	pubmed
ENSMUSG00000026712	Mrc1	145.19±25.91	272.52±30.5	130.85±14.85	231.48±35.43	0.909	0.00000	-0.150	0.64901	0.675	0.00026	0.00026	pubmed
ENSMUSG00000026796	Fam129b	361.26±34.36	645.98±71.89	378.7±31	740.78±73.66	0.838	0.00001	0.068	0.86535	1.036	0.00000	0.00000	pubmed
ENSMUSG00000026822	Lcn2	29.67±8.94	892.74±65.53	16.35±3.98	529.77±52.44	4.913	0.00000	-0.859	0.29345	4.160	0.00000	0.00000	pubmed
ENSMUSG00000026986	Hnmt	17.64±5.18	33.85±7.57	25.46±4.62	34.01±1.38	0.943	0.00692	0.531	0.24107	0.953	0.00325	0.00325	pubmed
ENSMUSG00000027111	Itga6	2871.33±108.27	5119.49±199.29	2232.71±152.94	4204.96±627.77	0.834	0.00000	-0.363	0.02857	0.550	0.00005	0.00005	pubmed
ENSMUSG00000027306	Nusap1	434.16±70.79	802.53±25.03	420.74±33.55	814.2±114.62	0.886	0.00069	-0.045	0.94184	0.908	0.00026	0.00026	pubmed
ENSMUSG00000027346	Gpcpd1	407.31±17.78	1404.6±132.7	334.54±31.22	1027.35±86.05	1.786	0.00000	-0.284	0.41944	1.335	0.00000	0.00000	pubmed
ENSMUSG00000027375	Mal	87.04±14.51	206.21±54.36	62.28±9.13	136.71±35.29	1.244	0.00053	-0.484	0.35979	0.649	0.09677	0.09677	pubmed
ENSMUSG00000027463	Slc52a3	1597.5±129.85	3207.31±187.41	1381.03±201.09	2196.6±668.81	1.006	0.00055	-0.210	0.66711	0.459	0.15605	0.15605	pubmed
ENSMUSG00000027488	Snta1	52.66±1.05	92.32±17.11	72.87±11.68	81.66±6.39	0.810	0.00042	0.468	0.10544	0.631	0.00639	0.00639	pubmed
ENSMUSG00000027534	Snx16	63.39±2.08	118.18±2.7	64.11±1.77	103.4±17.17	0.898	0.00003	0.016	0.98309	0.704	0.00124	0.00124	pubmed
ENSMUSG00000027630	Tbl1xr1	622.07±47.06	1236.43±58.66	554.23±37.83	1186.07±96.64	0.991	0.00000	-0.166	0.40043	0.931	0.00000	0.00000	pubmed
ENSMUSG00000027684	Mecom	162.13±10.43	344.51±38.48	151.7±13.71	297±30.94	1.087	0.00000	-0.097	0.79387	0.873	0.00000	0.00000	pubmed
ENSMUSG00000027690	Slc2a2	39.55±23.1	172.31±24.88	25.21±20.17	291.85±139.93	2.124	0.00293	-0.651	0.56040	2.884	0.00001	0.00001	pubmed
ENSMUSG00000027843	Ptpn22	168.38±42.25	515.29±70.36	107.85±32.35	353.14±8.41	1.614	0.00000	-0.641	0.07923	1.069	0.00011	0.00011	pubmed
ENSMUSG00000027845	Dclre1b	103.07±4.36	421.59±10.95	95.25±7.72	373.39±48.23	2.033	0.00000	-0.112	0.71584	1.856	0.00000	0.00000	pubmed
ENSMUSG00000027947	Il6ra	68.48±9.77	126.14±13.29	83.85±2.51	153.35±17.69	0.881	0.00013	0.291	0.40483	1.164	0.00000	0.00000	pubmed
ENSMUSG00000028016	Ints12	225.18±11.28	424.81±27.77	213.07±11.09	292.65±64.87	0.915	0.00000	-0.080	0.84200	0.376	0.08621	0.08621	pubmed
ENSMUSG00000028121	Bcar3	761.94±69.7	2404.45±122.97	707.46±18.11	2459.38±613.14	1.658	0.00000	-0.107	0.79365	1.690	0.00000	0.00000	pubmed
ENSMUSG00000028186	Uox	6.49±3.19	21.19±18.63	6.8±0.94	25.69±15.12	1.703	0.03969	0.062	0.97858	1.984	0.00596	0.00596	pubmed
ENSMUSG00000028211	Trp53inp1	955.03±53.64	3893.12±199.4	949.36±98.39	4215.57±333.86	2.027	0.00000	-0.008	0.98874	2.142	0.00000	0.00000	pubmed
ENSMUSG00000028334	Nans	819.77±81.3	1583.16±78.62	697.2±62.06	1591.51±154.03	0.949	0.00000	-0.234	0.28726	0.957	0.00000	0.00000	pubmed
ENSMUSG00000028359	Orm3	0.24±0.34	6.39±0.65	0.23±0.33	1.42±2.01	4.770	0.01681	-0.046	0.99684	2.670	0.22552	0.22552	pubmed
ENSMUSG00000028542	Slc6a9	95.66±11.79	354.8±27.96	93.37±7.21	327.34±48.88	1.892	0.00000	-0.034	0.95792	1.776	0.00000	0.00000	pubmed
ENSMUSG00000028713	Cyp4b1	222.83±62.69	416.68±45	372.76±60.42	604.51±169.97	0.903	0.00274	0.742	0.02664	1.439	0.00000	0.00000	pubmed
ENSMUSG00000028807	Zbtb8a	103.03±5.06	182.41±13.95	102.95±10.23	169.65±8.72	0.824	0.00000	-0.001	0.99995	0.719	0.00006	0.00006	pubmed
ENSMUSG00000028862	Map3k6	276.09±12.14	868.19±74.54	267.21±41.8	942.29±189.56	1.653	0.00000	-0.048	0.91950	1.771	0.00000	0.00000	pubmed
ENSMUSG00000029135	Fosl2	1763.73±194.73	3152.94±154.86	1953.22±202.35	2966.46±609.03	0.838	0.00162	0.147	0.75317	0.750	0.00358	0.00358	pubmed
ENSMUSG00000029167	Ppargc1a	146.92±11.03	288.17±33.74	255.2±13.14	365.4±41.32	0.972	0.00000	0.797	0.00017	1.314	0.00000	0.00000	pubmed
ENSMUSG00000029273	Sult1d1	1892.45±233.5	5486.63±106.54	2985.48±535.6	6658.56±988.11	1.536	0.00000	0.658	0.00885	1.815	0.00000	0.00000	pubmed
ENSMUSG00000029287	Tgfbr3	190.03±11.11	357.5±25.54	244.35±33.72	432.87±57.71	0.912	0.00000	0.363	0.09627	1.189	0.00000	0.00000	pubmed
ENSMUSG00000029335	Bmp3	249.2±36.62	554.13±59.35	279.4±19.55	553.01±57.17	1.152	0.00000	0.165	0.61504	1.149	0.00000	0.00000	pubmed
ENSMUSG00000029695	Aass	3.98±0.31	13.79±11.7	5±1.32	9.27±6.09	1.795	0.01936	0.327	0.82235	1.235	0.11345	0.11345	pubmed
ENSMUSG00000029722	Agfg2	162.91±11.89	346.48±1.46	245.72±24.53	415.83±15.69	1.088	0.00000	0.592	0.00288	1.351	0.00000	0.00000	pubmed
ENSMUSG00000029727	Cyp3a13	2632.87±76.64	5686.96±245.52	2639.05±655.35	4072.18±1308.78	1.111	0.00002	0.003	0.99913	0.629	0.02317	0.02317	pubmed
ENSMUSG00000029999	Tgfa	265.66±37.13	875.77±56.05	332.48±12.26	1356.1±247.04	1.721	0.00000	0.323	0.15390	2.351	0.00000	0.00000	pubmed
ENSMUSG00000030020	Prickle2	115.71±17.01	230.7±14.83	83.92±17.57	278.11±43.93	0.996	0.00003	-0.464	0.16406	1.266	0.00000	0.00000	pubmed
ENSMUSG00000030022	Adamts9	73.35±18.83	163.99±21.85	62.52±18.75	168.68±39.44	1.162	0.00132	-0.229	0.71907	1.203	0.00042	0.00042	pubmed
ENSMUSG00000030087	Klf15	27.09±3.51	177.33±23.34	31.53±12.13	238.49±38.85	2.709	0.00000	0.210	0.65264	3.138	0.00000	0.00000	pubmed
ENSMUSG00000030096	Slc6a6	2004.12±264.99	6511.48±433.26	2236.97±230.19	6685.01±653.25	1.700	0.00000	0.158	0.47432	1.738	0.00000	0.00000	pubmed
ENSMUSG00000030244	Gys2	1.17±0.32	5.03±4.29	0.93±1.31	1.42±2.01	2.110	0.03969	-0.367	0.88006	0.357	0.83365	0.83365	pubmed
ENSMUSG00000030278	Cidec	186.94±44.17	429.95±114.67	227.06±116.23	240.73±29.49	1.202	0.00702	0.280	0.69967	0.365	0.49693	0.49693	pubmed
ENSMUSG00000030428	Ttyh1	58.4±6.74	102.34±13.24	63.18±20.02	75.59±2.99	0.809	0.02206	0.111	0.86491	0.372	0.33278	0.33278	pubmed
ENSMUSG00000030474	Siglece	21.48±4.73	44.66±4.83	21.23±3.45	41.97±8.72	1.055	0.00107	-0.016	0.98828	0.969	0.00186	0.00186	pubmed
ENSMUSG00000030483	Cyp2b10	4.17±2.38	72.4±2.08	6.14±4.27	92.97±43.55	4.111	0.00000	0.548	0.66297	4.470	0.00000	0.00000	pubmed
ENSMUSG00000030545	Pex11a	281.51±80.09	606.84±44.05	199.8±14.96	550.59±80.23	1.109	0.00000	-0.495	0.06177	0.968	0.00000	0.00000	pubmed
ENSMUSG00000030650	Tmc5	2421.73±88.67	4974.29±295.19	2144.18±262.7	3812.92±990.9	1.038	0.00001	-0.176	0.66129	0.655	0.00617	0.00617	pubmed
ENSMUSG00000030660	Pik3c2a	1024.39±80.52	2226.27±87.4	869.28±75.41	2075.98±465.74	1.120	0.00000	-0.237	0.41176	1.019	0.00000	0.00000	pubmed
ENSMUSG00000030739	Myh14	5279.58±391.74	9204.63±539.24	6246.81±594.65	9033.75±1730.34	0.802	0.00000	0.243	0.34154	0.775	0.00000	0.00000	pubmed
ENSMUSG00000030747	Dgat2	1070.96±79.88	2243.53±131.94	910.54±262.32	1588.72±341.45	1.067	0.00000	-0.234	0.51886	0.569	0.01718	0.01718	pubmed
ENSMUSG00000030787	Lyve1	173.26±7.17	510.97±27.67	178.17±10.92	430.24±17.44	1.561	0.00000	0.040	0.89418	1.312	0.00000	0.00000	pubmed
ENSMUSG00000031016	Wee1	91.41±7.4	222.19±1.99	96.22±7.53	207.45±11.85	1.281	0.00000	0.074	0.84745	1.182	0.00000	0.00000	pubmed
ENSMUSG00000031216	Stard8	47.05±6.63	85.89±9.31	48.7±5.66	99.98±11.25	0.869	0.00000	0.050	0.91168	1.091	0.00000	0.00000	pubmed
ENSMUSG00000031286	Glt28d2	81.73±2.6	151.99±4.97	75.46±9.26	121.47±18.26	0.895	0.00002	-0.116	0.78192	0.570	0.01077	0.01077	pubmed
ENSMUSG00000031381	Piga	186.59±22.04	847.65±59.55	156.21±17.63	722.55±127.4	2.183	0.00000	-0.257	0.40998	1.952	0.00000	0.00000	pubmed
ENSMUSG00000031382	Asb11	0.69±0.56	37.75±9.89	1.58±0.64	38.41±11.83	5.751	0.00000	1.177	1.00000	5.774	0.00000	0.00000	pubmed
ENSMUSG00000031387	Renbp	28.32±1.52	55.6±6.16	37.24±3.37	37.36±6.96	0.973	0.00028	0.394	0.31204	0.404	0.20373	0.20373	pubmed
ENSMUSG00000031431	Tsc22d3	476.72±89.44	4149.31±395.43	432.15±39.24	4389.66±380.13	3.122	0.00000	-0.141	0.66608	3.203	0.00000	0.00000	pubmed
ENSMUSG00000031549	Ido2	0.69±0.56	4.51±1.96	0.89±0.82	3.17±2.01	2.688	0.02988	0.368	0.89607	2.211	0.06582	0.06582	pubmed
ENSMUSG00000031596	Slc7a2	38.18±12.95	99.09±18.72	35.22±5.26	100.31±4.88	1.377	0.00000	-0.118	0.84230	1.394	0.00000	0.00000	pubmed
ENSMUSG00000031604	Msmo1	1848.35±346.87	3644.1±168.12	2063.68±269.26	3418.08±817.55	0.979	0.00009	0.159	0.72627	0.887	0.00030	0.00030	pubmed
ENSMUSG00000031662	Snx20	182.43±37.35	373.16±42.57	171.7±24.9	362.93±36.96	1.033	0.00001	-0.087	0.85967	0.992	0.00001	0.00001	pubmed
ENSMUSG00000031725	Ces1f	555.52±123.77	1057.47±56.18	882.02±98.7	1624.99±95.96	0.929	0.00000	0.667	0.00006	1.549	0.00000	0.00000	pubmed
ENSMUSG00000031770	Herpud1	1057.23±40.04	4720.88±218.39	826.15±70.99	5017.03±332.26	2.159	0.00000	-0.356	0.21569	2.246	0.00000	0.00000	pubmed
ENSMUSG00000031775	Pllp	182.32±12.45	339.28±31.98	235.7±32.08	412.49±17.69	0.896	0.00000	0.370	0.16313	1.178	0.00000	0.00000	pubmed
ENSMUSG00000031780	Ccl17	5.61±2.08	18.08±2.51	2.01±1.4	13.8±2.07	1.687	0.01027	-1.466	1.00000	1.296	0.04698	0.04698	pubmed
ENSMUSG00000031825	Crispld2	205.19±11.67	461.75±22.03	232.23±56.61	556.94±87.73	1.170	0.00000	0.178	0.65537	1.441	0.00000	0.00000	pubmed
ENSMUSG00000031891	Hsd11b2	203.58±38.4	582.72±23.86	310.3±71.31	887.44±100.2	1.517	0.00000	0.608	0.04618	2.124	0.00000	0.00000	pubmed

Ensgene	Symbol	HP	HD	ZP	ZD	LFC HD-HP	FDR HD-HP	LFC ZP-HP	FDR ZP-HP	LFC ZD-HP	FDR ZD-HP	Ratio	Links
ENSMUSG00000032010	Usp2	266.03±53.23	583.29±23.65	200.78±42.31	534.59±51.17	1.132	0.00216	-0.406	0.45413	1.006	0.00489	0.00489	pubmed
ENSMUSG00000032079	Apoa5	0.46±0.64	6.86±9.15	0.91±0.34	6.1±4.07	3.886	0.03016	0.966	0.76629	3.721	0.02398	0.02398	pubmed
ENSMUSG00000032226	Gcnt3	604.32±33.3	1142.07±50.91	879.46±120.22	1972.81±509.14	0.918	0.00007	0.541	0.05674	1.707	0.00000	0.00000	pubmed
ENSMUSG00000032350	Gclc	442.48±13.86	877.85±105.79	625.95±5.95	1429.59±261.08	0.988	0.00000	0.500	0.00523	1.692	0.00000	0.00000	pubmed
ENSMUSG00000032372	Plscr2	11.01±2.05	20.23±0.86	16.76±1.19	33.83±7.39	0.878	0.02512	0.608	0.19234	1.613	0.00000	0.00000	pubmed
ENSMUSG00000032374	Plod2	81.8±6.48	144.29±7.14	119.98±12.89	174.61±6.73	0.818	0.00001	0.552	0.00794	1.094	0.00000	0.00000	pubmed
ENSMUSG00000032417	Rwdd2a	6.77±1.34	19.65±4.73	7.01±2.79	20.02±0.44	1.536	0.00959	0.050	1.00000	1.562	0.00432	0.00432	pubmed
ENSMUSG00000032470	Mras	45.58±9.67	98.74±1.83	54.88±1.2	130.83±14.23	1.118	0.00000	0.270	0.46075	1.525	0.00000	0.00000	pubmed
ENSMUSG00000032513	Gorasp1	157±25.84	296.36±19.45	164.88±19.19	334.94±22.02	0.917	0.00000	0.070	0.84679	1.093	0.00000	0.00000	pubmed
ENSMUSG00000032528	Vipr1	1077.04±114.71	2985.67±148.14	1016.33±42.78	3002.66±296.18	1.471	0.00000	-0.084	0.69697	1.479	0.00000	0.00000	pubmed
ENSMUSG00000032584	Mst1r	368.35±33.75	681.29±60.61	298.27±19.07	544.05±37.43	0.887	0.00000	-0.305	0.12993	0.562	0.00015	0.00015	pubmed
ENSMUSG00000032599	Ip6k2	149.89±8.96	386.76±45.04	164.9±8.41	546.26±6.28	1.367	0.00000	0.138	0.81224	1.865	0.00000	0.00000	pubmed
ENSMUSG00000032899	Styk1	1071.35±45.25	2278.97±83.7	1047.49±31.81	2169.21±362.17	1.089	0.00000	-0.033	0.93115	1.017	0.00000	0.00000	pubmed
ENSMUSG00000032911	Cspg4	60.5±6.1	120.88±17.61	96.7±26.1	172.54±0.84	0.998	0.00116	0.675	0.06508	1.511	0.00000	0.00000	pubmed
ENSMUSG00000032952	Ap4b1	210.4±0.95	471.97±12.95	182.56±7.24	393.11±30.64	1.166	0.00000	-0.205	0.17327	0.900	0.00000	0.00000	pubmed
ENSMUSG00000032978	Guca2b	1219.91±129.62	2389.17±163.3	1586.22±221.65	2699.18±634.48	0.970	0.00003	0.379	0.24782	1.146	0.00000	0.00000	pubmed
ENSMUSG00000033105	Lss	281.16±56.77	497.69±51.58	327.86±21.36	430.18±32.77	0.823	0.00089	0.221	0.57232	0.613	0.01389	0.01389	pubmed
ENSMUSG00000033453	Adamts15	121.98±11.18	394.46±19.09	86.01±9.71	450.07±43.86	1.693	0.00000	-0.506	0.02220	1.883	0.00000	0.00000	pubmed
ENSMUSG00000033590	Myo5c	480.96±55.11	1022.1±76.36	499.96±38.45	937.11±36.16	1.087	0.00000	0.055	0.85936	0.962	0.00000	0.00000	pubmed
ENSMUSG00000033618	Map3k13	205.32±10.1	360.85±32.8	231.54±24.05	265.74±64.61	0.813	0.00205	0.173	0.69466	0.371	0.20445	0.20445	pubmed
ENSMUSG00000033684	Qsox1	940.14±68.67	2238.13±134.74	887.99±81.86	1675.3±45.94	1.251	0.00000	-0.083	0.74869	0.833	0.00000	0.00000	pubmed
ENSMUSG00000033855	Ston1	121.56±14.32	359.1±19.74	124.54±14.32	432.63±64.18	1.562	0.00000	0.033	0.94239	1.832	0.00000	0.00000	pubmed
ENSMUSG00000033863	Klf9	380.68±41.77	811.4±27.05	379.26±43.3	868.94±52.91	1.092	0.00000	-0.005	0.99380	1.191	0.00000	0.00000	pubmed
ENSMUSG00000033985	Tesk2	347.24±30.32	909.02±29.19	351.56±28.88	963.8±123.3	1.389	0.00000	0.018	0.96987	1.473	0.00000	0.00000	pubmed
ENSMUSG00000033998	Kcnk1	511.98±46.1	999.13±83.45	478.33±34.06	1029.3±25.13	0.964	0.00000	-0.099	0.68343	1.007	0.00000	0.00000	pubmed
ENSMUSG00000034112	Atp2c2	147.59±9.51	541.85±59.46	153.83±20.27	513.1±67.52	1.876	0.00000	0.059	0.89672	1.798	0.00000	0.00000	pubmed
ENSMUSG00000034157	Cipc	281.08±37.52	577.82±39.85	330.98±31.5	695.73±8.05	1.039	0.00000	0.235	0.19790	1.307	0.00000	0.00000	pubmed
ENSMUSG00000034158	Lrrc58	433.34±4.57	774.17±6.01	483.55±33.5	1021.77±115.42	0.837	0.00000	0.158	0.42414	1.237	0.00000	0.00000	pubmed
ENSMUSG00000034258	Mfsd7c	55±11.2	160.08±12.43	66.47±43.86	135.82±56.31	1.541	0.03991	0.273	0.83177	1.304	0.06286	0.06286	pubmed
ENSMUSG00000034320	Slc26a2	4337.61±599.96	7684.58±222.63	4086.27±769.75	5641.98±1379.5	0.825	0.00144	-0.086	0.86560	0.379	0.18382	0.18382	pubmed
ENSMUSG00000034382	AI661453	552.36±13.42	988.28±40.23	524.18±50.13	954.15±143.72	0.839	0.00001	-0.076	0.84455	0.788	0.00002	0.00002	pubmed
ENSMUSG00000034614	Pik3ip1	52.18±4.9	102.21±33.39	69.73±9.62	95.02±2.36	0.969	0.00467	0.419	0.38130	0.864	0.00868	0.00868	pubmed
ENSMUSG00000034640	Tiparp	323.85±5.11	1394.44±125.75	312.39±24.26	1261.52±149.21	2.106	0.00000	-0.052	0.91979	1.962	0.00000	0.00000	pubmed
ENSMUSG00000034707	Gns	1227.99±95.16	2304.08±85.27	1004.21±46.52	1616.28±133.18	0.908	0.00000	-0.290	0.02120	0.396	0.00015	0.00015	pubmed
ENSMUSG00000034842	Art3	45.18±1.53	82.96±10.8	57.59±10.91	98.58±15.57	0.877	0.00136	0.348	0.37740	1.128	0.00000	0.00000	pubmed
ENSMUSG00000034926	Dhcr24	2382.92±250.12	10361.42±1200.55	3321.35±291.94	10894.84±1777.94	2.120	0.00000	0.479	0.01340	2.193	0.00000	0.00000	pubmed
ENSMUSG00000034936	Arl4d	106.71±12.95	611.79±29.46	151.43±12.61	871.81±65.43	2.519	0.00000	0.504	0.08776	3.029	0.00000	0.00000	pubmed
ENSMUSG00000035268	Pkig	336.15±41.96	617.35±23.12	330.32±19.81	615.31±28.66	0.878	0.00000	-0.024	0.93875	0.873	0.00000	0.00000	pubmed
ENSMUSG00000035270	Impg2	6.78±0.79	20.49±3.66	14.67±2.79	20.9±5.86	1.594	0.00117	1.116	0.05778	1.620	0.00046	0.00046	pubmed
ENSMUSG00000035529	Prdm4	365.71±31.44	814.95±70.55	376.02±31.13	840.76±119.12	1.156	0.00000	0.040	0.91786	1.201	0.00000	0.00000	pubmed
ENSMUSG00000035621	Midn	961.91±24.28	1974.38±129.38	959.11±37	1904.2±353.53	1.037	0.00000	-0.004	0.99525	0.985	0.00000	0.00000	pubmed
ENSMUSG00000035828	Pim3	391.92±73.66	754.85±36.66	311.38±22.19	750.1±50.8	0.946	0.00398	-0.332	0.49312	0.937	0.00250	0.00250	pubmed
ENSMUSG00000035934	Pknox2	10.98±2.26	29.28±4.56	14.72±0.58	36.08±8.95	1.413	0.00133	0.422	1.00000	1.718	0.00002	0.00002	pubmed
ENSMUSG00000036040	Adamtsl2	12.6±3.43	69.87±14.12	9.49±1.8	59.13±16.97	2.469	0.00000	-0.408	0.58750	2.228	0.00000	0.00000	pubmed
ENSMUSG00000036206	Sh3bp4	246.69±30.69	508.43±17.99	247.76±11.71	575.7±74.66	1.043	0.00000	0.006	0.99231	1.222	0.00000	0.00000	pubmed
ENSMUSG00000036478	Btg1	1820.7±462.37	4242.71±171.09	2078.04±243.93	4262.39±625.52	1.221	0.00000	0.191	0.59543	1.227	0.00000	0.00000	pubmed
ENSMUSG00000036672	Cenpt	65.75±1.59	115.93±13.94	57.14±3.76	96.89±7.08	0.818	0.00006	-0.203	0.56281	0.561	0.00851	0.00851	pubmed
ENSMUSG00000037336	Mfsd2b	12.66±5.02	32.45±2.85	19.22±2.07	44.67±8.22	1.359	0.00468	0.605	1.00000	1.818	0.00002	0.00002	pubmed
ENSMUSG00000037526	Atg14	159.13±21.81	279.46±34.36	152.66±14.94	235.77±18.24	0.813	0.00006	-0.060	0.89297	0.567	0.00648	0.00648	pubmed
ENSMUSG00000037685	Atp8a1	289.56±25.83	697.93±27.18	239.34±11.23	591.42±53.03	1.270	0.00000	-0.274	0.10475	1.032	0.00000	0.00000	pubmed
ENSMUSG00000037762	Slc16a9	19.25±4.09	38.66±8.26	13.33±2.54	32.27±0.86	1.008	0.00060	-0.523	0.26196	0.750	0.01291	0.01291	pubmed
ENSMUSG00000037797	Adh4	1.86±0.62	14.37±3.62	2.53±2.16	15.64±7.12	2.941	0.00002	0.417	0.79434	3.071	0.00000	0.00000	pubmed
ENSMUSG00000037904	Ankrd9	103.27±11.15	284.38±9.55	110.64±11.13	335.65±26.96	1.462	0.00000	0.099	0.79216	1.700	0.00000	0.00000	pubmed
ENSMUSG00000038068	Rnf144b	39.96±3.14	78.69±19.77	56.6±6.86	78.61±25.18	0.977	0.00226	0.500	0.23887	0.979	0.00117	0.00117	pubmed
ENSMUSG00000038301	Snx10	152.47±7.29	270.66±4.04	126.95±19.32	234.19±24.06	0.828	0.00001	-0.263	0.36870	0.620	0.00128	0.00128	pubmed
ENSMUSG00000038332	Sesn1	1012.65±17.24	4341.15±92.79	973.38±40.49	4033.74±762.42	2.100	0.00000	-0.057	0.87407	1.994	0.00000	0.00000	pubmed
ENSMUSG00000038342	Mlxip	773.12±120.7	1705.58±48.19	678.16±41.92	1800.4±31.94	1.142	0.00000	-0.189	0.39325	1.220	0.00000	0.00000	pubmed
ENSMUSG00000038393	Txnip	3349.86±275.58	12445.02±245.52	3948.15±180.49	18118.38±2891.69	1.893	0.00000	0.237	0.50575	2.435	0.00000	0.00000	pubmed
ENSMUSG00000038415	Foxq1	30.22±1.11	128.59±53.81	57.08±18.45	173.5±60.14	2.090	0.00020	0.917	0.23729	2.521	0.00000	0.00000	pubmed
ENSMUSG00000038526	Car14	10.01±3.29	28.47±0.63	7.27±0.98	44.7±9.58	1.503	0.00007	-0.470	0.51111	2.158	0.00000	0.00000	pubmed
ENSMUSG00000038534	Osbpl7	437.67±25.67	765.81±34.55	388.39±16.14	702.07±96.75	0.807	0.00000	-0.173	0.51164	0.681	0.00002	0.00002	pubmed
ENSMUSG00000038541	Srd5a2	9.14±2.13	48.43±0.7	5.45±0.64	29.28±0.7	2.407	0.00000	-0.747	1.00000	1.682	0.00000	0.00000	pubmed
ENSMUSG00000038756	Ttll6	2.77±1.99	14.1±0.86	7.89±3.12	32.82±11.8	2.336	0.00725	1.501	1.00000	3.553	0.00000	0.00000	pubmed
ENSMUSG00000038859	Baiap2l1	1464.44±111.12	2827.8±60.15	1178.83±28.53	2454.76±382.45	0.949	0.00000	-0.313	0.17661	0.745	0.00001	0.00001	pubmed
ENSMUSG00000039157	Fam102a	1222.4±270.94	4690.99±511.31	1253.05±271.52	4255.19±1323.03	1.940	0.00000	0.036	0.96438	1.799	0.00000	0.00000	pubmed
ENSMUSG00000039196	Orm1	7.33±4.57	37.17±18.05	14.27±10.03	13.74±12.89	2.346	0.01356	0.962	0.47661	0.919	0.40102	0.40102	pubmed
ENSMUSG00000039202	Abhd2	2776.32±117.78	5613.63±388.89	2777.85±118.44	5941.23±1391.7	1.016	0.00007	0.001	0.99995	1.098	0.00001	0.00001	pubmed
ENSMUSG00000039313	AF529169	1.17±0.32	14.62±4.26	2.98±1.64	46.06±8.14	3.644	0.00012	1.340	1.00000	5.300	0.00000	0.00000	pubmed
ENSMUSG00000039457	Ppl	59.48±10.86	110.67±3.37	95.63±10.47	148.33±7.53	0.895	0.01769	0.684	0.11381	1.317	0.00004	0.00004	pubmed
ENSMUSG00000039611	Tmem246	317.13±36	576.22±49.76	371.32±27.95	660.69±90.42	0.862	0.00000	0.228	0.41208	1.059	0.00000	0.00000	pubmed
ENSMUSG00000039883	Lrrc17	18.72±4.92	55.12±5.44	22.41±1.56	87.54±4.44	1.557	0.00000	0.260	1.00000	2.225	0.00000	0.00000	pubmed

Ensgene	Symbol	HP	HD	ZP	ZD	LFC HD-HP	FDR HD-HP	LFC ZP-HP	FDR ZP-HP	LFC ZD-HP	FDR ZD-HP	Ratio	Links
ENSMUSG00000039903	Eva1c	4±1.71	21.28±6.17	4.32±1.32	23.93±3.35	2.417	0.00002	0.113	1.00000	2.584	0.00000	0.00000	pubmed
ENSMUSG00000039958	Mettl20	143.9±11.81	317.47±11.66	163.53±11.48	279.64±18.27	1.141	0.00000	0.184	0.64667	0.958	0.00002	0.00002	pubmed
ENSMUSG00000039960	Rhou	613.83±60.4	2454.91±148.18	606.29±18.35	2031.01±522.65	2.000	0.00000	-0.018	0.97380	1.726	0.00000	0.00000	pubmed
ENSMUSG00000040111	Gramd1b	510.75±67.2	1046±111.25	480.34±15.89	1000.54±307.58	1.034	0.00009	-0.089	0.87241	0.970	0.00016	0.00016	pubmed
ENSMUSG00000040522	Tlr8	16.11±2.57	35.89±5.52	12.5±1.98	29.87±4.59	1.154	0.00241	-0.370	0.57093	0.893	0.01922	0.01922	pubmed
ENSMUSG00000040613	Apobec1	338.13±19.85	1060.77±55.74	328.97±24.45	800.55±177.29	1.649	0.00000	-0.040	0.94349	1.243	0.00000	0.00000	pubmed
ENSMUSG00000040808	S100g	62.27±15.05	139.8±35.13	82.35±22.8	284.21±99.54	1.166	0.01926	0.402	0.58422	2.190	0.00000	0.00000	pubmed
ENSMUSG00000040957	Cables1	259.27±17.18	1534.54±134.09	366.54±38.07	1506.82±292.83	2.565	0.00000	0.500	0.13396	2.539	0.00000	0.00000	pubmed
ENSMUSG00000041119	Pde9a	1619.74±246.75	3823.23±351.83	1424.36±287.69	3983.55±965.32	1.239	0.00000	-0.186	0.66846	1.298	0.00000	0.00000	pubmed
ENSMUSG00000041132	N4bp2l1	56.98±12.41	144.9±20.32	56.57±10.01	195.79±31.23	1.347	0.00000	-0.009	0.99237	1.781	0.00000	0.00000	pubmed
ENSMUSG00000041238	Rbbp8	201.73±12.8	678.74±45.27	221.65±13.44	783.27±92.43	1.750	0.00000	0.135	0.59224	1.957	0.00000	0.00000	pubmed
ENSMUSG00000041417	Pik3r1	931.13±90.3	1914.98±61.25	1023.19±44.4	2064.91±142.36	1.040	0.00000	0.136	0.59469	1.149	0.00000	0.00000	pubmed
ENSMUSG00000041471	Fam35a	39.12±2.63	68.92±8.82	41.82±4.92	72.03±2.44	0.818	0.00031	0.099	0.82981	0.882	0.00003	0.00003	pubmed
ENSMUSG00000041548	Hspb8	130.47±9.24	239.01±24.98	161.81±43.35	293.95±65.06	0.873	0.00024	0.310	0.37507	1.173	0.00000	0.00000	pubmed
ENSMUSG00000041642	Kif21b	655.86±104.53	1161.62±66.61	554.78±90.74	765.94±133.1	0.825	0.00007	-0.241	0.45229	0.224	0.38737	0.38737	pubmed
ENSMUSG00000041644	Slc5a12	154.3±27.15	730.68±164.99	220.96±40.95	1238.39±409.59	2.244	0.00000	0.518	0.30357	3.005	0.00000	0.00000	pubmed
ENSMUSG00000041831	Sytl3	62.31±4.22	136.13±15.58	60.45±8.46	102.77±9.59	1.128	0.00000	-0.045	0.94010	0.721	0.00280	0.00280	pubmed
ENSMUSG00000042116	Vwa1	144.45±11.59	375.1±53	247.77±23.4	590.48±131.16	1.377	0.00000	0.779	0.00519	2.031	0.00000	0.00000	pubmed
ENSMUSG00000042246	Tmc7	73.63±18.46	209.47±28.93	84.47±23.13	216.32±95.96	1.508	0.00022	0.198	0.80215	1.553	0.00007	0.00007	pubmed
ENSMUSG00000042472	Zfp410	270.55±26.07	581.59±41.6	272.88±15.16	524.04±83.3	1.104	0.00000	0.012	0.97957	0.953	0.00000	0.00000	pubmed
ENSMUSG00000042510	AA986860	1081.56±51.18	2122.85±101.71	1028.76±92.51	1747.6±460.21	0.973	0.00001	-0.072	0.87985	0.692	0.00253	0.00253	pubmed
ENSMUSG00000042540	Acot5	0.23±0.32	78.26±8.4	3.44±2.5	73.81±11.59	8.386	0.00000	3.865	1.00000	8.300	0.00000	0.00000	pubmed
ENSMUSG00000042622	Maff	118.51±9.21	377.55±35.41	125.32±5.25	361.97±64.67	1.672	0.00000	0.080	0.92621	1.610	0.00001	0.00001	pubmed
ENSMUSG00000042638	Gucy2c	2517.99±266.08	4599.04±273.99	2644.29±192.61	4802.85±773.58	0.869	0.00007	0.071	0.87716	0.932	0.00001	0.00001	pubmed
ENSMUSG00000042688	Mapk6	1536.77±156.76	4117.38±158.54	1761.63±139.98	4128.67±617.16	1.422	0.00000	0.197	0.39362	1.426	0.00000	0.00000	pubmed
ENSMUSG00000042807	Hecw2	22.2±1.72	42.54±4.2	22.2±0.73	49.57±8.52	0.937	0.00099	-0.001	0.99995	1.154	0.00001	0.00001	pubmed
ENSMUSG00000043487	Acot6	19.26±3.81	143.03±18.51	18.81±0.24	91.85±17.85	2.896	0.00000	-0.031	0.97544	2.255	0.00000	0.00000	pubmed
ENSMUSG00000043639	Rbm20	23.75±6.42	51.56±7.62	17.43±2.74	52.08±6.24	1.115	0.00085	-0.449	0.40117	1.129	0.00036	0.00036	pubmed
ENSMUSG00000044167	Foxo1	530.84±119.13	1060.4±95.48	493.57±72.88	820.39±257.15	0.998	0.00008	-0.105	0.83635	0.628	0.01756	0.01756	pubmed
ENSMUSG00000044244	Il20rb	9.14±1.11	20.77±1.81	13.67±3.84	21.89±5.13	1.184	0.04610	0.579	1.00000	1.264	0.01554	0.01554	pubmed
ENSMUSG00000044469	Tnfaip8l1	95.22±8.75	269.98±15.9	117.3±9.11	260.74±18.79	1.503	0.00000	0.300	0.23887	1.452	0.00000	0.00000	pubmed
ENSMUSG00000044583	Tlr7	47.38±25.68	120.19±17.31	35.46±9.53	112.95±7.18	1.345	0.00020	-0.415	0.47509	1.256	0.00036	0.00036	pubmed
ENSMUSG00000044636	Csrnp2	17.07±2.64	32.98±2.47	23.64±3.44	51.19±15.66	0.950	0.04966	0.468	1.00000	1.580	0.00004	0.00004	pubmed
ENSMUSG00000044715	Gskip	294.53±19.93	622.67±35.83	311.61±9.34	629.85±119.9	1.080	0.00000	0.081	0.83799	1.096	0.00000	0.00000	pubmed
ENSMUSG00000044748	Defb1	4.24±3.03	11.67±8.61	1.58±0.28	5.35±2.43	1.469	0.03442	-1.412	0.22065	0.326	0.73907	0.73907	pubmed
ENSMUSG00000045071	E130308A19Rik	51.31±2.82	92.84±4.2	64.86±11.22	125.14±11.04	0.857	0.00008	0.336	0.28308	1.286	0.00000	0.00000	pubmed
ENSMUSG00000045094	Arhgef37	20.43±8.92	57.96±11.12	29.69±0.16	69.92±3.77	1.498	0.00000	0.532	0.20378	1.768	0.00000	0.00000	pubmed
ENSMUSG00000045216	Hs6st1	668.42±39.58	1262.43±61.52	536.64±28.32	1055.98±140.05	0.917	0.00000	-0.317	0.06586	0.659	0.00000	0.00000	pubmed
ENSMUSG00000045534	Kcna5	7.54±2.69	29.24±3.1	13.64±3.08	55.56±7.86	1.962	0.00004	0.859	1.00000	2.889	0.00000	0.00000	pubmed
ENSMUSG00000045658	Pid1	55.98±4.95	99.48±8.08	77.71±16.65	122.76±18.14	0.830	0.00009	0.474	0.07568	1.136	0.00000	0.00000	pubmed
ENSMUSG00000045667	Smtnl2	19.61±2.8	56.05±17.9	18.87±2.66	81.4±8.94	1.514	0.00011	-0.059	1.00000	2.053	0.00000	0.00000	pubmed
ENSMUSG00000045730	Adrb2	23.45±11.14	103.45±21.26	19.38±5.49	85.27±11.93	2.142	0.00000	-0.272	0.75335	1.863	0.00001	0.00001	pubmed
ENSMUSG00000046318	Ccbe1	40.59±8.18	70.98±7.91	61.82±3.4	85.33±8.66	0.809	0.00050	0.610	0.02082	1.076	0.00000	0.00000	pubmed
ENSMUSG00000046546	Fam43a	72.42±40.27	174.85±50.17	65±23.04	147.44±16.18	1.272	0.00669	-0.154	0.86414	1.026	0.02486	0.02486	pubmed
ENSMUSG00000046694	Fam46b	17.74±2.57	53.2±3.31	23±8.33	63.84±6.91	1.583	0.00005	0.370	1.00000	1.847	0.00000	0.00000	pubmed
ENSMUSG00000046959	Slc26a1	1.17±0.32	5.83±5.41	2.25±0.79	4.46±1.42	2.322	0.01476	0.953	0.52406	1.945	0.03674	0.03674	pubmed
ENSMUSG00000047150	1700001C19Rik	0.94±0.89	4.51±1.85	1.6±1.8	7.7±1.62	2.272	0.04281	0.761	0.68486	3.050	0.00081	0.00081	pubmed
ENSMUSG00000047298	Kcnv2	0.46±0.64	33.8±5.2	3.19±0.7	44.37±13.43	6.177	0.00000	2.765	1.00000	6.567	0.00000	0.00000	pubmed
ENSMUSG00000047344	Lancl3	13.76±2.41	56.46±5.37	21.3±3.05	92.55±22.14	2.033	0.00000	0.628	0.25505	2.749	0.00000	0.00000	pubmed
ENSMUSG00000047787	Flrt1	2.79±1.49	10.64±0.98	1.6±0.88	15.61±4.17	1.925	0.01747	-0.820	1.00000	2.475	0.00036	0.00036	pubmed
ENSMUSG00000048489	8430408G22Rik	9.9±3.63	97.34±7.73	9.46±4.26	136.28±18.4	3.300	0.00000	-0.061	0.97759	3.785	0.00000	0.00000	pubmed
ENSMUSG00000048490	Nrip1	858.88±89.33	1723.07±42.92	1006.77±93.88	1776.74±332.15	1.005	0.00000	0.229	0.36651	1.048	0.00000	0.00000	pubmed
ENSMUSG00000048546	Tob2	381.54±4.24	671.4±57.51	389.8±23.77	753.6±132.82	0.815	0.00001	0.031	0.94725	0.981	0.00000	0.00000	pubmed
ENSMUSG00000048572	Tmem252	309.68±58.79	952.88±57.82	413.69±27.06	932.93±78.15	1.621	0.00000	0.418	0.07167	1.591	0.00000	0.00000	pubmed
ENSMUSG00000049115	Agtr1a	18.98±4.81	39.89±5.55	23.8±0.72	47.43±4.65	1.074	0.00001	0.328	0.41051	1.327	0.00000	0.00000	pubmed
ENSMUSG00000049307	Fut4	400.11±42.02	1005.8±60.54	323.74±12.91	769.84±88.91	1.330	0.00000	-0.306	0.14719	0.944	0.00000	0.00000	pubmed
ENSMUSG00000049409	Prokr1	4.47±1.51	15.16±2.19	7.72±0.46	16.54±5.94	1.767	0.00422	0.791	1.00000	1.901	0.00079	0.00079	pubmed
ENSMUSG00000049580	Tsku	59.67±4.08	151.46±45.67	62.15±21.71	125.56±2.25	1.344	0.00005	0.059	0.94252	1.073	0.00130	0.00130	pubmed
ENSMUSG00000049791	Fzd4	53.66±6.79	102.86±13.78	63.73±11.69	130.13±21.73	0.940	0.00014	0.247	0.53840	1.281	0.00000	0.00000	pubmed
ENSMUSG00000049892	Rasd1	16.95±4.82	255.44±15.06	17.82±6.21	185.13±62.87	3.917	0.00000	0.071	0.94994	3.451	0.00000	0.00000	pubmed
ENSMUSG00000050075	Gpr171	62.25±23.18	138.04±24.05	30.98±15.17	86.25±2.68	1.149	0.01730	-1.003	0.07282	0.471	0.38218	0.38218	pubmed
ENSMUSG00000050164	Mchr1	3.69±2.72	13.57±2.29	7.04±1.34	27.82±0.55	1.865	0.01261	0.917	1.00000	2.901	0.00000	0.00000	pubmed
ENSMUSG00000050628	Ubald2	663.06±98.11	1317.72±129.68	803.98±45.29	1258.78±119.2	0.991	0.00000	0.278	0.28277	0.925	0.00000	0.00000	pubmed
ENSMUSG00000050666	Vstm4	51.42±8.57	119.43±8.91	70.83±8.31	140.26±5.95	1.218	0.00000	0.465	0.07985	1.451	0.00000	0.00000	pubmed
ENSMUSG00000050761	Gp1bb	225.92±24.07	640.59±103.27	202.29±14.28	749.62±60.52	1.504	0.00000	-0.159	0.63570	1.730	0.00000	0.00000	pubmed
ENSMUSG00000050777	Tmem37	198.18±21.88	1320.39±78.95	206.28±17.33	1498.74±323.84	2.736	0.00000	0.058	0.89868	2.918	0.00000	0.00000	pubmed
ENSMUSG00000051000	Fam160a1	125.02±7.33	253.09±26.58	128.63±6.92	290.08±56.92	1.018	0.00001	0.041	0.94239	1.213	0.00000	0.00000	pubmed
ENSMUSG00000052131	Akr1b7	808.4±110.54	5648.04±610.3	693.32±224.91	4812.4±1165.19	2.805	0.00000	-0.222	0.67236	2.574	0.00000	0.00000	pubmed
ENSMUSG00000052253	Zfp622	374.96±41.99	674.15±26.46	496.74±32.79	774.16±90.61	0.846	0.00000	0.405	0.00840	1.045	0.00000	0.00000	pubmed
ENSMUSG00000053101	Gpr141	12.56±4.68	36.45±1.76	12.92±1.65	44.57±4.61	1.532	0.00016	0.036	1.00000	1.822	0.00000	0.00000	pubmed
ENSMUSG00000053198	Prx	27.15±5.49	63.56±6.77	29.18±2.68	53.39±13.18	1.227	0.00059	0.105	1.00000	0.972	0.00678	0.00678	pubmed

Ensgene	Symbol	HP	HD	ZP	ZD	LFC HD-HP	FDR HD-HP	LFC ZP-HP	FDR ZP-HP	LFC ZD-HP	FDR ZD-HP	Ratio	Links
ENSMUSG00000054150	Syne3	47.01±10.82	222.19±4	41.77±8.76	181.77±30.39	2.243	0.00000	-0.166	0.73900	1.953	0.00000	0.00000	pubmed
ENSMUSG00000054256	Msi1	53.75±7.75	172.45±21.02	53.93±5.18	232.81±36.33	1.681	0.00000	0.004	0.99832	2.114	0.00000	0.00000	pubmed
ENSMUSG00000054277	Arfgap3	1006.63±30.73	2019±142.18	1216.73±66.73	2190.79±384.26	1.004	0.00022	0.273	0.52107	1.122	0.00001	0.00001	pubmed
ENSMUSG00000055044	Pdlim1	1833.33±147.42	3445.56±227.38	2140.25±225.91	3901.79±257.29	0.910	0.00000	0.223	0.21390	1.089	0.00000	0.00000	pubmed
ENSMUSG00000055782	Abcd2	21.15±5.42	50.87±17.9	30.22±16.29	25.78±12.43	1.267	0.03582	0.514	0.54361	0.293	0.70590	0.70590	pubmed
ENSMUSG00000055980	Irs1	23.83±3.26	50.81±2.8	40.66±9.67	79.99±3.78	1.091	0.00043	0.768	0.03453	1.746	0.00000	0.00000	pubmed
ENSMUSG00000056131	Pgm3	391.49±37.43	787.6±9.93	410.99±26.77	868.91±11.02	1.009	0.00000	0.070	0.84145	1.150	0.00000	0.00000	pubmed
ENSMUSG00000056313	1810011O10Rik	736.8±142.46	1928.4±237.32	782.89±59.88	2137.74±336.52	1.388	0.00000	0.088	0.85847	1.537	0.00000	0.00000	pubmed
ENSMUSG00000056749	Nfil3	284.71±79.08	532.52±25.7	195.55±11.58	396.68±25.03	0.904	0.00359	-0.542	0.16631	0.479	0.14878	0.14878	pubmed
ENSMUSG00000056973	Ces1d	174.05±29.16	310.02±84.96	280.59±80.21	286.46±51.3	0.833	0.00318	0.689	0.02778	0.719	0.00864	0.00864	pubmed
ENSMUSG00000057329	Bcl2	143.19±39.09	255.77±30.46	162.17±27.45	371.62±25.93	0.837	0.00192	0.180	0.68965	1.376	0.00000	0.00000	pubmed
ENSMUSG00000058207	Serpina3k	13.3±4.94	85.03±114.61	4.5±2.78	51.31±56.68	2.676	0.04030	-1.562	0.36934	1.949	0.12161	0.12161	pubmed
ENSMUSG00000058503	Fam133b	107.48±12.43	188.67±7.61	114.49±10.88	262.43±35.46	0.812	0.00000	0.091	0.79216	1.287	0.00000	0.00000	pubmed
ENSMUSG00000058761	Rnf169	156.88±18.64	717.94±28.52	158.16±3.17	676.75±80.44	2.195	0.00000	0.013	0.98156	2.109	0.00000	0.00000	pubmed
ENSMUSG00000059401	Mamld1	6.32±0.6	19.44±1.19	14.02±2.59	25.91±3.94	1.620	0.00029	1.152	1.00000	2.040	0.00000	0.00000	pubmed
ENSMUSG00000059811	Atl2	1147.86±80.35	2334.47±235.82	1104.19±91.42	2117.42±381.79	1.024	0.00000	-0.056	0.86311	0.883	0.00000	0.00000	pubmed
ENSMUSG00000059901	Adamts14	15.19±1.34	32.16±5.01	16.07±3.31	48.44±3.63	1.082	0.00335	0.081	1.00000	1.673	0.00000	0.00000	pubmed
ENSMUSG00000060613	Cyp2c70	0.93±0.66	10.04±12.51	0.69±0.56	8.74±6.76	3.428	0.01769	-0.451	0.89604	3.233	0.01645	0.01645	pubmed
ENSMUSG00000060716	Plekhh1	583.66±46	2041.07±60.86	699.92±42.07	2717.05±343.74	1.806	0.00000	0.262	0.21313	2.219	0.00000	0.00000	pubmed
ENSMUSG00000061175	Fnip2	85.92±2.09	153.37±21.37	95.17±1.27	213.74±33.89	0.836	0.00019	0.148	0.71393	1.314	0.00000	0.00000	pubmed
ENSMUSG00000061740	Cyp2d22	91.93±20.11	174.1±20.34	120.12±22.3	172.38±35.11	0.920	0.00002	0.385	0.19354	0.904	0.00001	0.00001	pubmed
ENSMUSG00000061808	Ttr	31.1±5.44	70.99±51.9	44.2±2.9	130.52±33.52	1.191	0.03445	0.507	0.51410	2.070	0.00001	0.00001	pubmed
ENSMUSG00000062232	Rapgef2	235±31.01	761.89±56.14	220.6±27.07	690.41±183.55	1.697	0.00000	-0.092	0.84877	1.554	0.00000	0.00000	pubmed
ENSMUSG00000063522	2010109I03Rik	1398.9±512.48	4611.85±592.62	2178.2±467.85	6138.31±3329.35	1.721	0.01527	0.639	0.52766	2.134	0.00073	0.00073	pubmed
ENSMUSG00000064036	Mro	49.99±9.13	94.51±11.91	38.72±2.34	65.22±17.13	0.920	0.01015	-0.367	0.48686	0.383	0.34226	0.34226	pubmed
ENSMUSG00000064120	Mocs1	342.95±28.38	658.88±95.64	327.9±29.5	564.44±36.47	0.943	0.00000	-0.065	0.82780	0.719	0.00000	0.00000	pubmed
ENSMUSG00000066170	E230001N04Rik	6.08±0.53	71.87±21.7	12.73±1.54	72.47±11.01	3.563	0.00000	1.063	1.00000	3.574	0.00000	0.00000	pubmed
ENSMUSG00000066361	Serpina3c	3.99±0.7	22.08±9.04	8.04±4.87	14.77±6.14	2.471	0.00044	1.007	1.00000	1.898	0.00828	0.00828	pubmed
ENSMUSG00000066515	Klk1b3	0.24±0.34	10.89±5.23	0±0	54.51±27.04	5.538	0.00550	-15.085	1.00000	7.861	0.00001	0.00001	pubmed
ENSMUSG00000066687	Zbtb16	57.38±32.04	402.87±85.4	79.24±37.09	454.86±54.12	2.812	0.00005	0.466	0.70620	2.988	0.00001	0.00001	pubmed
ENSMUSG00000067199	Frat1	18.8±5.06	42.8±4.69	16.34±3.49	36.99±6.12	1.189	0.02821	-0.201	1.00000	0.977	0.05790	0.05790	pubmed
ENSMUSG00000067780	Pi15	15.67±0.6	60.14±5.23	18.82±7.26	63.92±19.06	1.940	0.00002	0.264	1.00000	2.030	0.00000	0.00000	pubmed
ENSMUSG00000067813	Xkr9	224.03±25.2	492.08±43.84	189.67±10.41	459.83±91.73	1.135	0.00000	-0.240	0.40726	1.037	0.00000	0.00000	pubmed
ENSMUSG00000068600	Gml2	11.01±1.84	50.02±13.89	2.97±0.91	8.46±4.98	2.184	0.00175	-1.898	1.00000	-0.391	0.70816	0.70816	pubmed
ENSMUSG00000068742	Cry2	101.14±13.08	435.35±55.5	142.9±16.3	469.52±125.03	2.107	0.00000	0.499	0.11069	2.215	0.00000	0.00000	pubmed
ENSMUSG00000070576	Mn1	22.88±5.08	71.29±5.05	34.37±15.61	83.96±3.1	1.638	0.00000	0.583	0.18401	1.874	0.00000	0.00000	pubmed
ENSMUSG00000070643	Sox13	824.63±14.68	1851.34±171.75	854.17±74.65	1888.92±395.27	1.167	0.00000	0.051	0.91645	1.196	0.00000	0.00000	pubmed
ENSMUSG00000071347	C1qtnf9	18.04±5.56	45.44±7.53	19.57±3.61	49.88±6.3	1.334	0.00130	0.116	1.00000	1.469	0.00013	0.00013	pubmed
ENSMUSG00000071379	Hpcal1	334.98±27.44	584.96±23.09	437.01±35.07	692.28±54.71	0.805	0.00000	0.384	0.01923	1.047	0.00000	0.00000	pubmed
ENSMUSG00000071506	Tmem139	105.59±7.59	187.17±26.56	130.09±9.75	165.74±38.37	0.826	0.00340	0.301	0.46420	0.649	0.01959	0.01959	pubmed
ENSMUSG00000072214	Sept5	2831.45±196.14	5422.54±470.37	2643.55±246.57	5042.91±550.95	0.937	0.00000	-0.099	0.72904	0.833	0.00000	0.00000	pubmed
ENSMUSG00000072872	Rybp	468.24±6.97	849.6±37.7	463.16±6.05	858.01±182.01	0.860	0.00000	-0.016	0.97361	0.873	0.00000	0.00000	pubmed
ENSMUSG00000074207	Adh1	156.18±5.11	670.88±32.45	181.15±23.3	519.91±80.34	2.103	0.00000	0.215	0.34972	1.734	0.00000	0.00000	pubmed
ENSMUSG00000074259	Gramd2	38.07±11.22	90.3±22.17	29.98±6.52	113.14±10.67	1.248	0.00150	-0.344	0.59854	1.572	0.00001	0.00001	pubmed
ENSMUSG00000074622	Mafb	465.85±73.93	1086.47±147.89	307.96±17.42	1052.49±336.55	1.222	0.00004	-0.597	0.12652	1.176	0.00005	0.00005	pubmed
ENSMUSG00000074623	Gm826	2.55±1.96	10.14±4.39	1.36±0.03	11.5±3.36	1.979	0.00995	-0.917	1.00000	2.164	0.00178	0.00178	pubmed
ENSMUSG00000074715	Ccl28	930.2±133.86	2221.82±209.45	676.78±40.21	1782.52±144.5	1.256	0.00000	-0.459	0.02131	0.938	0.00000	0.00000	pubmed
ENSMUSG00000074794	Arrdc3	355.48±14.77	1310.86±27.16	398.35±46.34	1622.67±63.06	1.883	0.00000	0.164	0.64216	2.190	0.00000	0.00000	pubmed
ENSMUSG00000074874	Ctla2b	21.56±2.87	38.01±4.91	15.65±3.53	31.05±6.14	0.820	0.00848	-0.462	0.31737	0.528	0.10271	0.10271	pubmed
ENSMUSG00000075511	1700001L05Rik	24.59±4.43	54.1±8.22	23.6±6.33	75.06±8.91	1.137	0.00105	-0.060	0.94464	1.612	0.00000	0.00000	pubmed
ENSMUSG00000076431	Sox4	294.9±31.27	785.75±76.92	338.73±5.08	1001.22±152.01	1.414	0.00000	0.200	0.62730	1.763	0.00000	0.00000	pubmed
ENSMUSG00000078234	Klhdc7a	18.17±7.53	62.31±21.14	27.21±12.13	33.85±30.48	1.780	0.04366	0.583	0.65432	0.903	0.32683	0.32683	pubmed
ENSMUSG00000078612	1700024P16Rik	94.82±24.9	497.96±55.91	135.98±58.01	643.06±201.05	2.392	0.00000	0.519	0.36357	2.761	0.00000	0.00000	pubmed
ENSMUSG00000079012	Serpina3m	1.85±1.14	9.55±6.21	0.23±0.33	4.87±3.61	2.355	0.00070	-3.046	0.21724	1.418	0.07180	0.07180	pubmed
ENSMUSG00000079174	Gm3054	53.44±9.71	134.98±23.47	49.82±11.49	83.97±19.78	1.336	0.00020	-0.103	0.89604	0.649	0.10375	0.10375	pubmed
ENSMUSG00000079419	Ms4a6c	116.08±12.63	212.79±34.48	96.71±17.63	224.76±34.21	0.874	0.00007	-0.263	0.44929	0.954	0.00000	0.00000	pubmed
ENSMUSG00000079481	Nhsl2	40.15±7.81	76.32±5.44	46.29±7.55	94.93±15.98	0.925	0.00212	0.203	0.69095	1.242	0.00000	0.00000	pubmed
ENSMUSG00000079737	3110001I22Rik	27.92±3.45	60.68±4.52	26.94±2.34	52.2±8.9	1.121	0.00021	-0.049	0.94994	0.901	0.00311	0.00311	pubmed
ENSMUSG00000083813	Gm15502	3.49±0.91	23.67±9.69	3.84±1.75	16.56±7.18	2.757	0.00014	0.135	1.00000	2.235	0.00223	0.00223	pubmed
ENSMUSG00000084960	B430010I23Rik	10.8±1.84	56.93±13.24	15.68±2.46	31.99±5.06	2.400	0.00000	0.539	1.00000	1.567	0.00043	0.00043	pubmed
ENSMUSG00000085184	4933439K11Rik	11.2±2.1	46.84±0.72	8.17±0.64	53.05±1.38	2.061	0.00000	-0.460	1.00000	2.241	0.00000	0.00000	pubmed
ENSMUSG00000085241	Snhg3	50.03±6.23	96.06±3.5	48.43±6.32	107.3±18.64	0.940	0.00001	-0.047	0.93005	1.097	0.00000	0.00000	pubmed
ENSMUSG00000086389	Gm15998	4.42±1.35	15.65±5.42	11.72±4.36	9.21±4.72	1.820	0.00387	1.404	1.00000	1.037	0.13540	0.13540	pubmed
ENSMUSG00000086765	Gm11827	0.69±0.56	20.21±3.64	0.45±0.32	19.43±3.53	4.848	0.00000	-0.631	1.00000	4.786	0.00000	0.00000	pubmed
ENSMUSG00000087385	AA415398	12.44±2.21	21.83±0.89	16.37±2.35	19.09±1.45	0.814	0.04579	0.396	0.46873	0.618	0.11547	0.11547	pubmed
ENSMUSG00000089712	Gm15889	6.28±1.92	52.16±16.91	7.03±1.18	65.62±14.03	3.048	0.00000	0.156	0.89782	3.379	0.00000	0.00000	pubmed
ENSMUSG00000090264	Eif4ebp3	4.43±0.78	73.4±9.14	7.49±2.47	66.66±12.41	4.046	0.00000	0.752	0.36218	3.905	0.00000	0.00000	pubmed
ENSMUSG00000090698	Apold1	25.95±6.14	50.3±1.66	24±7.51	68.31±15.76	0.954	0.00976	-0.113	0.87931	1.393	0.00001	0.00001	pubmed
ENSMUSG00000091144	Phf11c	12.12±2.93	27.11±4.91	13.32±2.85	32.28±2.71	1.158	0.00032	0.136	0.84565	1.409	0.00000	0.00000	pubmed
ENSMUSG00000092008	Cyp2c69	0.92±0.85	10.56±13.83	0.22±0.31	3.85±3.94	3.509	0.04759	-2.043	0.58133	2.063	0.25861	0.25861	pubmed
ENSMUSG00000094091	Gm21885	101.42±7.7	246.05±24.67	165.82±9.13	356.85±71.98	1.279	0.00000	0.709	0.00585	1.815	0.00000	0.00000	pubmed

Ensgene	Symbol	HP	HD	ZP	ZD	LFC HD-HP	FDR HD-HP	LFC ZP-HP	FDR ZP-HP	LFC ZD-HP	FDR ZD-HP	Ratio	Links
ENSMUSG00000094806	Cyp2d10	3.49±1.08	12.47±5.82	3.14±1.61	19.16±4.92	1.833	0.00050	-0.146	0.91950	2.440	0.00000	0.00000	pubmed
ENSMUSG00000097203	4732419C18Rik	2.33±0.82	14.91±2.13	1.14±0.65	8.3±0.95	2.671	0.00001	-1.045	0.48099	1.822	0.00617	0.00617	pubmed
ENSMUSG00000097440	Gm6277	6.07±1.82	27.44±2.99	7.97±1.86	28.36±2.26	2.173	0.00001	0.386	1.00000	2.221	0.00000	0.00000	pubmed
ENSMUSG00000097615	Gm2061	147.54±12.27	347.41±46.89	114.07±33.42	234.8±94.87	1.236	0.00355	-0.371	0.57105	0.670	0.13733	0.13733	pubmed
ENSMUSG00000097908	4933404O12Rik	54.29±3.93	124.39±22.91	40.97±7.62	74.45±20.47	1.196	0.00008	-0.406	0.39504	0.452	0.20799	0.20799	pubmed
ENSMUSG00000098708	Gm27252	0.7±0.01	9.83±3.15	0.22±0.31	6.03±1.13	3.809	0.00069	-1.632	1.00000	3.099	0.00636	0.00636	pubmed
ENSMUSG00000100975	Gm28875	9.6±0.31	25.01±0.51	5.65±1.67	33.19±3.8	1.382	0.00203	-0.761	1.00000	1.788	0.00001	0.00001	pubmed
ENSMUSG00000101389	Ms4a4a	50.35±1.31	121.75±16.35	50.07±4.36	119.68±8.51	1.275	0.00000	-0.007	0.99361	1.250	0.00000	0.00000	pubmed
ENSMUSG00000103278	Gm37919	5.65±3.07	35.62±5.16	5.28±5.18	92.8±53.28	2.659	0.00025	-0.102	0.95987	4.040	0.00000	0.00000	pubmed
ENSMUSG00000103502	9330121J05Rik	12.83±2.34	30.62±2.22	15.3±4.8	29.92±5.88	1.251	0.00129	0.244	0.73465	1.216	0.00101	0.00101	pubmed
ENSMUSG00000104011	Gm32391	8.47±3.69	20.72±6.63	12.93±2.04	24.94±2.74	1.296	0.00836	0.614	1.00000	1.565	0.00031	0.00031	pubmed
ENSMUSG00000104340	Gm10522	34.4±8.87	81.92±4.82	38.21±7	42.05±17.37	1.252	0.01281	0.151	1.00000	0.286	0.66280	0.66280	pubmed

Appendix Table S7

List of genes derived from the RNASEQ experiment shown in Fig. 2. Definitions of the categories see Fig. 2.

Ensgene	Symbol	HP	HD	ZP	ZD	LFC HD-HP	FDR HD-HP	LFC ZP-HP	FDR ZP-HP	LFC ZD-HP	FDR ZD-HP	Ratio	Links
ENSMUSG00000030935	Acsm3	137.69±20.97	231.41±11.39	277.96±32.81	468.88±24.46	0.748	0.00000	1.011	0.00000	1.767	0.00000	0.00000	pubmed
ENSMUSG00000049122	Frmd3	41.15±2.31	62.5±4.84	91.7±7.84	171.11±11.29	0.603	0.02559	1.154	0.00000	2.056	0.00000	0.00000	pubmed

Appendix Table S8

List of genes derived from the RNASEQ experiment shown in Fig. 2. Definitions of the categories see Fig. 2.

Ensgene	Symbol	HP	HD	ZP	ZD	LFC HD-HP	FDR HD-HP	LFC ZP-HP	FDR ZP-HP	LFC ZD-HP	FDR ZD-HP	Ratio	Links
ENSMUSG00000000440	Pparg	59.49±4.68	69.22±7.77	127.59±21.47	134.95±58.23	0.218	0.72623	1.100	0.00247	1.181	0.00028	0.00028	pubmed
ENSMUSG00000000531	Grasp	20.59±1.48	24.44±4.73	38.1±3.33	32.09±3.63	0.248	0.69912	0.888	0.02568	0.639	0.09592	0.09592	pubmed
ENSMUSG00000001025	S100a6	871.41±127.29	981.35±55.49	1811.84±466.65	1924.66±427.09	0.171	0.75204	1.056	0.00056	1.143	0.00004	0.00004	pubmed
ENSMUSG00000002565	Scin	2177.19±488.72	1538.73±83.41	3954.4±304.56	3193.65±347.63	-0.501	0.03208	0.861	0.00003	0.553	0.00789	0.00789	pubmed
ENSMUSG00000003352	Cacnb3	261.48±13.5	232.54±4.89	474.51±62.62	407.46±50.83	-0.169	0.58830	0.859	0.00000	0.641	0.00041	0.00041	pubmed
ENSMUSG00000003555	Cyp17a1	1.38±1.47	2.12±1.34	5.9±1.44	6.17±2.25	0.603	0.74000	2.073	0.04003	2.153	0.01399	0.01399	pubmed
ENSMUSG00000004038	Gstm3	57.25±13	83.58±30.5	196.5±79.41	174.06±87.6	0.546	0.51449	1.779	0.00179	1.604	0.00235	0.00235	pubmed
ENSMUSG00000005125	Ndrg1	614.01±98.02	844±56.65	1325.68±154.9	1800.61±158.36	0.459	0.01224	1.111	0.00000	1.553	0.00000	0.00000	pubmed
ENSMUSG00000009614	Sardh	35.99±5.36	48.35±8.57	68.98±3.12	69±12.96	0.426	0.20681	0.937	0.00019	0.934	0.00007	0.00007	pubmed
ENSMUSG00000013523	Bcas1	424.61±15.61	472.05±61.19	753.76±90.59	827.85±91.9	0.153	0.62202	0.828	0.00001	0.963	0.00000	0.00000	pubmed
ENSMUSG00000018102	Hist1h2bc	546.93±62.53	612.5±44.08	959.36±84.66	900.86±120	0.163	0.57092	0.810	0.00000	0.719	0.00002	0.00002	pubmed
ENSMUSG00000018217	Pmp22	4492.32±1095.32	4376.78±158.99	8792.19±1069.09	5976.78±2437.45	-0.038	0.96806	0.969	0.00333	0.412	0.24069	0.24069	pubmed
ENSMUSG00000019232	Etnppl	13.52±6.04	20.95±10.9	40.44±19.61	37.05±1.38	0.631	0.30850	1.575	0.00030	1.451	0.00042	0.00042	pubmed
ENSMUSG00000020019	Ntn4	149.68±5.87	169.71±14.69	294.49±7.35	257.12±3.39	0.182	0.31890	0.977	0.00000	0.781	0.00000	0.00000	pubmed
ENSMUSG00000021136	Smoc1	45.03±10.13	73.46±17.27	81.4±12.75	81.04±8.93	0.707	0.00355	0.854	0.00026	0.852	0.00009	0.00009	pubmed
ENSMUSG00000021213	Akr1c13	376.04±48.86	387.81±34.06	778.61±139.85	668.1±96.11	0.044	0.92315	1.050	0.00000	0.829	0.00000	0.00000	pubmed
ENSMUSG00000021379	Id4	76.7±15.89	56.24±8.99	150.15±36.74	103.78±12.21	-0.449	0.42366	0.968	0.02086	0.436	0.32216	0.32216	pubmed
ENSMUSG00000021557	Agtpbp1	97.31±14.46	119.51±4.28	203.19±63.06	255.06±44.21	0.297	0.49811	1.062	0.00021	1.391	0.00000	0.00000	pubmed
ENSMUSG00000022098	Bmp1	155.72±9.08	157.96±14.15	273.12±20.41	198.9±12.86	0.020	0.95964	0.811	0.00000	0.351	0.00967	0.00967	pubmed
ENSMUSG00000022445	Cyp2d26	363.68±123.34	315.07±37.02	967.9±159.74	767.11±204.24	-0.207	0.68207	1.412	0.00000	1.076	0.00009	0.00009	pubmed
ENSMUSG00000022537	Tmem44	24.91±6.69	23.95±2.27	49.65±3.29	38.52±2.65	-0.060	0.95198	0.991	0.00745	0.626	0.09249	0.09249	pubmed
ENSMUSG00000022822	Abcc5	103.49±12.84	99.52±5.42	183.73±25	164.64±4.18	-0.057	0.90129	0.828	0.00002	0.669	0.00045	0.00045	pubmed
ENSMUSG00000023092	Fhl1	695.29±110.99	833.55±58.98	1243.24±147.13	1299.9±101.03	0.261	0.29371	0.838	0.00000	0.902	0.00000	0.00000	pubmed
ENSMUSG00000025194	Abcc2	437.91±90.26	551.72±156.26	885.63±369.24	542.96±165.07	0.333	0.57028	1.016	0.01302	0.310	0.50820	0.50820	pubmed
ENSMUSG00000025197	Cyp2c44	2.58±1.78	4.5±2.26	9.37±4.56	5.64±3.07	0.812	0.43586	1.854	0.00727	1.157	0.11439	0.11439	pubmed
ENSMUSG00000025474	Tubgcp2	123.64±14.56	106.44±7.26	290.9±76.79	392.16±41.54	-0.215	0.62171	1.234	0.00000	1.666	0.00000	0.00000	pubmed
ENSMUSG00000025515	Muc2	8061.56±171.28	7882.68±230.88	15125.58±2949.21	14132.77±3453.65	-0.032	0.96554	0.908	0.00030	0.810	0.00058	0.00058	pubmed
ENSMUSG00000026278	Bok	98.54±12.68	123.39±10.2	229.46±17.24	200.16±37.86	0.325	0.30939	1.219	0.00000	1.021	0.00000	0.00000	pubmed
ENSMUSG00000026433	Rab29	75.35±1.07	70.78±2.16	172.39±41.49	165.89±17.84	-0.090	0.85900	1.193	0.00000	1.140	0.00000	0.00000	pubmed
ENSMUSG00000026489	Adck3	175.81±13.56	217.45±25.84	435.94±46.01	391.17±64.51	0.307	0.46346	1.310	0.00000	1.154	0.00001	0.00001	pubmed
ENSMUSG00000027187	Cat	1228.72±29.64	1350.54±35.15	2205.47±193.78	1930.24±210.59	0.136	0.43825	0.844	0.00000	0.651	0.00000	0.00000	pubmed
ENSMUSG00000027495	Fam210b	238.95±13.91	273.63±20.96	448.59±60.5	498.68±56.59	0.195	0.42692	0.908	0.00000	1.061	0.00000	0.00000	pubmed
ENSMUSG00000027761	Aadac	14.06±1.14	14.87±7.44	66.72±11.42	69±14.07	0.085	0.92177	2.249	0.00000	2.300	0.00000	0.00000	pubmed
ENSMUSG00000027792	Bche	451.44±28.64	389.48±53.35	849.14±152.76	690.93±110.32	-0.213	0.49087	0.911	0.00000	0.614	0.00171	0.00171	pubmed
ENSMUSG00000027870	Hao2	38.5±10.58	31.09±7.83	92.89±10.22	83.38±12.03	-0.309	0.69085	1.270	0.00653	1.114	0.00962	0.00962	pubmed
ENSMUSG00000027876	Reg4	585.74±149.44	636.36±91.95	2075.33±651.98	1995.09±366.19	0.119	0.87142	1.825	0.00000	1.768	0.00000	0.00000	pubmed
ENSMUSG00000028003	Lrat	134.3±17.44	139.48±5.62	298.58±38.28	191.81±11.43	0.054	0.92003	1.152	0.00000	0.513	0.02293	0.02293	pubmed
ENSMUSG00000028088	Fmo5	1088.31±158.87	1411.05±141.41	2002.69±151.6	2020.31±476.85	0.375	0.12843	0.880	0.00001	0.892	0.00000	0.00000	pubmed
ENSMUSG00000028124	Gclm	785.47±45.54	1082.46±50.55	1418.08±183.96	1611.18±295.76	0.463	0.00730	0.852	0.00000	1.036	0.00000	0.00000	pubmed
ENSMUSG00000028755	Cda	85.36±12.83	60.41±4.27	280.88±50.74	193.54±34.74	-0.498	0.12547	1.719	0.00000	1.180	0.00000	0.00000	pubmed
ENSMUSG00000028780	Sema3c	225.32±16.49	332.41±4.73	435.16±73.91	559.65±66.16	0.561	0.01015	0.949	0.00000	1.312	0.00000	0.00000	pubmed
ENSMUSG00000028836	Slc30a2	416.98±79.93	533.55±32.32	1311.13±207.12	1639.66±237.68	0.356	0.26697	1.653	0.00000	1.975	0.00000	0.00000	pubmed
ENSMUSG00000029304	Spp1	4.01±2.23	5.85±0.98	11.38±4.63	10.65±4.15	0.555	0.57333	1.510	0.01420	1.406	0.01311	0.01311	pubmed
ENSMUSG00000029490	Mfsd7a	52.09±12.21	57.46±2.3	96.56±16.31	94.04±12.46	0.141	0.81547	0.890	0.00622	0.850	0.00404	0.00404	pubmed
ENSMUSG00000030110	Ret	125.5±5.79	127.87±16	307.55±71.23	354.95±13.57	0.027	0.97439	1.293	0.00000	1.500	0.00000	0.00000	pubmed
ENSMUSG00000030155	Clec2e	372.27±76.21	631.74±108.8	805.73±154.05	1104.86±281.44	0.763	0.03548	1.114	0.00092	1.569	0.00000	0.00000	pubmed
ENSMUSG00000030621	Me3	58.31±11.91	80.14±11.81	121.87±7.39	151.77±20.62	0.457	0.22881	1.062	0.00020	1.378	0.00000	0.00000	pubmed
ENSMUSG00000030834	Abcc6	37.56±6.29	35.4±7.53	72.72±3.92	69.27±3.4	-0.089	0.84800	0.950	0.00000	0.881	0.00000	0.00000	pubmed
ENSMUSG00000030905	Crym	34.91±5.22	31.16±3.87	86.39±11.37	79.12±11.74	-0.164	0.80346	1.307	0.00002	1.179	0.00007	0.00007	pubmed
ENSMUSG00000030935	Acsm3	137.69±20.97	231.41±11.39	277.96±32.81	468.88±24.46	0.748	0.00000	1.011	0.00000	1.767	0.00000	0.00000	pubmed
ENSMUSG00000031214	Ophn1	58.61±7.79	60.14±3.84	105.18±11.25	106.66±14.74	0.036	0.96199	0.842	0.00098	0.862	0.00022	0.00022	pubmed
ENSMUSG00000031762	Mt2	220.29±91.05	361.9±45.43	9444.61±2696.81	12205.88±1386.23	0.717	0.23943	5.422	0.00000	5.792	0.00000	0.00000	pubmed
ENSMUSG00000031790	Mmp15	306.78±10.88	294.02±26.35	537.84±22.41	437.26±29.36	-0.061	0.83254	0.810	0.00000	0.510	0.00014	0.00014	pubmed
ENSMUSG00000032348	Gsta4	141.21±33.14	178.62±38.17	333.84±32.32	427.51±63.33	0.338	0.31746	1.240	0.00000	1.598	0.00000	0.00000	pubmed
ENSMUSG00000032377	Plscr4	53.44±8.17	77.97±3.5	93.71±5.69	116.16±11.1	0.543	0.02392	0.809	0.00016	1.116	0.00000	0.00000	pubmed
ENSMUSG00000032788	Pdxk	403.82±21.77	314.11±22.16	762.08±66.43	553.18±30.57	-0.362	0.04366	0.916	0.00000	0.454	0.00265	0.00265	pubmed
ENSMUSG00000033720	Sfxn5	10.78±3.24	17.31±1.14	19.94±4.16	16.28±6.66	0.684	0.13382	0.889	0.02780	0.614	0.12840	0.12840	pubmed
ENSMUSG00000033721	Vav3	52.85±11.69	57.18±6.32	100.62±24.59	96.45±11.62	0.115	0.86044	0.931	0.00415	0.869	0.00356	0.00356	pubmed
ENSMUSG00000034353	Ramp1	116.37±21.61	147.88±8.46	213.35±26.1	270.17±18.16	0.344	0.18313	0.872	0.00001	1.214	0.00000	0.00000	pubmed
ENSMUSG00000034528	Hsd17b13	66.68±6.68	68.54±14.39	136.94±33.06	158.79±6.83	0.041	0.94468	1.037	0.00000	1.251	0.00000	0.00000	pubmed
ENSMUSG00000034687	Fras1	24.16±2.61	22.67±5.77	51.9±1.79	65.62±7.38	-0.092	0.91148	1.105	0.00112	1.442	0.00000	0.00000	pubmed
ENSMUSG00000034993	Vat1	858.07±68.9	745.44±49.78	1568.99±139.87	1027.67±143.21	-0.203	0.33428	0.870	0.00000	0.260	0.11128	0.11128	pubmed
ENSMUSG00000035112	Wnk4	34.14±2.04	33.52±3.94	73.93±1.47	93.94±7.05	-0.027	0.97690	1.114	0.00001	1.458	0.00000	0.00000	pubmed
ENSMUSG00000036473	Tbc1d24	76.64±13.04	55.04±8.82	138.17±9.6	133.94±19.41	-0.478	0.10897	0.849	0.00021	0.807	0.00018	0.00018	pubmed
ENSMUSG00000036814	Slc6a20a	830.83±46.33	707.79±100.41	1838.02±41.29	1118.41±217.58	-0.231	0.46392	1.145	0.00000	0.428	0.05135	0.05135	pubmed
ENSMUSG00000037949	Ano10	471.28±41.86	522.7±42.24	833.07±72.9	701.14±120	0.149	0.59663	0.822	0.00000	0.572	0.00057	0.00057	pubmed
ENSMUSG00000039063	Echdc3	8.44±1.61	13.56±3.38	16.79±0.95	18.13±3.82	0.687	0.17414	0.993	0.01876	1.116	0.00243	0.00243	pubmed
ENSMUSG00000040147	Maob	170.22±34	182.09±10.67	491.25±65.31	426.57±14.27	0.095	0.75716	1.527	0.00000	1.323	0.00000	0.00000	pubmed
ENSMUSG00000040584	Abcb1a	3358.22±520.93	3279.07±349.92	8097.03±1729.38	5290.69±2474.97	-0.034	0.97499	1.270	0.00022	0.656	0.06648	0.06648	pubmed
ENSMUSG00000041012	Cmtm8	2.56±0.84	5.32±1.33	7.94±0.49	9.24±0.97	1.048	0.24286	1.624	0.02162	1.847	0.00236	0.00236	pubmed
ENSMUSG00000041143	Tmco4	283.27±6.78	280.4±11.14	532.8±128.3	696.49±62.4	-0.015	0.98610	0.911	0.00002	1.298	0.00000	0.00000	pubmed

Ensgene	Symbol	HP	HD	ZP	ZD	LFC HD-HP	FDR HD-HP	LFC ZP-HP	FDR ZP-HP	LFC ZD-HP	FDR ZD-HP	Ratio	Links
ENSMUSG00000042359	Osbpl6	187.63±20.96	279.89±6.69	333.99±25.81	466.87±67.3	0.577	0.00528	0.831	0.00002	1.314	0.00000	0.00000	pubmed
ENSMUSG00000043789	Vwce	6.79±0.3	6.92±1.97	14.33±2.7	11.36±2.54	0.028	0.98878	1.074	0.03570	0.734	0.15050	0.15050	pubmed
ENSMUSG00000044252	Osbpl1a	91.03±2.07	120.09±9.52	161.2±4.01	182.41±21.67	0.399	0.02289	0.824	0.00000	1.000	0.00000	0.00000	pubmed
ENSMUSG00000044359	P2ry4	148.79±17.65	197.15±3.88	268.45±40.74	321.74±29.15	0.405	0.11745	0.851	0.00004	1.113	0.00000	0.00000	pubmed
ENSMUSG00000044986	Tst	166.18±6.43	156.12±11.5	306.03±50.69	242.92±26	-0.090	0.78679	0.880	0.00000	0.549	0.00074	0.00074	pubmed
ENSMUSG00000045019	Acer1	80.5±11.5	96.35±1.56	179.18±43.09	184.06±13.72	0.259	0.55965	1.154	0.00002	1.193	0.00000	0.00000	pubmed
ENSMUSG00000048440	Cyp4f16	547.4±40.78	505.32±37.94	1487.65±76.26	958.68±271.97	-0.115	0.79661	1.442	0.00000	0.808	0.00024	0.00024	pubmed
ENSMUSG00000051098	Mblac2	28.4±8.02	27.4±1.42	51.22±4.76	35.35±5.76	-0.055	0.94980	0.847	0.01023	0.316	0.40381	0.40381	pubmed
ENSMUSG00000051483	Cbr1	361.87±31.82	327.38±16.88	644.49±76.83	615.8±56.38	-0.145	0.56321	0.832	0.00000	0.766	0.00000	0.00000	pubmed
ENSMUSG00000053219	Raet1e	13.8±3.49	14.38±3.01	92.78±25.56	131.93±19.59	0.059	0.96806	2.748	0.00000	3.258	0.00000	0.00000	pubmed
ENSMUSG00000053279	Aldh1a1	1638.06±175.89	2284.58±85.08	3322.96±454.49	3362.52±560.56	0.480	0.06767	1.020	0.00000	1.037	0.00000	0.00000	pubmed
ENSMUSG00000054417	Cyp3a44	55.61±7.08	99.74±48.1	235.19±163.48	279.34±211.48	0.843	0.51343	2.081	0.02995	2.328	0.00478	0.00478	pubmed
ENSMUSG00000054619	Mettl7a1	218.62±11.62	257.07±27.18	396.57±30.17	374.25±34.55	0.234	0.25884	0.858	0.00000	0.776	0.00000	0.00000	pubmed
ENSMUSG00000054630	Ugt2b5	90.85±10.43	53.76±7.13	186.04±8.21	123.68±13.71	-0.759	0.00001	1.032	0.00000	0.442	0.00425	0.00425	pubmed
ENSMUSG00000055737	Ghr	254.42±12.37	348.49±51.73	501.35±84.98	466.23±31.23	0.454	0.02468	0.978	0.00000	0.874	0.00000	0.00000	pubmed
ENSMUSG00000056162	Cndp1	109.16±21.23	100.76±10.29	220.28±40.94	159.79±31.45	-0.116	0.85707	1.012	0.00142	0.548	0.09403	0.09403	pubmed
ENSMUSG00000057074	Ces1g	29.54±5.94	28.77±8.35	109.29±12.68	130.01±34.26	-0.038	0.96806	1.888	0.00000	2.138	0.00000	0.00000	pubmed
ENSMUSG00000058135	Gstm1	222.18±7.05	290.77±13.45	512.24±59.36	594.76±81.1	0.388	0.06034	1.205	0.00000	1.421	0.00000	0.00000	pubmed
ENSMUSG00000062410	Hsd3b3	142.47±19.6	129.86±14.16	446.08±59.79	326.01±34.3	-0.134	0.72144	1.646	0.00000	1.193	0.00000	0.00000	pubmed
ENSMUSG00000063730	Hsd3b2	36.03±2.36	36.13±6.91	90.57±13.92	74.08±12.33	0.005	0.99729	1.330	0.00000	1.037	0.00009	0.00009	pubmed
ENSMUSG00000067231	Cyp2c65	338.06±34.75	311.91±90.9	927.04±15.74	676.52±253.37	-0.116	0.88487	1.455	0.00007	1.000	0.00589	0.00589	pubmed
ENSMUSG00000068762	Gstm6	15.01±5.01	20.48±4.12	26.75±1.01	25.8±2.71	0.451	0.32570	0.836	0.01649	0.784	0.01417	0.01417	pubmed
ENSMUSG00000068874	Selenbp1	270.24±40.52	381.88±26.18	516.1±108.74	580.28±48.44	0.499	0.12127	0.933	0.00056	1.102	0.00001	0.00001	pubmed
ENSMUSG00000069727	Gm5595	27.64±3.1	29.53±1.08	49.32±6.92	36.5±3.93	0.096	0.89097	0.834	0.01175	0.403	0.25649	0.25649	pubmed
ENSMUSG00000069919	Hba-a1	27.34±8.95	40.23±5.26	66.41±28.88	37.11±6.25	0.556	0.41920	1.281	0.01288	0.440	0.45361	0.45361	pubmed
ENSMUSG00000070704	Ugt2b36	68.19±7.82	49.18±6.11	130.28±22.3	118.67±7.31	-0.472	0.04285	0.931	0.00000	0.796	0.00000	0.00000	pubmed
ENSMUSG00000071178	Serpina1b	152.29±13.27	188.07±33.76	344.01±48.7	448.37±61	0.304	0.18630	1.175	0.00000	1.559	0.00000	0.00000	pubmed
ENSMUSG00000071551	Akr1c19	246.13±18.99	171.06±35.41	646.48±119.64	439.31±79.93	-0.525	0.04522	1.393	0.00000	0.836	0.00007	0.00007	pubmed
ENSMUSG00000071604	Fam189a2	82.72±7.16	62.23±4.67	235.11±37.8	218.92±7.18	-0.411	0.15775	1.506	0.00000	1.403	0.00000	0.00000	pubmed
ENSMUSG00000074639	BC089597	4.66±1.09	6.09±4.66	14.31±1.69	15.01±0.5	0.387	0.67787	1.609	0.00070	1.683	0.00011	0.00011	pubmed
ENSMUSG00000079164	Tlr5	12.85±3.08	16.47±2.57	25.1±4.49	23.25±4.42	0.358	0.59435	0.966	0.03098	0.855	0.03694	0.03694	pubmed

Appendix Table S9

List of genes derived from the RNASEQ experiment shown in Fig. 2. Definitions of the categories see Fig. 2.

Ensgene	Symbol	HP	HD	ZP	ZD	LFC HD-HP	FDR HD-HP	LFC ZP-HP	FDR ZP-HP	LFC ZD-HP	FDR ZD-HP	Ratio	Links
ENSMUSG00000005533	Igf1r	68.19±8.08	68.34±6.31	70.95±11.86	104.74±17.01	0.004	0.99729	0.058	0.91950	0.622	0.01629	0.01629	pubmed
ENSMUSG00000005672	Kit	333±16.88	414.32±9.09	456±51.84	665.2±75.5	0.315	0.12661	0.453	0.01511	0.999	0.00000	0.00000	pubmed
ENSMUSG00000008136	Fhl2	227.91±3.75	329.12±33.51	292.54±36.07	526.55±73.66	0.530	0.01283	0.360	0.15794	1.208	0.00000	0.00000	pubmed
ENSMUSG00000020282	Rhbdf1	104.17±1.74	127.11±11.72	120.18±2.59	181.21±17.93	0.288	0.23232	0.206	0.44261	0.800	0.00000	0.00000	pubmed
ENSMUSG00000020427	Igfbp3	456.09±32.65	558.34±40.32	614.73±10.79	912.49±89.91	0.292	0.20749	0.431	0.03687	1.001	0.00000	0.00000	pubmed
ENSMUSG00000021846	Peli2	79.79±14.41	113.79±12.82	100.76±18.05	161.44±2.64	0.512	0.01232	0.336	0.17271	1.017	0.00000	0.00000	pubmed
ENSMUSG00000022180	Slc7a8	246.11±13.38	325.73±17.73	339.35±48.29	567.36±62.26	0.405	0.03811	0.463	0.01608	1.205	0.00000	0.00000	pubmed
ENSMUSG00000022231	Sema5a	457.64±33.71	660.35±86.97	688.97±58.48	993.94±104.15	0.529	0.00476	0.590	0.00190	1.119	0.00000	0.00000	pubmed
ENSMUSG00000022299	Slc25a32	115.19±6.61	188.54±17.28	101.93±3.82	276.96±21.24	0.711	0.00431	-0.176	0.66621	1.265	0.00000	0.00000	pubmed
ENSMUSG00000024533	Spire1	79.64±8	123.72±5.01	87.98±14.91	177.08±20.59	0.636	0.00882	0.143	0.72685	1.154	0.00000	0.00000	pubmed
ENSMUSG00000032754	Slc8b1	85.92±12.64	108.04±10.63	108.26±6.51	168.98±22.28	0.331	0.26425	0.333	0.26012	0.975	0.00000	0.00000	pubmed
ENSMUSG00000033857	Engase	173.35±8.05	214.19±17.39	181.03±21.93	335±39.36	0.305	0.17313	0.062	0.86166	0.949	0.00000	0.00000	pubmed
ENSMUSG00000040274	Cdk6	71.63±11.45	81.84±14.85	81.3±11.87	134.16±10.93	0.190	0.62023	0.181	0.63090	0.905	0.00001	0.00001	pubmed
ENSMUSG00000045411	2410002F23Rik	174.17±3.04	215.53±21.82	154.05±17.26	410.25±31.26	0.308	0.14881	-0.176	0.50575	1.236	0.00000	0.00000	pubmed
ENSMUSG00000048330	Ric3	20.34±1.36	30.56±5.97	28.15±5.36	53.87±5.8	0.587	0.21219	0.467	1.00000	1.406	0.00001	0.00001	pubmed
ENSMUSG00000049928	Glp2r	94.22±6.83	124.21±10.61	139.41±42.18	216.62±14.7	0.398	0.32003	0.565	0.11632	1.201	0.00001	0.00001	pubmed
ENSMUSG00000050846	Zfp623	91.36±9.61	119.18±19.4	120.71±15.58	206.78±12.79	0.383	0.12729	0.400	0.11177	1.177	0.00000	0.00000	pubmed
ENSMUSG00000052698	Tln2	162.7±14.5	249.14±17.46	221.2±30.75	395.45±38.7	0.615	0.00135	0.442	0.04646	1.282	0.00000	0.00000	pubmed
ENSMUSG00000053646	Plxnb1	68.95±9.1	109.4±21.11	84.97±20.17	219.17±10.72	0.665	0.00674	0.301	0.37152	1.668	0.00000	0.00000	pubmed
ENSMUSG00000061292	Cyp3a59	8.45±1.28	14.61±2.51	13.42±4.45	29.03±1.18	0.795	0.12383	0.667	0.23290	1.785	0.00000	0.00000	pubmed
ENSMUSG00000063760	Rnf217	76.93±13.04	132.53±7.24	66.02±4.47	194.18±11.54	0.784	0.00000	-0.222	0.36645	1.335	0.00000	0.00000	pubmed
ENSMUSG00000066513	Klk1b4	0±0	1.6±0.67	0.68±0.57	6.86±2.33	18.003	0.98842	16.772	1.00000	20.091	0.98074	0.98074	pubmed
ENSMUSG00000072294	Klf12	12.48±3.7	12.48±2.53	14.16±5.01	24.72±3.82	0.009	0.99729	0.181	0.80668	0.995	0.00681	0.00681	pubmed
ENSMUSG00000073700	Klhl21	118.11±8.71	137.34±22.62	127.43±4.18	194.99±22.44	0.217	0.43519	0.109	0.74346	0.724	0.00002	0.00002	pubmed
ENSMUSG00000073758	Sh3d21	49.71±7.65	59.03±8.89	82.43±16.1	126.87±3.65	0.249	0.59010	0.730	0.01960	1.352	0.00000	0.00000	pubmed
ENSMUSG00000079020	Slc45a4	160.1±2.88	211.75±20.62	167.07±12.68	335.8±26.89	0.404	0.01688	0.061	0.84073	1.070	0.00000	0.00000	pubmed
ENSMUSG00000083282	Ctsf	35.9±6.93	38.58±1.01	42.42±1.59	64.7±8.42	0.106	0.83200	0.242	0.51172	0.854	0.00009	0.00009	pubmed
ENSMUSG00000096956	Snhg18	104.96±10.2	130.61±11.97	140.83±20.03	211.44±5.68	0.315	0.27220	0.423	0.10292	1.010	0.00000	0.00000	pubmed

Appendix Table S10

List of genes derived from the RNASEQ experiment shown in Fig. 2. Definitions of the categories see Supplemental Information Appendix Fig. S1.

Ensgene	Symbol	HP	HD	ZP	ZD	LFC HD-HP	FDR HD-HP	LFC ZP-HP	FDR ZP-HP	LFC ZD-HP	FDR ZD-HP	Ratio	Links
ENSMUSG00000016496	Cd274	185.29±63.48	96.15±8.27	74.82±10.36	38.33±6.27	-0.946	0.00530	-1.307	0.00006	-2.270	0.00000	0.00000	pubmed
ENSMUSG00000026149	Tm4sf20	2207±479.4	1247.65±72	1227.02±123.77	709.46±94.56	-0.823	0.00017	-0.847	0.00019	-1.638	0.00000	0.00000	pubmed
ENSMUSG00000027225	Duoxa2	1309.53±112.73	706.63±38.55	137.84±21	50.57±24.97	-0.890	0.01757	-3.248	0.00000	-4.700	0.00000	0.00000	pubmed
ENSMUSG00000032033	Barx2	238.85±19.91	93.37±4.16	71.72±4.34	27.08±5.53	-1.355	0.00000	-1.736	0.00000	-3.133	0.00000	0.00000	pubmed
ENSMUSG00000034459	Ifit1	446.31±98.12	243.93±12.41	242.63±56.44	56.9±5.67	-0.871	0.00281	-0.880	0.00386	-2.970	0.00000	0.00000	pubmed
ENSMUSG00000039699	Batf2	270.42±8.12	90.51±5.2	127.48±23.56	36.61±1.35	-1.579	0.00000	-1.085	0.00000	-2.886	0.00000	0.00000	pubmed
ENSMUSG00000042677	Zc3h12a	1062.44±117.28	550.29±58.8	471.9±40.47	306.9±71.68	-0.949	0.01202	-1.171	0.00166	-1.792	0.00000	0.00000	pubmed
ENSMUSG00000050982	Apol10a	3336.26±168.28	1506.67±369.6	1559.24±232.11	528.85±87.2	-1.147	0.00000	-1.097	0.00002	-2.657	0.00000	0.00000	pubmed
ENSMUSG00000069874	Irgm2	780.32±235.97	439.54±22.57	313.31±53.28	193.3±42.75	-0.828	0.00175	-1.316	0.00000	-2.012	0.00000	0.00000	pubmed
ENSMUSG00000074345	Tnfaip8l3	171.3±34.89	89.81±14.3	78.86±2.4	48.55±14.65	-0.931	0.00616	-1.119	0.00092	-1.814	0.00000	0.00000	pubmed
ENSMUSG00000079339	Ifit1bl1	2556.61±547.59	1232.76±108.68	575.42±50.98	121.12±7.76	-1.052	0.00000	-2.152	0.00000	-4.400	0.00000	0.00000	pubmed

Appendix Table S11

List of genes derived from the RNASEQ experiment shown in Fig. 2. Definitions of the categories see Supplemental Information Appendix Fig. S1.

Ensgene	Symbol	HP	HD	ZP	ZD	LFC HD-HP	FDR HD-HP	LFC ZP-HP	FDR ZP-HP	LFC ZD-HP	FDR ZD-HP	Ratio	Links
ENSMUSG00000054072	Iigp1	368.29±94.76	182.56±4.78	158.97±14.73	125.48±30.19	-1.012	0.00000	-1.211	0.00000	-1.550	0.00000	0.00000	pubmed

Appendix Table S12

List of genes derived from the RNASEQ experiment shown in Fig. 2. Definitions of the categories see Supplemental Information Appendix Fig. S1.

Ensgene	Symbol	HP	HD	ZP	ZD	LFC HD-HP	FDR HD-HP	LFC ZP-HP	FDR ZP-HP	LFC ZD-HP	FDR ZD-HP	Ratio	Links
ENSMUSG00000020638	Cmpk2	361.17±45.35	152.78±7.27	442.06±45.92	100.31±15.66	-1.241	0.00000	0.291	0.30992	-1.850	0.00000	0.00000	pubmed
ENSMUSG00000022504	Ciita	507.5±148.46	194.95±95.89	247.23±123.42	74.42±9.65	-1.381	0.00604	-1.037	0.07282	-2.770	0.00000	0.00000	pubmed
ENSMUSG00000025017	Pik3ap1	447.39±36.57	248.34±12.88	291.36±65.32	94.44±25.65	-0.849	0.00006	-0.618	0.00964	-2.248	0.00000	0.00000	pubmed
ENSMUSG00000025993	Slc40a1	1315.19±231.92	720.26±40.16	850.82±101.5	420.15±31.1	-0.869	0.00000	-0.628	0.00033	-1.646	0.00000	0.00000	pubmed
ENSMUSG00000029449	Rhof	374.74±40.24	126.58±17.89	311.43±44.37	67.64±18.41	-1.566	0.00000	-0.267	0.52005	-2.474	0.00000	0.00000	pubmed
ENSMUSG00000031732	Phlpp2	938.6±38.91	524.69±75.73	836.18±41.25	286.92±51.98	-0.839	0.00000	-0.167	0.57802	-1.711	0.00000	0.00000	pubmed
ENSMUSG00000032038	St3gal4	10717.24±780	5737.95±364.83	10147.53±1023.15	3604.81±1211.49	-0.901	0.00002	-0.079	0.85768	-1.572	0.00000	0.00000	pubmed
ENSMUSG00000032380	Dapk2	202.94±12.16	110.14±8.55	148.68±9.88	64.74±11.89	-0.881	0.00096	-0.449	0.18494	-1.646	0.00000	0.00000	pubmed
ENSMUSG00000034422	Parp14	1622.15±267.6	909.06±86.47	1029.58±83.03	428.04±35.41	-0.835	0.00000	-0.656	0.00021	-1.922	0.00000	0.00000	pubmed
ENSMUSG00000039304	Tnfsf10	698.07±63.43	263.89±37.36	509.74±31	151.27±15.81	-1.403	0.00000	-0.453	0.02593	-2.205	0.00000	0.00000	pubmed
ENSMUSG00000041515	Irf8	1193.85±142.46	477.54±45.22	728.51±76.49	336.39±30.91	-1.322	0.00000	-0.712	0.00002	-1.827	0.00000	0.00000	pubmed
ENSMUSG00000044309	Apol7c	393.4±27.27	91.46±10.71	302.96±26.67	53.71±3.87	-2.104	0.00000	-0.377	0.06486	-2.871	0.00000	0.00000	pubmed
ENSMUSG00000048521	Cxcr6	52.29±5.84	26.06±2.08	34.59±4	11.69±1.97	-1.003	0.00224	-0.594	1.00000	-2.154	0.00000	0.00000	pubmed
ENSMUSG00000054520	Sh3bp2	206.16±14.74	112.87±9.4	138.83±28.08	74.49±17.06	-0.869	0.00009	-0.569	0.02500	-1.472	0.00000	0.00000	pubmed
ENSMUSG00000068299	1700019G17Rik	697.4±58.08	298.49±26.51	581.9±71.45	166.03±35.99	-1.225	0.00000	-0.261	0.34274	-2.073	0.00000	0.00000	pubmed
ENSMUSG00000079227	Ccr5	77.33±6.43	43.85±10.09	48.69±4.1	25.51±3.77	-0.817	0.00108	-0.666	0.01205	-1.598	0.00000	0.00000	pubmed

Appendix Table S13

List of genes derived from the RNASEQ experiment shown in Fig. 2. Definitions of the categories see Supplemental Information Appendix Fig. S1.

Ensgene	Symbol	HP	HD	ZP	ZD	LFC HD-HP	FDR HD-HP	LFC ZP-HP	FDR ZP-HP	LFC ZD-HP	FDR ZD-HP	Ratio	Links
ENSMUSG00000000317	Bcl6b	11.7±0.73	3.73±0.4	18.58±0.42	5.27±1.65	-1.651	0.01239	0.667	1.00000	-1.153	0.04556	0.04556	pubmed
ENSMUSG00000001288	Rarg	69.72±5.67	30.08±2.98	87±9.68	37.95±3.11	-1.213	0.00004	0.319	0.42633	-0.876	0.00228	0.00228	pubmed
ENSMUSG00000001493	Meox1	25.75±2.37	10.64±1.46	29.65±1.88	15.27±0.59	-1.275	0.00459	0.205	1.00000	-0.753	0.07738	0.07738	pubmed
ENSMUSG00000002603	Tgfb1	190.27±91.74	94.35±14.72	154.73±45.55	98.34±9.01	-1.012	0.00778	-0.297	0.60230	-0.952	0.00789	0.00789	pubmed
ENSMUSG00000002699	Lcp2	160.41±53.79	79.85±2.29	109.79±27.95	65.12±8.45	-1.006	0.00130	-0.545	0.16101	-1.301	0.00001	0.00001	pubmed
ENSMUSG00000003863	Ppfia3	370.37±22.98	193.76±14.49	510.11±72.78	199.58±40.12	-0.935	0.00002	0.462	0.08606	-0.893	0.00002	0.00002	pubmed
ENSMUSG00000004317	Clcn5	385.4±18.05	179.64±18.28	440.03±17.94	160.78±29.84	-1.101	0.00000	0.191	0.52459	-1.264	0.00000	0.00000	pubmed
ENSMUSG00000006403	Adamts4	46.34±11.71	17.01±2.15	36.46±10.01	31.12±3.36	-1.444	0.00140	-0.344	1.00000	-0.574	0.22788	0.22788	pubmed
ENSMUSG00000007872	Id3	990.51±70.23	342.25±31.89	853.94±18.55	348.23±14.3	-1.533	0.00000	-0.214	0.61158	-1.508	0.00000	0.00000	pubmed
ENSMUSG00000011263	Exoc3l2	42.46±9.3	9.83±3.14	39.88±9.29	12.59±1.77	-2.111	0.00000	-0.095	0.87513	-1.761	0.00000	0.00000	pubmed
ENSMUSG00000015839	Nfe2l2	1139.23±49.31	650.88±27.3	1299.26±33.88	731.51±27.45	-0.808	0.00000	0.190	0.25715	-0.639	0.00000	0.00000	pubmed
ENSMUSG00000015947	Fcgr1	39.67±8.16	22.61±0.53	25.84±0.43	24.48±2.94	-0.809	0.02688	-0.617	0.11814	-0.693	0.03851	0.03851	pubmed
ENSMUSG00000016477	E2f3	190.72±16.01	87.81±12.85	217.22±47.32	95.73±11.1	-1.119	0.00000	0.187	0.58459	-0.994	0.00000	0.00000	pubmed
ENSMUSG00000017386	Traf4	335.03±45.72	162±8.33	342.05±22.83	167±10.71	-1.048	0.00000	0.030	0.93115	-1.005	0.00000	0.00000	pubmed
ENSMUSG00000017607	Tns4	472.28±18.15	266.03±63.39	574.65±39.46	301.69±18.69	-0.828	0.00005	0.283	0.32766	-0.646	0.00149	0.00149	pubmed
ENSMUSG00000018899	Irf1	2185.57±254.12	994.74±52.91	1260.66±60.29	635.98±26.22	-1.136	0.00186	-0.794	0.06443	-1.781	0.00000	0.00000	pubmed
ENSMUSG00000018983	E2f2	505.3±75.66	205.5±11.82	402.71±27.95	166.26±23.22	-1.298	0.00000	-0.327	0.25413	-1.602	0.00000	0.00000	pubmed
ENSMUSG00000019966	Kitl	1137.72±77.27	491.67±15.68	1101.87±113.65	560.63±35.29	-1.210	0.00000	-0.046	0.87438	-1.021	0.00000	0.00000	pubmed
ENSMUSG00000020205	Phlda1	99.19±14.77	51.61±19.06	134.02±18.17	67.41±6.46	-0.943	0.01591	0.434	0.38485	-0.557	0.15625	0.15625	pubmed
ENSMUSG00000020258	Glyctk	107.59±10.16	43.06±8.19	68.1±13.67	34.4±7.4	-1.319	0.00000	-0.660	0.00202	-1.647	0.00000	0.00000	pubmed
ENSMUSG00000020682	Mmp28	56.46±3.52	29.77±3.87	46.23±6.03	34.14±4.57	-0.922	0.00385	-0.288	1.00000	-0.722	0.01756	0.01756	pubmed
ENSMUSG00000020961	Ston2	92.25±3.13	45.81±4.3	115.23±13.63	40.68±9.11	-1.010	0.00001	0.320	0.26131	-1.188	0.00000	0.00000	pubmed
ENSMUSG00000021070	Bdkrb2	39.8±1.12	21.79±4.78	59.7±8.82	30.56±0.74	-0.868	0.02372	0.584	1.00000	-0.381	0.32776	0.32776	pubmed
ENSMUSG00000021127	Zfp36l1	848.56±108.86	460.28±45.25	832.46±75.75	509.43±6.34	-0.882	0.00000	-0.027	0.94956	-0.736	0.00001	0.00001	pubmed
ENSMUSG00000021216	Tubal3	483.52±30.55	212.4±4.22	524.09±51.4	177.04±32.22	-1.187	0.00000	0.116	0.73279	-1.452	0.00000	0.00000	pubmed
ENSMUSG00000021281	Tnfaip2	275.27±70.8	147.09±15.52	162.91±23.29	119.53±6.83	-0.904	0.00565	-0.756	0.03529	-1.203	0.00004	0.00004	pubmed
ENSMUSG00000021411	Pxdc1	205.19±66.63	91.62±8.68	162.17±34.68	71.84±5.5	-1.163	0.00011	-0.338	0.44939	-1.514	0.00000	0.00000	pubmed
ENSMUSG00000021725	Parp8	53.18±20.16	26.62±3.58	52.88±16.93	29.84±2.08	-0.999	0.04763	-0.006	1.00000	-0.834	0.07152	0.07152	pubmed
ENSMUSG00000021822	Plau	86.46±13.3	22.89±3.57	125.45±29.99	33.8±13.37	-1.917	0.00000	0.537	0.26056	-1.349	0.00014	0.00014	pubmed
ENSMUSG00000021944	Gata4	21.68±14.49	6.37±2.31	10.76±8.23	1.76±0.37	-1.762	0.00563	-1.014	0.15364	-3.625	0.00000	0.00000	pubmed
ENSMUSG00000021974	Fgf9	29.7±4.49	13.83±0.62	39.55±6.83	15.48±2.1	-1.102	0.01719	0.414	1.00000	-0.938	0.02765	0.02765	pubmed
ENSMUSG00000021994	Wnt5a	124.24±27.78	69.38±10	186.64±35.59	102.5±12.14	-0.841	0.01084	0.586	0.11177	-0.277	0.46065	0.46065	pubmed
ENSMUSG00000022074	Tnfrsf10b	42.16±1.33	19.96±1.68	39.13±4.25	23.88±4.51	-1.079	0.00210	-0.106	1.00000	-0.822	0.01366	0.01366	pubmed
ENSMUSG00000022218	Tgm1	15.5±3.71	5.05±2.07	8.12±3.9	5.02±0.77	-1.615	0.00548	-0.926	0.12790	-1.626	0.00244	0.00244	pubmed
ENSMUSG00000022237	Ankrd33b	11.99±7.89	5.07±1.39	9.9±3.99	4.48±0.86	-1.241	0.03856	-0.265	0.73078	-1.409	0.00923	0.00923	pubmed
ENSMUSG00000022434	Fam118a	390.63±40.96	171.17±20.74	459.62±39.35	137.37±27.81	-1.190	0.00000	0.234	0.48566	-1.510	0.00000	0.00000	pubmed
ENSMUSG00000022489	Pde1b	102.65±21.58	57.2±11.25	86.28±3.34	57.48±6.08	-0.843	0.01737	-0.250	0.63008	-0.835	0.01036	0.01036	pubmed
ENSMUSG00000022676	Snai2	16.64±1.83	6.38±0.6	20.84±2.12	8.79±0.69	-1.380	0.00378	0.326	0.54014	-0.922	0.03063	0.03063	pubmed
ENSMUSG00000022768	Ccdc116	61.87±6.15	28.45±2.69	61.74±7.83	24.81±2.61	-1.120	0.00033	-0.002	1.00000	-1.318	0.00001	0.00001	pubmed
ENSMUSG00000022793	B4galt4	549.46±52.51	298.11±7.38	560.17±34.11	271.3±25.61	-0.883	0.00000	0.027	0.93358	-1.020	0.00000	0.00000	pubmed
ENSMUSG00000022864	D16Ertd472e	37.12±10.21	20.74±2.22	23.02±4.94	17.95±2.04	-0.837	0.02353	-0.684	0.08095	-1.042	0.00167	0.00167	pubmed
ENSMUSG00000022999	Lmbr1l	326.61±28.12	186.17±14.55	338.65±21.94	183.03±5	-0.810	0.00000	0.053	0.87438	-0.835	0.00000	0.00000	pubmed
ENSMUSG00000023755	Rhebl1	88.25±13.34	23.69±2.32	109.31±22.51	22.76±3.5	-1.897	0.00000	0.309	0.50880	-1.953	0.00000	0.00000	pubmed
ENSMUSG00000023947	Nfkbie	110.2±54.83	39.23±13.34	92.4±23.3	39.61±7.14	-1.491	0.00203	-0.253	0.75800	-1.478	0.00122	0.00122	pubmed
ENSMUSG00000024014	Pim1	513.87±111.25	235.46±22.31	378.35±26.82	193.46±31.92	-1.126	0.01317	-0.442	0.48768	-1.410	0.00050	0.00050	pubmed
ENSMUSG00000024349	Tmem173	182.77±70.9	100.89±3.66	114.34±45.41	69.38±5.65	-0.857	0.03922	-0.675	0.14733	-1.397	0.00006	0.00006	pubmed
ENSMUSG00000024401	Tnf	27.79±9.31	5.05±2.27	8.31±3.46	2.78±1.62	-2.459	0.00994	-1.738	1.00000	-3.329	0.00026	0.00026	pubmed
ENSMUSG00000024431	Nr3c1	1939.3±102.37	1020.63±63.49	2061.76±66.8	829.54±21	-0.926	0.00000	0.088	0.49848	-1.225	0.00000	0.00000	pubmed
ENSMUSG00000024440	Pcdh12	39.2±7.83	11.95±2.74	54.52±6.43	21.79±2.36	-1.714	0.00000	0.472	0.17081	-0.849	0.00506	0.00506	pubmed
ENSMUSG00000024663	Rab3il1	37.09±6.11	17.58±2.49	37.67±4.93	20.22±1.9	-1.076	0.00158	0.024	0.97781	-0.870	0.00668	0.00668	pubmed
ENSMUSG00000024737	Slc15a3	90.53±25.41	38.9±7.35	67.29±23.39	33.95±2.72	-1.219	0.00031	-0.427	0.35934	-1.413	0.00001	0.00001	pubmed
ENSMUSG00000025083	Afap1l2	37.1±7.08	14.07±3.1	47.12±7.11	16.25±1.38	-1.398	0.00029	0.344	1.00000	-1.191	0.00111	0.00111	pubmed
ENSMUSG00000025402	Nab2	80.95±18.95	33.52±4.45	91.9±5.61	31.28±6.31	-1.271	0.00001	0.184	0.68823	-1.376	0.00000	0.00000	pubmed
ENSMUSG00000025804	Ccr1	125.11±7.81	62±3.41	78.98±18.26	57.9±10.94	-1.013	0.00047	-0.662	0.04770	-1.108	0.00005	0.00005	pubmed
ENSMUSG00000025856	Pdgfa	223.85±11.9	120.57±4.07	292.84±35.64	144.27±3.76	-0.893	0.00001	0.387	0.11709	-0.634	0.00145	0.00145	pubmed
ENSMUSG00000026227	2810459M11Rik	93.87±5.79	45.47±4.6	110.96±18.73	57.6±13.77	-1.045	0.00000	0.240	0.40835	-0.698	0.00075	0.00075	pubmed
ENSMUSG00000026447	Pik3c2b	610.31±47.87	276.59±17.61	614.94±0.51	239.5±36.49	-1.141	0.00000	0.011	0.98278	-1.348	0.00000	0.00000	pubmed
ENSMUSG00000026452	Syt2	43.35±12.06	12±2.98	82.76±11.09	20.44±3.66	-1.855	0.00003	0.931	1.00000	-1.083	0.01269	0.01269	pubmed
ENSMUSG00000026580	Selp	27.61±16.95	5.35±2.7	17.81±4.98	8.06±1.18	-2.370	0.00132	-0.630	1.00000	-1.778	0.01001	0.01001	pubmed
ENSMUSG00000027173	Depdc7	190.39±34.14	106.37±9.3	236.26±17.08	116.85±23.94	-0.840	0.00014	0.311	0.29573	-0.707	0.00105	0.00105	pubmed
ENSMUSG00000027313	Chac1	34.07±10.81	8.52±0.43	9.59±3.05	4.76±0.71	-1.999	0.02127	-1.831	1.00000	-2.839	0.00036	0.00036	pubmed
ENSMUSG00000027314	Dll4	219.42±20.39	96.66±9.86	175.97±10.07	97.16±0.35	-1.183	0.00000	-0.318	0.16980	-1.175	0.00000	0.00000	pubmed
ENSMUSG00000027356	Fermt1	748.26±47.28	425.34±17.94	665.99±12.71	449.13±27.12	-0.815	0.00000	-0.168	0.28492	-0.736	0.00000	0.00000	pubmed
ENSMUSG00000027398	Il1b	51.08±16.03	3.99±1.95	20.3±4.29	3.23±1.24	-3.677	0.00000	-1.329	1.00000	-3.976	0.00000	0.00000	pubmed
ENSMUSG00000027399	Il1a	10.12±2.81	2.4±0.68	6.97±2.66	0.25±0.36	-2.074	0.00770	-0.527	0.53608	-5.332	0.00256	0.00256	pubmed
ENSMUSG00000027457	Snph	158.68±9.06	76.33±5.48	167.98±12.98	95.1±10.54	-1.055	0.00000	0.082	0.82227	-0.737	0.00009	0.00009	pubmed
ENSMUSG00000027983	Cyp2u1	31.37±3.76	12±4.15	29.56±5.67	12.78±1.18	-1.388	0.00049	-0.087	1.00000	-1.296	0.00055	0.00055	pubmed
ENSMUSG00000028525	Pde4b	234.74±75.72	97.34±20.7	162.61±45.89	73.59±8.74	-1.270	0.00107	-0.529	0.31797	-1.672	0.00000	0.00000	pubmed
ENSMUSG00000028635	Edn2	118±10.29	23.38±5.38	168.25±17.57	28.82±8.35	-2.335	0.00000	0.511	0.18054	-2.039	0.00000	0.00000	pubmed
ENSMUSG00000028681	Ptch2	8.43±0.17	0.8±0.01	10.69±4.11	1.74±0.28	-3.400	0.00359	0.341	1.00000	-2.267	0.00612	0.00612	pubmed
ENSMUSG00000028919	Arhgef19	271.46±28.08	145.97±12.96	372.92±24.3	182.3±14.81	-0.895	0.00060	0.458	0.16200	-0.574	0.03134	0.03134	pubmed

Ensgene	Symbol	HP	HD	ZP	ZD	LFC HD-HP	FDR HD-HP	LFC ZP-HP	FDR ZP-HP	LFC ZD-HP	FDR ZD-HP	Ratio	Links
ENSMUSG00000029188	Slc34a2	6661.31±494.95	2445.98±483.94	8134.43±478.88	1615.39±691.16	-1.445	0.00001	0.288	0.60036	-2.044	0.00000	0.00000	pubmed
ENSMUSG00000029401	Rilpl2	179.21±19.23	91.31±4.02	127.07±7.57	78.84±3.17	-0.972	0.00000	-0.495	0.02530	-1.184	0.00000	0.00000	pubmed
ENSMUSG00000029923	Rab19	187.31±17.41	106.85±17.65	154.96±16.37	79.93±5.99	-0.809	0.00037	-0.274	0.39670	-1.227	0.00000	0.00000	pubmed
ENSMUSG00000030256	Bhlhe41	47.86±18.91	14.99±8.79	31.42±15.77	6.82±1.86	-1.677	0.03016	-0.606	1.00000	-2.816	0.00004	0.00004	pubmed
ENSMUSG00000030494	Rhpn2	706.24±35.24	369.85±27.28	727.05±19.53	270.34±21.06	-0.933	0.00000	0.042	0.89340	-1.386	0.00000	0.00000	pubmed
ENSMUSG00000030551	Nr2f2	128.74±11.87	70.22±3.3	137.9±11.16	85.22±6.57	-0.873	0.00001	0.100	0.76743	-0.595	0.00217	0.00217	pubmed
ENSMUSG00000031015	Swap70	455.91±179.6	254.51±64.7	427.33±154.72	251.28±26.52	-0.841	0.03884	-0.093	0.90166	-0.859	0.01894	0.01894	pubmed
ENSMUSG00000031530	Dusp4	57.86±13.24	30.09±2.54	52.05±16.94	24.86±4.58	-0.943	0.01895	-0.152	0.82076	-1.214	0.00073	0.00073	pubmed
ENSMUSG00000031778	Cx3cl1	888.83±77.35	473.93±25.01	735.93±58.67	419.65±59.27	-0.907	0.00000	-0.272	0.21313	-1.084	0.00000	0.00000	pubmed
ENSMUSG00000031880	Rrad	22.59±10.27	8.25±0.79	13.6±0.93	5±0.85	-1.451	0.03434	-0.730	1.00000	-2.173	0.00053	0.00053	pubmed
ENSMUSG00000031937	Vstm5	223.19±12.68	97.28±13.99	281.75±35.35	99.82±21.73	-1.198	0.00000	0.336	0.32000	-1.163	0.00000	0.00000	pubmed
ENSMUSG00000032014	Oaf	210.06±20.79	103.77±4.81	238.46±31.74	125.58±19.61	-1.018	0.00000	0.182	0.46799	-0.745	0.00000	0.00000	pubmed
ENSMUSG00000032122	Slc37a2	31.35±0.63	14.66±3.65	27.98±6.05	16.45±3.82	-1.099	0.00716	-0.162	1.00000	-0.927	0.01451	0.01451	pubmed
ENSMUSG00000032313	AI118078	16.67±3.48	2.13±1.35	6.78±0.8	2.48±0.84	-2.967	0.00004	-1.293	1.00000	-2.736	0.00003	0.00003	pubmed
ENSMUSG00000032714	Syde1	127.81±5.72	51.86±3.15	128.54±11.21	57.14±5.34	-1.301	0.00000	0.008	0.98828	-1.158	0.00000	0.00000	pubmed
ENSMUSG00000032860	P2ry2	311.03±37.07	148.7±6.1	292.85±21.97	116.78±20.67	-1.065	0.00000	-0.087	0.81948	-1.416	0.00000	0.00000	pubmed
ENSMUSG00000032902	Slc16a1	782.88±74.65	421.22±10.89	862.27±91.08	480.46±35.5	-0.894	0.00000	0.139	0.64041	-0.705	0.00004	0.00004	pubmed
ENSMUSG00000033949	Trim36	118.17±9.75	66.27±1.4	110.6±12.06	61.93±4.64	-0.835	0.00013	-0.096	0.80861	-0.933	0.00001	0.00001	pubmed
ENSMUSG00000034159	2310007B03Rik	77.23±1.69	24.18±4.72	96.03±9.84	26.88±2.02	-1.674	0.00000	0.314	1.00000	-1.525	0.00000	0.00000	pubmed
ENSMUSG00000034226	Rhov	28.81±5.89	11.15±2.89	41.83±5.34	14.28±6.08	-1.368	0.02268	0.537	1.00000	-1.022	0.06795	0.06795	pubmed
ENSMUSG00000034282	Evpl	475.09±26.05	128.69±24.6	598.8±88.09	158.7±11.14	-1.884	0.00000	0.334	0.23218	-1.582	0.00000	0.00000	pubmed
ENSMUSG00000034413	Neurl1b	184.41±10.24	69.67±5.28	216.69±27.37	85.74±13.04	-1.404	0.00000	0.233	0.49202	-1.105	0.00000	0.00000	pubmed
ENSMUSG00000034584	Exph5	456.58±112.83	191.54±7.46	342.02±23.66	166.3±14.84	-1.253	0.00000	-0.417	0.16313	-1.456	0.00000	0.00000	pubmed
ENSMUSG00000034645	Zyg11a	17.08±5.21	4.51±2.61	31.91±10.41	4.54±0.79	-1.918	0.00100	0.899	0.07830	-1.919	0.00043	0.00043	pubmed
ENSMUSG00000034652	Cd300a	41.92±3.19	22.86±4.06	38.25±2.83	27.12±5.14	-0.873	0.00826	-0.131	0.80520	-0.623	0.04696	0.04696	pubmed
ENSMUSG00000034765	Dusp5	89.79±4.67	49.72±5.17	114.77±7.64	60.92±3.25	-0.853	0.00011	0.353	0.17921	-0.561	0.01035	0.01035	pubmed
ENSMUSG00000034855	Cxcl10	86.62±26.18	9.32±1.55	29.61±6.24	5.47±1.36	-3.216	0.00139	-1.548	0.22662	-3.982	0.00003	0.00003	pubmed
ENSMUSG00000035105	Egln3	292.61±13.8	160.67±29.94	385.36±72.57	186.58±27.34	-0.865	0.00006	0.397	0.14689	-0.650	0.00253	0.00253	pubmed
ENSMUSG00000035172	Plekhh3	175.81±11.75	77.64±7.63	168.29±9.7	73.05±5.06	-1.179	0.00000	-0.064	0.87471	-1.269	0.00000	0.00000	pubmed
ENSMUSG00000035385	Ccl2	23.86±13.04	0.53±0.38	11.56±7.72	0±0	-5.485	0.00011	-1.045	1.00000	-21.935	0.97933	0.97933	pubmed
ENSMUSG00000035606	Ky	35.96±5.11	4.77±1.69	37.31±4.03	4.85±2.43	-2.911	0.00000	0.053	1.00000	-2.909	0.00000	0.00000	pubmed
ENSMUSG00000036377	C530008M17Rik	1658.89±98.13	937.26±90.38	1530.47±155.68	707.96±105.01	-0.824	0.00000	-0.116	0.71117	-1.229	0.00000	0.00000	pubmed
ENSMUSG00000036381	P2ry14	88.68±11.51	40.19±2.67	90.59±13.01	59.13±8.95	-1.142	0.00001	0.030	0.96103	-0.583	0.02591	0.02591	pubmed
ENSMUSG00000036533	Cdc42ep3	127.73±15.66	68.81±11.46	149.59±9.76	94.11±4.79	-0.892	0.00006	0.227	0.48293	-0.441	0.05893	0.05893	pubmed
ENSMUSG00000036553	Sh3tc1	102.35±17.62	33.81±4.43	90.17±10.54	31.18±3.37	-1.597	0.00000	-0.181	0.61504	-1.710	0.00000	0.00000	pubmed
ENSMUSG00000036718	Micall2	176.77±21.39	84.57±5.84	175.07±14.05	85.37±13.07	-1.064	0.00000	-0.015	0.98263	-1.048	0.00000	0.00000	pubmed
ENSMUSG00000036894	Rap2b	157.5±18.3	84.02±7.68	161.78±12.51	81.1±16.87	-0.906	0.00001	0.040	0.93358	-0.960	0.00000	0.00000	pubmed
ENSMUSG00000036959	Bcorl1	123.74±8.34	63.32±5.49	145.98±10.66	72.24±5.61	-0.967	0.00000	0.237	0.35758	-0.775	0.00002	0.00002	pubmed
ENSMUSG00000036995	Asap3	40.62±7.65	17±3.15	32.8±3.61	9.24±2.78	-1.253	0.00068	-0.306	0.55607	-2.132	0.00000	0.00000	pubmed
ENSMUSG00000037405	Icam1	197.71±84.65	55.47±14	149.37±40.32	38.64±6.54	-1.834	0.00317	-0.404	0.68779	-2.355	0.00003	0.00003	pubmed
ENSMUSG00000037447	Arid5a	160.84±73.59	77.32±16.07	106.18±45.53	61.15±4.35	-1.057	0.04333	-0.598	0.35660	-1.395	0.00198	0.00198	pubmed
ENSMUSG00000037465	Klf10	319.63±33.03	149.44±17.75	297.79±12.89	185.94±2.67	-1.097	0.00088	-0.102	0.87605	-0.782	0.01819	0.01819	pubmed
ENSMUSG00000037722	Gnpnat1	767.49±99.22	438.33±8.76	635.57±89.21	355.81±41	-0.808	0.00000	-0.272	0.21196	-1.108	0.00000	0.00000	pubmed
ENSMUSG00000037731	Themis2	110.46±48.35	52.74±5.42	72.1±28.71	31.73±7.92	-1.066	0.01827	-0.613	0.27137	-1.801	0.00000	0.00000	pubmed
ENSMUSG00000037752	Xkr8	40.29±5.24	17.83±0.34	28.6±2.05	17.01±3.26	-1.176	0.00006	-0.495	0.14424	-1.242	0.00001	0.00001	pubmed
ENSMUSG00000037872	Ackr1	34.55±11.43	9.59±1.17	25.77±3.32	9.04±0.84	-1.849	0.00003	-0.420	1.00000	-1.935	0.00001	0.00001	pubmed
ENSMUSG00000037892	Pcdh18	28.64±8.2	8.52±3.26	40.62±11.14	8.53±1.38	-1.752	0.00076	0.502	1.00000	-1.750	0.00036	0.00036	pubmed
ENSMUSG00000037902	Sirpa	258.82±64.06	137.1±14.54	226.63±41.64	133.21±16.74	-0.917	0.00081	-0.191	0.66945	-0.958	0.00022	0.00022	pubmed
ENSMUSG00000037992	Rara	274.13±19.97	156.15±6.57	301.69±23.05	155.25±15.88	-0.812	0.00001	0.138	0.64223	-0.822	0.00000	0.00000	pubmed
ENSMUSG00000037995	Igsf9	946.95±91	159.93±23.58	1328.88±169.53	226.78±62.85	-2.566	0.00000	0.489	0.13290	-2.063	0.00000	0.00000	pubmed
ENSMUSG00000038037	Socs1	80.78±13.19	16.5±1.3	29.68±2.1	10.46±5.45	-2.291	0.00000	-1.444	1.00000	-2.963	0.00000	0.00000	pubmed
ENSMUSG00000038167	Plekhg6	445.08±34.02	247.19±30.42	559.78±46.24	165.39±54.17	-0.848	0.00488	0.331	0.43393	-1.430	0.00000	0.00000	pubmed
ENSMUSG00000038422	Hdhd3	305.92±36.48	172.71±5.33	252.44±23.65	142.56±7.68	-0.825	0.00002	-0.278	0.30992	-1.102	0.00000	0.00000	pubmed
ENSMUSG00000038451	Spsb2	109.98±11.08	61.26±6.11	103.65±3.9	56.47±8.84	-0.844	0.00004	-0.085	0.82316	-0.966	0.00000	0.00000	pubmed
ENSMUSG00000038692	Hoxb4	41.51±9	22.84±4.14	49.16±4.25	38.48±4.84	-0.862	0.03183	0.243	1.00000	-0.110	0.83529	0.83529	pubmed
ENSMUSG00000039339	2010001E11Rik	145.55±10.62	52.71±9.26	171.5±16.31	64.65±19.51	-1.465	0.00000	0.237	0.63088	-1.175	0.00007	0.00007	pubmed
ENSMUSG00000039741	Bahcc1	197.77±34.44	113.31±5.56	189.17±10.84	109.9±10.5	-0.803	0.00041	-0.064	0.88978	-0.846	0.00009	0.00009	pubmed
ENSMUSG00000039835	Nhsl1	915.75±32.5	473.07±28.89	1041.72±56.23	386.89±48.1	-0.953	0.00000	0.186	0.55036	-1.244	0.00000	0.00000	pubmed
ENSMUSG00000039981	Zc3h12d	137.89±25.64	67.15±12.55	136.5±28.41	60.76±8.89	-1.038	0.00264	-0.014	0.98899	-1.180	0.00023	0.00023	pubmed
ENSMUSG00000039989	Cbx4	124.35±18.28	70.08±9.94	108.72±25.65	64.83±4.49	-0.828	0.01138	-0.193	0.70488	-0.939	0.00160	0.00160	pubmed
ENSMUSG00000040740	Slc25a34	109.8±34.89	48.35±20.83	106.17±16.2	36.9±17.27	-1.182	0.02214	-0.048	0.96859	-1.577	0.00054	0.00054	pubmed
ENSMUSG00000041378	Cldn5	34.35±3.4	15.12±5.45	40.58±5.39	23.34±4.91	-1.182	0.00067	0.239	0.61090	-0.551	0.10624	0.10624	pubmed
ENSMUSG00000041479	Syt15	26.66±2.21	11.47±4.75	30.7±5.01	11.48±3.3	-1.219	0.02619	0.202	1.00000	-1.214	0.01441	0.01441	pubmed
ENSMUSG00000042379	Esm1	24.78±2.16	10.09±3.02	39.39±8.05	13.31±0.88	-1.295	0.01968	0.667	1.00000	-0.899	0.08287	0.08287	pubmed
ENSMUSG00000042745	Id1	781.85±87.32	362.37±45.71	753.29±106.61	473.41±10.8	-1.109	0.00131	-0.054	0.94670	-0.724	0.04035	0.04035	pubmed
ENSMUSG00000042810	Krba1	164.16±11.97	92.34±0.25	236.83±11.18	106.01±11.28	-0.831	0.00000	0.528	0.00022	-0.634	0.00001	0.00001	pubmed
ENSMUSG00000044294	Krt84	35.31±13.73	8.25±0.33	15.05±4.12	3.3±2.28	-2.099	0.00239	-1.233	1.00000	-3.432	0.00000	0.00000	pubmed
ENSMUSG00000044338	Aplnr	52.54±5.8	10.93±3.85	64.7±8.46	10.19±2.22	-2.266	0.00000	0.301	1.00000	-2.359	0.00000	0.00000	pubmed
ENSMUSG00000044548	Dact1	63.51±8.97	26.32±4.91	62.83±8.24	30.65±7.62	-1.271	0.00036	-0.016	0.98828	-1.049	0.00228	0.00228	pubmed
ENSMUSG00000044707	Ccnjl	74.12±9.71	19.16±1.68	65.07±14.06	17.62±2.24	-1.952	0.00000	-0.189	1.00000	-2.076	0.00000	0.00000	pubmed
ENSMUSG00000044708	Kcnj10	44.25±11.23	23.92±4.39	44.18±1.22	28.04±0.4	-0.889	0.02138	-0.004	1.00000	-0.660	0.06896	0.06896	pubmed

Ensgene	Symbol	HP	HD	ZP	ZD	LFC HD-HP	FDR HD-HP	LFC ZP-HP	FDR ZP-HP	LFC ZD-HP	FDR ZD-HP	Ratio	Links
ENSMUSG00000044921	Rassf9	12.23±3.9	3.45±0.97	13.9±5.92	6.77±0.28	-1.821	0.02670	0.184	1.00000	-0.852	0.25741	0.25741	pubmed
ENSMUSG00000045314	Sowahb	648.44±41.04	309.34±23.26	563.93±37.31	253.82±47.11	-1.068	0.00000	-0.202	0.49534	-1.355	0.00000	0.00000	pubmed
ENSMUSG00000045664	Cdc42ep2	218.15±16.57	62.73±7.73	355.18±21.06	74.43±21.85	-1.797	0.00000	0.703	0.00812	-1.555	0.00000	0.00000	pubmed
ENSMUSG00000045680	Tcf21	118.07±18.99	29.78±3.37	155.67±17.79	45.96±5.46	-1.988	0.00000	0.398	0.20001	-1.360	0.00000	0.00000	pubmed
ENSMUSG00000045930	Clec14a	55.48±3.74	26.84±5.44	68.24±3.68	34.73±4.93	-1.046	0.00004	0.299	0.34059	-0.680	0.00560	0.00560	pubmed
ENSMUSG00000045932	Ifit2	138.4±32.39	68.18±8.45	88.51±9.53	63.18±4.98	-1.021	0.00002	-0.644	0.01623	-1.131	0.00000	0.00000	pubmed
ENSMUSG00000045991	Onecut2	7.53±2.12	2.39±1.29	3.18±0.87	3.26±0.94	-1.645	0.02604	-1.239	0.07227	-1.202	0.04931	0.04931	pubmed
ENSMUSG00000046245	Pilra	44.76±4.68	24.99±3.08	31.05±0.62	31.09±7.58	-0.839	0.01232	-0.527	0.16793	-0.520	0.10890	0.10890	pubmed
ENSMUSG00000046470	Sox18	42.9±8.47	20.15±9.39	60.26±1.23	23.91±3.93	-1.089	0.00265	0.489	0.24097	-0.841	0.01451	0.01451	pubmed
ENSMUSG00000046916	Myct1	22.06±4.18	6.4±1.8	30.91±3.68	10.81±1.19	-1.785	0.00004	0.487	0.24519	-1.031	0.00649	0.00649	pubmed
ENSMUSG00000047330	Kcne4	117.1±50.77	49.94±13.89	64.84±21.85	59.53±7.94	-1.228	0.02644	-0.852	0.18646	-0.975	0.06327	0.06327	pubmed
ENSMUSG00000047496	Rnf152	1944.87±204.82	623.23±77.8	2018.64±153.14	464.94±88.46	-1.642	0.00000	0.054	0.91898	-2.065	0.00000	0.00000	pubmed
ENSMUSG00000047821	Trim16	377.5±5.58	160.24±24.17	531.63±68.34	178.43±15.27	-1.236	0.00000	0.494	0.02689	-1.082	0.00000	0.00000	pubmed
ENSMUSG00000048458	Fam212b	35.04±4.01	15.44±1.41	35.38±2.19	23.4±4.89	-1.183	0.00460	0.013	1.00000	-0.579	0.16428	0.16428	pubmed
ENSMUSG00000048897	Zfp710	426.33±9.34	236.02±5.8	385.64±9.95	187.68±11.88	-0.853	0.00000	-0.145	0.52458	-1.184	0.00000	0.00000	pubmed
ENSMUSG00000048911	Rnf24	165.6±19.42	89.72±11.89	139.66±31.29	57.92±8.68	-0.885	0.02608	-0.246	0.67999	-1.517	0.00001	0.00001	pubmed
ENSMUSG00000049103	Ccr2	77.1±13.47	32.19±3.63	46.21±11.7	21.99±1.25	-1.260	0.00026	-0.736	0.06816	-1.808	0.00000	0.00000	pubmed
ENSMUSG00000050010	Shisa3	35±21.75	5.6±2.28	23.46±15.13	5.52±2.36	-2.644	0.00635	-0.575	1.00000	-2.666	0.00314	0.00314	pubmed
ENSMUSG00000050014	Apol10b	69.41±5.6	34.31±7.88	48.19±7.17	23.12±4.25	-1.017	0.00457	-0.526	1.00000	-1.582	0.00000	0.00000	pubmed
ENSMUSG00000050578	Mmp13	93.77±11.42	38.84±5.5	74.03±5.94	40.04±6.95	-1.271	0.00000	-0.341	0.37152	-1.223	0.00000	0.00000	pubmed
ENSMUSG00000050592	Fam78a	326.05±33.32	110.87±19.06	307.94±23.11	78.8±18.5	-1.557	0.00000	-0.082	0.85598	-2.052	0.00000	0.00000	pubmed
ENSMUSG00000050721	Plekho2	168.67±46.1	81.96±7.23	129.22±19.87	59.9±6.14	-1.040	0.00003	-0.383	0.26496	-1.491	0.00000	0.00000	pubmed
ENSMUSG00000050737	Ptges	85.92±15.54	45.68±9.76	99.1±12.27	49.09±7.31	-0.910	0.00752	0.205	0.69157	-0.806	0.01226	0.01226	pubmed
ENSMUSG00000050910	Cdr2l	35.4±3.14	19.67±2.46	39.28±11.05	27.35±1.22	-0.846	0.02841	0.150	1.00000	-0.373	0.33619	0.33619	pubmed
ENSMUSG00000051065	Mb21d2	223.09±29.23	127.48±11.44	244.25±27.64	126.94±13.22	-0.807	0.00003	0.131	0.68802	-0.814	0.00001	0.00001	pubmed
ENSMUSG00000052135	Foxo6	103.2±7.12	49.57±7.56	68.05±9.11	33.34±10.5	-1.059	0.00182	-0.601	0.14536	-1.634	0.00000	0.00000	pubmed
ENSMUSG00000052270	Fpr2	7.3±2.43	2.13±0.39	3.84±1.24	2.02±0.42	-1.772	0.04514	-0.918	1.00000	-1.858	0.01836	0.01836	pubmed
ENSMUSG00000052485	Tmem171	150.36±31.33	69.69±7.98	233.79±4.32	107.26±18.3	-1.109	0.00002	0.637	0.03139	-0.486	0.08684	0.08684	pubmed
ENSMUSG00000053004	Hrh1	75.5±5.08	42.61±3.35	81.71±9.33	32.75±4.6	-0.826	0.00442	0.113	1.00000	-1.210	0.00001	0.00001	pubmed
ENSMUSG00000053063	Clec12a	15.2±3.89	5.58±1.1	8.93±6.28	5.21±1.47	-1.445	0.01103	-0.755	0.22065	-1.535	0.00336	0.00336	pubmed
ENSMUSG00000053113	Socs3	451.02±180.87	111.29±11.41	155.18±51.28	47.84±4.35	-2.019	0.00789	-1.539	0.07740	-3.237	0.00000	0.00000	pubmed
ENSMUSG00000053175	Bcl3	408.87±36.23	83.86±14.66	266.32±30.42	62.81±14.12	-2.286	0.00000	-0.618	0.16483	-2.703	0.00000	0.00000	pubmed
ENSMUSG00000053199	Arhgap20	38.37±9.83	16.21±2.59	35.18±5.74	27±1.92	-1.242	0.00520	-0.126	1.00000	-0.506	0.27215	0.27215	pubmed
ENSMUSG00000053846	Lipg	61.8±0.72	27.13±2.71	41.94±1.4	18.87±10.83	-1.187	0.00018	-0.559	0.14673	-1.694	0.00000	0.00000	pubmed
ENSMUSG00000054855	Rnd1	44.75±28.86	4.24±2.26	24.6±15.16	3.36±3.2	-3.398	0.00280	-0.863	0.60239	-3.748	0.00049	0.00049	pubmed
ENSMUSG00000056306	Sertm1	35.35±5.53	9.32±1.07	39.02±1.53	7.26±0.24	-1.924	0.00001	0.142	1.00000	-2.283	0.00000	0.00000	pubmed
ENSMUSG00000057103	Cml1	23.87±2.42	12.49±1.89	31.74±6.23	18.99±1.17	-0.933	0.00720	0.411	0.22885	-0.327	0.33853	0.33853	pubmed
ENSMUSG00000057123	Gja5	18.06±3.37	8.5±1.57	13.57±1.99	12.02±1.53	-1.084	0.04579	-0.409	1.00000	-0.586	0.24529	0.24529	pubmed
ENSMUSG00000057706	Mex3b	39.92±5.1	14.91±3.84	47.12±9.75	19.85±1.48	-1.422	0.00039	0.237	1.00000	-1.011	0.00864	0.00864	pubmed
ENSMUSG00000057777	Mab21l2	93.9±34.13	49.74±2.63	136.67±23.84	68.04±14.81	-0.918	0.04692	0.540	0.32358	-0.464	0.32191	0.32191	pubmed
ENSMUSG00000058881	Zfp516	185.04±11.53	95.5±10.66	193.85±12.35	120.25±3.6	-0.954	0.00003	0.067	0.88543	-0.622	0.00753	0.00753	pubmed
ENSMUSG00000060044	Tmem26	16.18±6.76	5.33±0.81	11.94±4.1	4.75±1.21	-1.602	0.00886	-0.433	1.00000	-1.766	0.00193	0.00193	pubmed
ENSMUSG00000060183	Cxcl11	12.65±1.61	3.19±0.65	14.49±1.96	5.01±0.25	-1.985	0.00278	0.197	1.00000	-1.336	0.01909	0.01909	pubmed
ENSMUSG00000061411	Nol4l	997.67±95.76	563.66±39.94	935.32±58.16	492.39±36.72	-0.824	0.00000	-0.093	0.70618	-1.019	0.00000	0.00000	pubmed
ENSMUSG00000061654	Spry3	40.91±9.17	14.62±1.87	48.48±11.98	13.9±5.57	-1.483	0.00767	0.245	1.00000	-1.561	0.00244	0.00244	pubmed
ENSMUSG00000062157	Ifnlr1	178.56±14.23	92.59±3.03	159.35±17.91	82.84±23.2	-0.948	0.00070	-0.164	0.73078	-1.112	0.00002	0.00002	pubmed
ENSMUSG00000062488	Ifit3b	19.49±3.46	8.23±1.92	18.85±7.08	10.23±2.96	-1.239	0.01380	-0.047	0.96227	-0.924	0.04404	0.04404	pubmed
ENSMUSG00000062545	Tlr12	72.45±7.72	38.07±6.23	63.53±3.8	32.29±4.74	-0.928	0.00310	-0.189	0.69947	-1.165	0.00006	0.00006	pubmed
ENSMUSG00000062960	Kdr	230.46±4.65	120.67±15.06	255.99±25.36	133.57±6.98	-0.933	0.00000	0.151	0.51046	-0.786	0.00000	0.00000	pubmed
ENSMUSG00000063060	Sox7	15.46±3.53	6.4±1.36	17.46±0.97	8.91±2.7	-1.274	0.01743	0.176	1.00000	-0.782	0.10669	0.10669	pubmed
ENSMUSG00000063146	Clip2	810.32±18.87	349.15±10.33	742.12±44.67	281.61±14.85	-1.215	0.00000	-0.127	0.39567	-1.524	0.00000	0.00000	pubmed
ENSMUSG00000063727	Tnfrsf11b	47.44±5.86	17.54±3.49	54.31±4.06	21.71±1.88	-1.435	0.00000	0.195	0.66608	-1.125	0.00012	0.00012	pubmed
ENSMUSG00000068893	Sprr2a2	143.61±18.53	74.8±13.09	177.96±11.64	54.26±15.92	-0.941	0.00445	0.310	0.50979	-1.408	0.00000	0.00000	pubmed
ENSMUSG00000070304	Scn2b	52.36±3.99	30.05±5.13	58.96±3.21	35.85±5.29	-0.801	0.02037	0.170	1.00000	-0.548	0.09713	0.09713	pubmed
ENSMUSG00000070315	4930581F22Rik	37.04±6.48	11.97±2.3	37.07±7.55	14.67±5.87	-1.628	0.00000	0.004	0.99799	-1.346	0.00001	0.00001	pubmed
ENSMUSG00000071226	Cecr2	23.32±13.59	8.79±1.78	18.54±13.37	15.05±1.16	-1.409	0.04319	-0.327	0.74471	-0.634	0.35765	0.35765	pubmed
ENSMUSG00000072618	Gm10384	29.26±0.54	10.63±2.65	25.85±2.12	11.11±2.31	-1.460	0.00174	-0.179	1.00000	-1.403	0.00130	0.00130	pubmed
ENSMUSG00000072844	G530011O06Rik	21.46±8.99	7.96±2.55	19.49±0.5	10.03±2.39	-1.426	0.02306	-0.136	1.00000	-1.095	0.05705	0.05705	pubmed
ENSMUSG00000073002	Vamp5	36.95±1.98	20.27±6.67	33.49±7.19	15.85±4.96	-0.868	0.03028	-0.141	0.82457	-1.225	0.00058	0.00058	pubmed
ENSMUSG00000073403	Gm10499	263.15±62.04	142.23±14.99	220.8±54.78	104.77±26.07	-0.888	0.02289	-0.253	0.66608	-1.331	0.00008	0.00008	pubmed
ENSMUSG00000073802	Cdkn2b	903.23±40.04	181.48±12.04	965.05±80.58	146.91±15.42	-2.315	0.00000	0.095	0.68823	-2.623	0.00000	0.00000	pubmed
ENSMUSG00000074063	Osgin1	315.39±46.25	144.4±10	215.68±21.11	97.19±21.33	-1.127	0.00058	-0.549	0.19903	-1.699	0.00000	0.00000	pubmed
ENSMUSG00000074896	Ifit3	62.17±7.41	27.98±5.26	64.51±5.99	33.24±4.79	-1.152	0.00001	0.053	0.91471	-0.900	0.00021	0.00021	pubmed
ENSMUSG00000074934	Grem1	1003.38±143.45	486.73±63.07	1150.13±213.13	610.57±111.7	-1.044	0.00003	0.197	0.64136	-0.716	0.00508	0.00508	pubmed
ENSMUSG00000075389	2810410L24Rik	33.67±1.97	15.68±2.15	36.28±1.15	17.08±1.89	-1.102	0.00494	0.107	1.00000	-0.982	0.00718	0.00718	pubmed
ENSMUSG00000076580	Igkv8-27	1635.43±850.16	251.43±71.07	910.61±842.33	750.89±266.01	-2.701	0.01496	-0.845	0.61175	-1.123	0.35870	0.35870	pubmed
ENSMUSG00000078202	Nrarp	355.65±54.54	134.24±17.25	325.84±12.47	151.37±10.73	-1.405	0.00000	-0.126	0.70997	-1.232	0.00000	0.00000	pubmed
ENSMUSG00000078771	Evi2a	66.15±16.44	30.88±2.34	59.67±7.77	22.54±0.35	-1.099	0.00140	-0.147	0.80465	-1.553	0.00000	0.00000	pubmed
ENSMUSG00000079363	Gbp4	149.11±19.28	73.77±5.82	93.98±24.03	50.71±3.15	-1.015	0.00004	-0.664	0.01752	-1.554	0.00000	0.00000	pubmed
ENSMUSG00000083307	AA414768	18.21±3.13	7.47±2.05	23.34±3.6	13.21±2.76	-1.289	0.03697	0.357	1.00000	-0.461	0.45737	0.45737	pubmed
ENSMUSG00000087006	Gm13889	62.18±11.21	23.44±1.86	65.98±16.39	32.13±5.07	-1.408	0.00004	0.086	1.00000	-0.949	0.00483	0.00483	pubmed

Ensgene	Symbol	HP	HD	ZP	ZD	LFC HD-HP	FDR HD-HP	LFC ZP-HP	FDR ZP-HP	LFC ZD-HP	FDR ZD-HP	Ratio	Links
ENSMUSG00000087611	4930458D05Rik	35.5±5.95	15.7±0.7	39.11±8.81	15.58±1.62	-1.178	0.01148	0.138	1.00000	-1.191	0.00548	0.00548	pubmed
ENSMUSG00000089762	Ier5l	24.63±3.02	10.12±2.12	16.99±1.34	9.98±1	-1.283	0.00507	-0.534	1.00000	-1.298	0.00220	0.00220	pubmed
ENSMUSG00000090877	Hspa1b	89.39±48.37	43.42±16.3	50.68±6.34	25.25±6.98	-1.043	0.04541	-0.820	0.15821	-1.828	0.00003	0.00003	pubmed
ENSMUSG00000094584	Ms4a18	553.64±100.71	289.6±28.71	544.52±31.88	311.97±7.88	-0.935	0.00000	-0.024	0.96048	-0.828	0.00001	0.00001	pubmed
ENSMUSG00000095589	Ighv1-55	806.42±229.21	181.85±52.19	487.01±355.18	457.76±63.74	-2.149	0.00601	-0.728	0.52549	-0.817	0.35663	0.35663	pubmed
ENSMUSG00000095704	Gm8221	32.51±2.82	9.05±0.81	25.34±2.25	3.47±2.49	-1.845	0.00030	-0.359	1.00000	-3.217	0.00000	0.00000	pubmed
ENSMUSG00000096632	Igkv9-124	54.9±7.01	10.73±9.29	12.7±2.91	24.63±10.36	-2.358	0.00288	-2.112	1.00000	-1.154	0.15853	0.15853	pubmed
ENSMUSG00000097343	9030407P20Rik	25.02±3.09	9.6±2.46	18.85±3.57	11.75±7.37	-1.383	0.02220	-0.410	1.00000	-1.077	0.05353	0.05353	pubmed
ENSMUSG00000099032	Tcf24	13.56±2.09	5.59±2.96	12.09±3.31	10.04±0.58	-1.280	0.03421	-0.172	0.84429	-0.437	0.45389	0.45389	pubmed
ENSMUSG00000103529	A730089K16Rik	41.26±3.13	19.66±5.8	34.56±6.61	23.94±3.88	-1.068	0.01908	-0.256	1.00000	-0.788	0.06695	0.06695	pubmed

Appendix Table S14

List of genes derived from the RNASEQ experiment shown in Fig. 2. Definitions of the categories see Supplemental Information Appendix Fig. S1.

Ensgene	Symbol	HP	HD	ZP	ZD	LFC HD-HP	FDR HD-HP	LFC ZP-HP	FDR ZP-HP	LFC ZD-HP	FDR ZD-HP	Ratio	Links
ENSMUSG00000020407	Upp1	3408.47±375	3325.29±343.09	629.96±59.01	443.34±48.28	-0.036	0.93962	-2.436	0.00000	-2.942	0.00000	0.00000	pubmed
ENSMUSG00000021509	Slc25a48	1103.71±259.5	897.42±59.41	621.14±110.29	265.13±94.15	-0.299	0.58523	-0.829	0.03521	-2.059	0.00000	0.00000	pubmed
ENSMUSG00000037321	Tap1	866.34±176.71	645.27±119.45	411.18±45.48	257.53±47.61	-0.425	0.13992	-1.075	0.00000	-1.749	0.00000	0.00000	pubmed
ENSMUSG00000044827	Tlr1	155.67±31.73	123.36±18.21	79.02±23.87	43.08±0.94	-0.336	0.50022	-0.977	0.00668	-1.853	0.00000	0.00000	pubmed
ENSMUSG00000057346	Apol9a	71.71±20.35	98.56±19.27	38.44±12	19.23±6.82	0.460	0.30002	-0.900	0.01841	-1.904	0.00000	0.00000	pubmed
ENSMUSG00000068452	Duox2	3883.6±310.71	3337.87±118.55	779.02±79.53	416.45±160.31	-0.218	0.65497	-2.318	0.00000	-3.222	0.00000	0.00000	pubmed
ENSMUSG00000079362	Gbp6	340.01±67.38	231.82±35.15	119.13±16.18	71.29±17.21	-0.552	0.07049	-1.513	0.00000	-2.250	0.00000	0.00000	pubmed

Appendix Table S15

List of genes derived from the RNASEQ experiment shown in Fig. 2. Definitions of the categories see Supplemental Information Appendix Fig. S1.

Ensgene	Symbol	HP	HD	ZP	ZD	LFC HD-HP	FDR HD-HP	LFC ZP-HP	FDR ZP-HP	LFC ZD-HP	FDR ZD-HP	Ratio	Links
ENSMUSG00000000805	Car4	1141.2±516.06	1045.69±112.26	388.31±80.89	299.94±82.77	-0.126	0.91372	-1.555	0.00393	-1.928	0.00004	0.00004	pubmed
ENSMUSG00000002033	Cd3g	142.41±33.5	93.63±10.68	78.1±18.5	47.86±5.93	-0.604	0.12729	-0.865	0.01717	-1.571	0.00000	0.00000	pubmed
ENSMUSG00000002992	Apoc2	228.87±3.92	264.61±51.63	126.18±38.13	153.03±13.13	0.210	0.55122	-0.861	0.00021	-0.580	0.01106	0.01106	pubmed
ENSMUSG00000005947	Itgae	197.23±31.43	263.32±55.01	108.39±4.68	150±4.31	0.418	0.18952	-0.863	0.00127	-0.394	0.15870	0.15870	pubmed
ENSMUSG00000013653	1810065E05Rik	707.06±198.62	690.47±162.87	264.89±83.67	207.78±38.09	-0.034	0.97874	-1.416	0.00026	-1.767	0.00000	0.00000	pubmed
ENSMUSG00000015437	Gzmb	381.85±107.82	377.77±101.74	79.21±17.87	103.15±26.74	-0.015	0.99037	-2.270	0.00000	-1.887	0.00000	0.00000	pubmed
ENSMUSG00000019872	Smpdl3a	1719.56±147.45	1817.23±117.44	919.72±69.82	911.42±114.32	0.080	0.73485	-0.903	0.00000	-0.916	0.00000	0.00000	pubmed
ENSMUSG00000020407	Upp1	3408.47±375	3325.29±343.09	629.96±59.01	443.34±48.28	-0.036	0.93962	-2.436	0.00000	-2.942	0.00000	0.00000	pubmed
ENSMUSG00000020826	Nos2	3155.72±477.65	2381.6±298.56	906.43±220.92	525.93±190.25	-0.406	0.61358	-1.800	0.00038	-2.585	0.00000	0.00000	pubmed
ENSMUSG00000021850	1700011H14Rik	310.72±14.06	411.93±39.83	163.5±14.45	214.7±27.41	0.407	0.03833	-0.926	0.00000	-0.534	0.00220	0.00220	pubmed
ENSMUSG00000022847	Thpo	34.69±9.8	52.1±9.92	14.4±4.73	23.47±4.51	0.584	0.08501	-1.265	0.00023	-0.574	0.08850	0.08850	pubmed
ENSMUSG00000022938	Fam3b	869.14±69.11	1150.58±30.64	497.04±33.16	607.94±171.21	0.405	0.21448	-0.806	0.00272	-0.516	0.04941	0.04941	pubmed
ENSMUSG00000023132	Gzma	868.1±162.68	1230.39±370.51	241.77±58.85	346.65±66.91	0.503	0.25816	-1.845	0.00000	-1.324	0.00002	0.00002	pubmed
ENSMUSG00000023176	Cpn2	4.71±3.7	6.63±3.71	0.23±0.32	2.91±2.33	0.506	0.55008	-4.369	0.03593	-0.641	0.42105	0.42105	pubmed
ENSMUSG00000023272	Creld2	547.95±101.62	522.45±86.5	285.64±22.74	329.21±10.04	-0.069	0.94984	-0.940	0.04802	-0.735	0.08968	0.08968	pubmed
ENSMUSG00000024338	Psmb8	1302.56±164.76	1290.13±162.82	599.02±32.78	562.12±74.36	-0.014	0.98274	-1.120	0.00000	-1.212	0.00000	0.00000	pubmed
ENSMUSG00000024353	Mzb1	341.34±78.75	275.96±24.56	186.67±60.38	123.64±18.49	-0.307	0.55855	-0.870	0.02028	-1.464	0.00000	0.00000	pubmed
ENSMUSG00000024610	Cd74	15707.85±2737.99	17491.4±1412.96	7445.84±1309.47	8139.57±1743.44	0.155	0.71071	-1.077	0.00000	-0.948	0.00002	0.00002	pubmed
ENSMUSG00000024863	Mbl2	13.15±2.08	11.71±1.64	2.04±0.58	2.9±2.49	-0.163	0.82170	-2.684	0.00002	-2.126	0.00007	0.00007	pubmed
ENSMUSG00000024887	Asah2	1024.61±276.01	1255.17±403.72	479.78±202.39	363.67±111.5	0.293	0.61956	-1.095	0.00526	-1.494	0.00001	0.00001	pubmed
ENSMUSG00000025163	Cd7	156.05±14	181.26±41.31	72.36±7.49	79.14±3.79	0.217	0.58816	-1.109	0.00001	-0.979	0.00005	0.00005	pubmed
ENSMUSG00000026117	Zap70	63.47±19.94	63.1±2.96	34.28±8.9	34.13±6.03	-0.007	0.99665	-0.885	0.01896	-0.891	0.00836	0.00836	pubmed
ENSMUSG00000026880	Stom	2986.99±792.43	2623.68±46.86	1251.77±125.8	946.01±236.23	-0.187	0.65761	-1.255	0.00000	-1.659	0.00000	0.00000	pubmed
ENSMUSG00000027514	Zbp1	581.74±123.71	585.19±48.4	117.19±14.21	104.85±21.06	0.009	0.99337	-2.311	0.00000	-2.470	0.00000	0.00000	pubmed
ENSMUSG00000028028	Alpk1	248.23±21.35	222.94±27.32	114.34±13.5	104.33±12.19	-0.155	0.66208	-1.118	0.00000	-1.252	0.00000	0.00000	pubmed
ENSMUSG00000028270	Gbp2	535.29±130.31	407.96±2.96	277.39±48.63	325.46±92.08	-0.392	0.30901	-0.948	0.00131	-0.717	0.01104	0.01104	pubmed
ENSMUSG00000028737	Aldh4a1	151.22±26.51	257.22±38.71	70.45±14.83	64.89±6.96	0.766	0.00016	-1.104	0.00000	-1.223	0.00000	0.00000	pubmed
ENSMUSG00000028864	Hgf	62.59±16.75	36.4±6.28	33.31±0.97	31.34±5.24	-0.780	0.00511	-0.909	0.00098	-0.991	0.00010	0.00010	pubmed
ENSMUSG00000028885	Smpdl3b	444.41±68.65	375.98±16.88	127.88±10.28	88.8±32.16	-0.241	0.60871	-1.797	0.00000	-2.326	0.00000	0.00000	pubmed
ENSMUSG00000029084	Cd38	2460.28±297.53	1944.83±163.55	1075.49±93.8	836.27±107	-0.339	0.05980	-1.194	0.00000	-1.557	0.00000	0.00000	pubmed
ENSMUSG00000029417	Cxcl9	178.21±67.99	151.32±21.78	56.44±0.18	72.39±9.07	-0.235	0.69254	-1.658	0.00000	-1.298	0.00009	0.00009	pubmed
ENSMUSG00000029530	Ccr9	130.39±17.61	114.34±30.63	70.09±9.69	73.59±7.92	-0.189	0.73485	-0.895	0.01000	-0.824	0.00904	0.00904	pubmed
ENSMUSG00000029657	Hsph1	781.37±303.46	699.44±259.18	370.5±22.75	539.82±116.22	-0.160	0.86490	-1.077	0.03766	-0.534	0.30516	0.30516	pubmed
ENSMUSG00000029798	Herc6	245.95±34.54	250.39±11.78	89.95±1.67	68.83±7.24	0.027	0.95579	-1.450	0.00000	-1.833	0.00000	0.00000	pubmed
ENSMUSG00000030017	Reg3g	75905.19±24575.39	74127.64±2742.46	8753.08±1914.38	10531.95±3537.07	-0.034	0.99409	-3.116	0.01088	-2.849	0.01007	0.01007	pubmed
ENSMUSG00000030786	Itgam	374.71±2.1	379.53±39.08	163.52±7.21	179.48±26.63	0.019	0.97499	-1.196	0.00000	-1.060	0.00000	0.00000	pubmed
ENSMUSG00000031410	Nxf7	93.5±7.03	139.83±14.71	47.55±2.52	52.55±11.09	0.580	0.04148	-0.975	0.00040	-0.834	0.00134	0.00134	pubmed
ENSMUSG00000031551	Ido1	470.09±72.24	661.52±113.27	195.8±14.69	272.05±35.61	0.493	0.06524	-1.264	0.00000	-0.788	0.00041	0.00041	pubmed
ENSMUSG00000031584	Gsr	2682.05±559.99	2409.56±91.4	1479.69±169.67	1673.15±134.36	-0.155	0.54516	-0.858	0.00000	-0.681	0.00001	0.00001	pubmed
ENSMUSG00000032093	Cd3e	100.89±23.04	87.22±8.14	57.33±11.42	36.69±3.74	-0.209	0.71396	-0.814	0.03155	-1.457	0.00000	0.00000	pubmed
ENSMUSG00000033213	AA467197	701.93±61.22	756.62±37.63	340.12±6.91	283.86±89.14	0.108	0.83622	-1.045	0.00007	-1.307	0.00000	0.00000	pubmed
ENSMUSG00000034438	Gbp8	155.76±11.57	147.08±20.01	76.55±13.55	63.34±13.08	-0.083	0.87491	-1.023	0.00006	-1.299	0.00000	0.00000	pubmed
ENSMUSG00000035042	Ccl5	289.89±69.65	352.13±111.08	131.69±19.91	122.08±18.05	0.281	0.60998	-1.139	0.00165	-1.248	0.00012	0.00012	pubmed
ENSMUSG00000035186	Ubd	406.04±138.7	439.62±156.59	44.07±26.18	19.05±6.55	0.115	0.94946	-3.202	0.00000	-4.417	0.00000	0.00000	pubmed
ENSMUSG00000036594	H2-Aa	7573.16±1230.06	8243.44±987.69	3210.28±691.17	3216.21±763.04	0.122	0.80591	-1.238	0.00000	-1.236	0.00000	0.00000	pubmed
ENSMUSG00000037095	Lrg1	76.05±34.2	79.29±13.21	32.46±7.97	53.59±3.53	0.062	0.96033	-1.225	0.01302	-0.504	0.32570	0.32570	pubmed
ENSMUSG00000037145	2210407C18Rik	643.49±77.62	976.74±352.42	253.52±45.06	352.35±28.21	0.602	0.12870	-1.344	0.00003	-0.869	0.00730	0.00730	pubmed
ENSMUSG00000037321	Tap1	866.34±176.71	645.27±119.45	411.18±45.48	257.53±47.61	-0.425	0.13992	-1.075	0.00000	-1.749	0.00000	0.00000	pubmed
ENSMUSG00000037649	H2-DMa	741.51±116.46	735.38±76.28	353.77±65.79	331.12±17.09	-0.012	0.98776	-1.067	0.00000	-1.163	0.00000	0.00000	pubmed
ENSMUSG00000038751	Ptk6	681.24±127.01	656.35±18.98	358.92±25.06	253.99±86.18	-0.054	0.95469	-0.925	0.01454	-1.424	0.00001	0.00001	pubmed
ENSMUSG00000040253	Gbp7	318.12±48.74	214.85±23.52	177.69±22.96	139.63±21.49	-0.566	0.00344	-0.840	0.00000	-1.185	0.00000	0.00000	pubmed
ENSMUSG00000041193	Pla2g5	213.02±45.98	242.58±15.49	47.58±5.69	62.83±8.19	0.188	0.65988	-2.162	0.00000	-1.759	0.00000	0.00000	pubmed
ENSMUSG00000041481	Serpina3g	201.59±89.11	182.76±25.95	91.77±31.07	144.25±22.63	-0.141	0.86031	-1.134	0.00755	-0.482	0.27791	0.27791	pubmed
ENSMUSG00000042808	Gpx2	4728.82±871	4821.61±265.24	2047.79±240.54	2559.25±124.46	0.028	0.95886	-1.207	0.00000	-0.886	0.00000	0.00000	pubmed
ENSMUSG00000043592	Unc5cl	443.59±80.62	469±18.12	171.45±6.12	192.3±4.82	0.081	0.82418	-1.371	0.00000	-1.206	0.00000	0.00000	pubmed
ENSMUSG00000044165	Bcl2l15	1103.66±200.26	1388.02±31.74	595.13±87.18	741.12±111.84	0.331	0.27662	-0.891	0.00009	-0.575	0.01129	0.01129	pubmed
ENSMUSG00000046688	Tifa	1113.79±234.52	688.15±128.15	354.61±36.19	299.7±18.59	-0.695	0.00726	-1.651	0.00000	-1.894	0.00000	0.00000	pubmed
ENSMUSG00000047517	Dmbt1	18537.56±7356.43	26403.13±3068.26	4775.3±1094.22	8228.9±657.22	0.510	0.44172	-1.957	0.00001	-1.172	0.00925	0.00925	pubmed
ENSMUSG00000049608	Gpr55	191.35±7.38	164.87±39.76	104.57±18.2	104.62±3.64	-0.215	0.61037	-0.871	0.00165	-0.871	0.00057	0.00057	pubmed
ENSMUSG00000053977	Cd8a	189.32±24.18	239.79±52.74	85.21±13.08	88.87±13.33	0.341	0.38620	-1.151	0.00005	-1.089	0.00005	0.00005	pubmed
ENSMUSG00000054169	Ceacam10	320.44±32.78	357.87±104.08	164.7±34.82	176.99±14.06	0.159	0.77815	-0.960	0.00331	-0.856	0.00445	0.00445	pubmed
ENSMUSG00000055312	Them7	6.59±1.85	5.85±3.32	2.28±0.68	2.5±0.71	-0.164	0.88528	-1.532	0.04914	-1.388	0.04363	0.04363	pubmed
ENSMUSG00000055413	H2-Q5	54.42±8.98	34.09±2.98	25.83±10.64	19.14±4.12	-0.674	0.17371	-1.070	0.01552	-1.510	0.00010	0.00010	pubmed
ENSMUSG00000055415	Atp10b	2042.16±242.02	2075.76±105.44	1105.93±69.78	1308.86±89.94	0.024	0.95235	-0.885	0.00000	-0.642	0.00000	0.00000	pubmed
ENSMUSG00000055978	Fut2	145.93±44.14	223.77±23.27	24.86±5.87	34.92±16.44	0.617	0.28807	-2.552	0.00000	-2.068	0.00000	0.00000	pubmed
ENSMUSG00000056737	Capg	296.32±61.78	252.03±2.93	159.81±49.64	107.69±18.95	-0.233	0.64658	-0.890	0.00823	-1.460	0.00000	0.00000	pubmed
ENSMUSG00000057465	Saa2	6.82±1.55	8.25±1.37	0.46±0.65	0.49±0.35	0.277	0.89677	-3.906	0.02608	-3.771	0.01543	0.01543	pubmed
ENSMUSG00000058624	Gda	5659.12±494.73	7203.94±879.55	2674.6±352.3	2308.3±616.67	0.348	0.23436	-1.081	0.00000	-1.294	0.00000	0.00000	pubmed
ENSMUSG00000060586	H2-Eb1	4261.77±970.81	4243.18±337.22	2253.66±491.77	2201.21±225.48	-0.006	0.99622	-0.919	0.00053	-0.953	0.00008	0.00008	pubmed
ENSMUSG00000060950	Trmt61a	64.95±5.8	48.43±1.9	34.94±7.24	38.07±7.78	-0.424	0.26456	-0.896	0.00386	-0.767	0.00765	0.00765	pubmed
ENSMUSG00000061762	Tac1	170.63±34.71	146.09±5.71	82.2±6.51	78.87±11.3	-0.223	0.58015	-1.054	0.00004	-1.113	0.00000	0.00000	pubmed

Ensgene	Symbol	HP	HD	ZP	ZD	LFC HD-HP	FDR HD-HP	LFC ZP-HP	FDR ZP-HP	LFC ZD-HP	FDR ZD-HP	Ratio	Links
ENSMUSG00000061947	Serpina10	19.26±6.57	13.01±2.9	5.35±4.23	3.03±1.31	-0.559	0.18650	-1.820	0.00001	-2.675	0.00000	0.00000	pubmed
ENSMUSG00000063206	Gm15315	930.72±141.35	1045.24±234.48	414.86±101.64	651.99±180.19	0.167	0.80240	-1.166	0.00137	-0.513	0.18382	0.18382	pubmed
ENSMUSG00000063354	Slc39a4	1717.2±69.61	2216.37±134.48	219.28±26.79	191.4±38.72	0.368	0.08876	-2.969	0.00000	-3.168	0.00000	0.00000	pubmed
ENSMUSG00000063388	BC023105	4.93±0.65	2.67±1.36	0.92±0.65	1.46±0.98	-0.885	0.37607	-2.438	0.02951	-1.712	0.05018	0.05018	pubmed
ENSMUSG00000068227	Il2rb	98.6±36.31	82.31±9.25	39.78±15.22	44.28±8.72	-0.260	0.69158	-1.306	0.00142	-1.152	0.00245	0.00245	pubmed
ENSMUSG00000069792	Wfdc17	29.98±2.54	22.93±4.16	15.24±2.39	12.83±1.52	-0.388	0.32077	-0.980	0.00221	-1.229	0.00005	0.00005	pubmed
ENSMUSG00000070348	Ccnd1	1586.11±197.49	961.04±11.25	747.62±44.8	699.98±81.37	-0.723	0.02675	-1.085	0.00033	-1.180	0.00002	0.00002	pubmed
ENSMUSG00000070427	Il18bp	210.31±41.49	256.85±11.02	47.59±10.63	51.16±4.75	0.289	0.37015	-2.140	0.00000	-2.037	0.00000	0.00000	pubmed
ENSMUSG00000072620	Slfn2	215.25±77.57	166.09±30.17	107.18±51.27	112.89±5.29	-0.374	0.51871	-1.004	0.01787	-0.931	0.01458	0.01458	pubmed
ENSMUSG00000073421	H2-Ab1	6155.39±893.8	6272.1±918.93	2963.28±486.3	2960.33±628.38	0.027	0.97182	-1.055	0.00001	-1.056	0.00000	0.00000	pubmed
ENSMUSG00000073555	Gm4951	35.26±8.11	23.98±4.47	19.99±5.29	32.43±5.23	-0.554	0.12891	-0.818	0.01399	-0.114	0.78409	0.78409	pubmed
ENSMUSG00000074115	Saa1	2308.41±726.19	2635.2±420.41	60.96±34.19	130.74±38.34	0.191	0.93688	-5.243	0.00000	-4.142	0.00000	0.00000	pubmed
ENSMUSG00000074151	Nlrc5	236.19±42.88	244.15±23.22	92.87±24.05	80.87±12.92	0.048	0.93658	-1.344	0.00000	-1.543	0.00000	0.00000	pubmed
ENSMUSG00000074768	Bhmt	55±37.85	43.73±39.45	7.55±3.23	21.06±11.7	-0.330	0.84524	-2.867	0.00191	-1.381	0.13231	0.13231	pubmed
ENSMUSG00000074892	B3galt5	145.36±82.41	240.62±53.78	20.12±2.58	22.37±1.84	0.728	0.30852	-2.852	0.00000	-2.701	0.00000	0.00000	pubmed
ENSMUSG00000076514	Igkv17-121	176.6±102.24	200.97±70.58	41.52±11.54	71.81±24.34	0.187	0.89092	-2.088	0.00164	-1.299	0.04551	0.04551	pubmed
ENSMUSG00000076934	Iglv1	625.49±172.94	681.57±282.94	201.88±76.44	206.77±38.43	0.124	0.93046	-1.631	0.01059	-1.597	0.00516	0.00516	pubmed
ENSMUSG00000078853	Igtp	566.62±179.19	357.25±36.38	161.54±14.69	110.39±29.71	-0.665	0.03931	-1.810	0.00000	-2.357	0.00000	0.00000	pubmed
ENSMUSG00000078920	Ifi47	641.49±125.77	650.2±77.24	311.53±29.08	286.56±18.1	0.020	0.98075	-1.042	0.00001	-1.162	0.00000	0.00000	pubmed
ENSMUSG00000078921	Tgtp2	46.23±6.02	44.68±8.28	25.29±4.46	34.7±2.69	-0.047	0.95270	-0.866	0.01059	-0.410	0.22338	0.22338	pubmed
ENSMUSG00000079180	Mptx2	5424.81±1186.22	6362.99±1347.42	1841.32±332.23	3046.11±1235.13	0.230	0.86742	-1.559	0.04016	-0.833	0.26694	0.26694	pubmed
ENSMUSG00000079362	Gbp6	340.01±67.38	231.82±35.15	119.13±16.18	71.29±17.21	-0.552	0.07049	-1.513	0.00000	-2.250	0.00000	0.00000	pubmed
ENSMUSG00000079445	B3gnt7	740.06±189.24	552.68±67.5	148.44±19.23	167.52±14.82	-0.421	0.22302	-2.318	0.00000	-2.143	0.00000	0.00000	pubmed
ENSMUSG00000079507	H2-Q2	427.12±36.68	514.3±14.74	233.56±68.39	200.42±67.66	0.268	0.61175	-0.871	0.01740	-1.093	0.00049	0.00049	pubmed
ENSMUSG00000079547	H2-DMb1	620.24±35.1	643.16±115.65	244.3±11.22	260.9±62.83	0.053	0.92894	-1.344	0.00000	-1.250	0.00000	0.00000	pubmed
ENSMUSG00000082292	Gm12250	133.56±33.84	144.52±8.9	33.85±3.43	37.6±7.73	0.115	0.83270	-1.981	0.00000	-1.824	0.00000	0.00000	pubmed
ENSMUSG00000090942	F830016B08Rik	14.56±2.57	10.38±0.1	5.41±1.33	9.17±3.15	-0.485	0.42464	-1.418	0.00566	-0.652	0.16290	0.16290	pubmed
ENSMUSG00000093896	Ighv1-76	265.63±172.96	180.02±87.9	58.21±39.99	78.77±9.95	-0.561	0.72369	-2.191	0.03506	-1.753	0.06398	0.06398	pubmed
ENSMUSG00000094689	Ighv1-81	276.54±139.58	160.91±52.52	33.98±7.18	65.98±47.56	-0.781	0.54348	-3.025	0.00037	-2.069	0.01246	0.01246	pubmed
ENSMUSG00000094694	Ighv1-9	978.4±69.18	267.04±120.67	194.48±120.15	207.16±138.4	-1.873	0.11983	-2.331	0.04373	-2.240	0.02690	0.02690	pubmed
ENSMUSG00000095794	Igkv6-17	294.79±92.93	160.5±67.72	98.78±51.04	86.79±21.14	-0.877	0.27312	-1.578	0.01832	-1.763	0.00249	0.00249	pubmed
ENSMUSG00000096727	Psmb9	565.73±60.14	466.18±73.29	250.78±16.17	228.04±42.14	-0.279	0.32817	-1.173	0.00000	-1.309	0.00000	0.00000	pubmed

Appendix Table S16

List of genes derived from the RNASEQ experiment shown in Fig. 2. Definitions of the categories see Supplemental Information Appendix Fig. S1.

Ensgene	Symbol	HP	HD	ZP	ZD	LFC HD-HP	FDR HD-HP	LFC ZP-HP	FDR ZP-HP	LFC ZD-HP	FDR ZD-HP	Ratio	Links
ENSMUSG00000000409	Lck	172.38±76.44	104.44±13.31	103.28±51.91	45.39±10.77	-0.723	0.26261	-0.738	0.25536	-1.926	0.00002	0.00002	pubmed
ENSMUSG00000005107	Slc2a9	257.83±29.94	311.72±11.31	309.41±13.5	207.22±55.38	0.274	0.32229	0.263	0.35387	-0.318	0.15660	0.15660	pubmed
ENSMUSG00000009772	Nuak2	987.96±145.96	727.03±66.42	821.77±117.57	365.74±73.34	-0.442	0.16102	-0.266	0.48293	-1.434	0.00000	0.00000	pubmed
ENSMUSG00000013707	Tnfaip8l2	134.98±11.7	81.46±12.77	109.72±13.9	47.13±8.44	-0.729	0.00678	-0.298	0.41208	-1.521	0.00000	0.00000	pubmed
ENSMUSG00000017830	Dhx58	64.11±11.07	58.56±3.33	55.97±4.51	36.9±3.48	-0.129	0.75493	-0.194	0.58302	-0.797	0.00051	0.00051	pubmed
ENSMUSG00000017868	Sgk2	428.44±36.27	345.12±30.05	350.67±86.71	118.71±87.77	-0.312	0.73418	-0.289	0.75042	-1.854	0.00014	0.00014	pubmed
ENSMUSG00000018401	Mtmr4	948.75±94.67	727.43±41.46	802.28±127.39	495.58±64.84	-0.383	0.11826	-0.242	0.40835	-0.938	0.00000	0.00000	pubmed
ENSMUSG00000018648	Dusp14	25.48±2.96	19.95±1.61	24.44±8.07	9.67±3.82	-0.353	0.54796	-0.060	0.94269	-1.414	0.00048	0.00048	pubmed
ENSMUSG00000019189	Rnf145	779.26±25.44	492.48±70.07	519.24±51.48	343.18±30.97	-0.662	0.00023	-0.585	0.00260	-1.183	0.00000	0.00000	pubmed
ENSMUSG00000019823	Mical1	583.18±66.34	370.96±47.8	411.14±60.93	224.37±23.61	-0.653	0.00243	-0.504	0.03791	-1.378	0.00000	0.00000	pubmed
ENSMUSG00000019888	Mgat4c	4213.85±584.27	2976.37±352.02	3529.3±351.91	1931.86±400.61	-0.502	0.06906	-0.256	0.46941	-1.125	0.00000	0.00000	pubmed
ENSMUSG00000020062	Slc5a8	3960.66±475.24	2607.61±127.09	2692.33±109.93	1456.38±470.47	-0.603	0.07454	-0.557	0.12265	-1.444	0.00000	0.00000	pubmed
ENSMUSG00000020641	Rsad2	75.32±2.32	95.04±11.77	76.9±20.25	43.58±7.11	0.335	0.38886	0.029	0.96701	-0.793	0.00543	0.00543	pubmed
ENSMUSG00000020733	Slc9a3r1	6804.71±445.85	6403.65±191.78	6886.56±408.19	4374.24±1271.9	-0.088	0.82770	0.017	0.97616	-0.638	0.00121	0.00121	pubmed
ENSMUSG00000021280	Exoc3l4	1099.97±89.82	759.46±35.68	802.18±212.71	357.5±136.74	-0.534	0.25816	-0.456	0.36518	-1.622	0.00000	0.00000	pubmed
ENSMUSG00000021798	Ldb3	652.23±8.4	449.24±9.58	554.13±28.27	240.43±42.94	-0.538	0.00162	-0.235	0.30977	-1.442	0.00000	0.00000	pubmed
ENSMUSG00000022906	Parp9	1129.2±87.24	838.6±61.63	890.68±57.61	454.25±22.34	-0.429	0.00003	-0.342	0.00253	-1.314	0.00000	0.00000	pubmed
ENSMUSG00000023959	Clic5	3415.45±291.38	2606.34±13.98	3225.84±279.56	1568.63±329.47	-0.390	0.12647	-0.082	0.83836	-1.123	0.00000	0.00000	pubmed
ENSMUSG00000024105	Themis3	1014.64±48.05	647.16±29.38	915.03±62.01	315.52±70.19	-0.649	0.00196	-0.149	0.66081	-1.686	0.00000	0.00000	pubmed
ENSMUSG00000024313	Mep1b	14009.96±685.37	12884.17±493.26	9788.31±540.77	6383.11±1811.66	-0.121	0.80146	-0.517	0.08868	-1.134	0.00000	0.00000	pubmed
ENSMUSG00000024778	Fas	171.42±28.98	118.76±10.19	151.04±6.66	82.85±10.98	-0.529	0.00553	-0.181	0.49846	-1.051	0.00000	0.00000	pubmed
ENSMUSG00000025092	Hspa12a	565.08±41.65	549.78±39.9	391.72±59.88	239.59±83.7	-0.040	0.96033	-0.529	0.13121	-1.239	0.00000	0.00000	pubmed
ENSMUSG00000025161	Slc16a3	132.77±39.15	130.59±12.69	98.11±9.46	45.17±18.11	-0.023	0.98681	-0.436	0.42254	-1.559	0.00001	0.00001	pubmed
ENSMUSG00000025314	Ptprj	1149.15±24.63	924.23±74.94	1014.63±60.84	551.13±94.17	-0.314	0.03574	-0.180	0.33544	-1.061	0.00000	0.00000	pubmed
ENSMUSG00000025429	Pstpip2	299.55±31.54	298.68±23.32	285.56±40.98	180.6±51.21	-0.004	0.99729	-0.069	0.90227	-0.732	0.00706	0.00706	pubmed
ENSMUSG00000025498	Irf7	985.41±51.31	1170.85±89.14	790.64±43.75	535.47±96.44	0.249	0.26168	-0.318	0.12758	-0.881	0.00000	0.00000	pubmed
ENSMUSG00000025648	Pfkfb4	754.52±31.56	609.21±33.76	783±64.74	372.96±112.71	-0.309	0.40580	0.053	0.92535	-1.018	0.00002	0.00002	pubmed
ENSMUSG00000026009	Icos	47.53±28.79	25.88±6.36	25.69±17.29	7.34±2.32	-0.877	0.29287	-0.884	1.00000	-2.700	0.00001	0.00001	pubmed
ENSMUSG00000026672	Optn	1084.35±75.48	812.37±37.45	1149.48±56.29	546.09±74.8	-0.417	0.00058	0.084	0.67779	-0.991	0.00000	0.00000	pubmed
ENSMUSG00000026821	Ralgds	725.47±31.74	672.89±43.06	668.34±34.42	432.37±41.4	-0.108	0.61530	-0.118	0.56939	-0.747	0.00000	0.00000	pubmed
ENSMUSG00000026980	Ly75	445.5±43.26	449.32±39.96	366.47±40.77	237.92±34.85	0.013	0.98512	-0.282	0.24504	-0.906	0.00000	0.00000	pubmed
ENSMUSG00000027580	Helz2	1011.42±136	620.13±50.17	747.27±120.45	401.78±57.14	-0.706	0.00018	-0.436	0.05450	-1.332	0.00000	0.00000	pubmed
ENSMUSG00000028251	Tstd3	269.35±20.56	246.83±23.66	280.84±34.4	154.93±28.62	-0.126	0.71094	0.060	0.87745	-0.800	0.00001	0.00001	pubmed
ENSMUSG00000028278	Rragd	238.04±13.22	189.73±34.45	193.19±46.14	121.46±17.55	-0.327	0.38421	-0.302	0.43672	-0.970	0.00012	0.00012	pubmed
ENSMUSG00000029314	Agpat9	932.65±55	767.53±26.82	784.87±126.11	465.61±163.19	-0.281	0.53943	-0.249	0.59563	-1.003	0.00034	0.00034	pubmed
ENSMUSG00000029561	Oasl2	597.1±89.89	651.89±8.63	417.45±51.08	261.83±29.69	0.127	0.69851	-0.516	0.01503	-1.189	0.00000	0.00000	pubmed
ENSMUSG00000030865	Chp2	1467.32±170.58	1200.63±141.35	1251.94±113.38	757.43±178.07	-0.289	0.35134	-0.229	0.49534	-0.955	0.00000	0.00000	pubmed
ENSMUSG00000030909	Anks4b	1402.82±82.62	1222.29±125.17	1311.57±123.42	791.63±179.58	-0.199	0.61436	-0.097	0.83526	-0.826	0.00036	0.00036	pubmed
ENSMUSG00000031378	Abcd1	467.94±34.49	419.7±7.87	322.06±17.14	225.19±29.52	-0.157	0.61629	-0.539	0.01156	-1.056	0.00000	0.00000	pubmed
ENSMUSG00000031557	Plekha2	858.61±97.02	581.09±49.79	620.24±85.92	383.71±47.53	-0.563	0.00417	-0.469	0.03072	-1.162	0.00000	0.00000	pubmed
ENSMUSG00000032300	1700017B05Rik	273.42±24.68	238.64±6.72	213.35±23.72	149.91±11.86	-0.197	0.49910	-0.358	0.13749	-0.868	0.00000	0.00000	pubmed
ENSMUSG00000033538	Casp4	548.66±27.22	435.22±16.53	433.34±62.42	216.8±45	-0.334	0.20622	-0.340	0.20345	-1.340	0.00000	0.00000	pubmed
ENSMUSG00000034738	Nostrin	852.51±68.09	713.99±21.33	822.48±76.13	441.89±122.61	-0.256	0.36545	-0.052	0.90149	-0.949	0.00000	0.00000	pubmed
ENSMUSG00000034919	Ttc22	867.75±66.99	576.7±58.13	776.01±17.59	376.25±68.15	-0.589	0.00262	-0.161	0.59716	-1.207	0.00000	0.00000	pubmed
ENSMUSG00000035429	Ptprh	1794.84±154.95	1501.5±81.08	1577.56±53.76	903.79±243.43	-0.257	0.45744	-0.186	0.62337	-0.990	0.00000	0.00000	pubmed
ENSMUSG00000035673	Sbno2	851.83±53.42	545.03±8.84	552.62±30.16	375.24±29.75	-0.644	0.00000	-0.624	0.00001	-1.183	0.00000	0.00000	pubmed
ENSMUSG00000035692	Isg15	131.13±26.83	93.64±5.12	79.09±11.55	38.68±2.84	-0.484	0.06417	-0.729	0.00238	-1.762	0.00000	0.00000	pubmed
ENSMUSG00000036769	Wdr44	187.62±24.95	140.22±26	150.72±10.31	95.68±6.77	-0.420	0.09797	-0.315	0.26893	-0.972	0.00000	0.00000	pubmed
ENSMUSG00000038871	Bpgm	826.88±82.78	607.1±56.62	605.16±21.28	411.49±54.27	-0.446	0.00807	-0.450	0.01023	-1.008	0.00000	0.00000	pubmed
ENSMUSG00000039501	Znfx1	657.25±49.94	486.48±19.2	545.99±14.54	298.25±18.01	-0.434	0.00042	-0.267	0.06992	-1.139	0.00000	0.00000	pubmed
ENSMUSG00000039616	Mocos	314.14±13.51	247.97±11.53	245.5±21.18	145.65±24.35	-0.341	0.05151	-0.356	0.04477	-1.112	0.00000	0.00000	pubmed
ENSMUSG00000040033	Stat2	545.79±59.69	447.64±28	407.13±16.53	285.47±23.27	-0.286	0.09757	-0.423	0.00699	-0.935	0.00000	0.00000	pubmed
ENSMUSG00000040339	Fam102b	930±81.27	820.32±38.6	768.24±63.64	533.62±70.01	-0.181	0.44402	-0.276	0.18495	-0.802	0.00000	0.00000	pubmed
ENSMUSG00000041372	B4galnt3	204.65±15.59	148.25±16.99	196.72±18.95	75.33±37.36	-0.465	0.42464	-0.057	0.95277	-1.445	0.00016	0.00016	pubmed
ENSMUSG00000041779	Tram2	255.54±3.44	226.82±17.96	343.84±18.76	157.25±24.49	-0.172	0.56008	0.428	0.04481	-0.699	0.00009	0.00009	pubmed
ENSMUSG00000041827	Oasl1	412.74±90.2	269.89±9.41	334.29±94.58	119.48±19.37	-0.613	0.06179	-0.305	0.47008	-1.790	0.00000	0.00000	pubmed
ENSMUSG00000043079	Synpo	849.63±28.99	586.17±25.27	720.23±91.47	319.93±53.75	-0.536	0.03612	-0.239	0.48562	-1.410	0.00000	0.00000	pubmed
ENSMUSG00000048279	Sacs	60.72±17.39	56.32±17.31	62.92±14.25	27.65±4.22	-0.109	0.89992	0.053	0.95589	-1.132	0.00503	0.00503	pubmed
ENSMUSG00000049502	Dtx3l	1327.08±151.23	801.79±77.44	1053.02±61.78	434.22±31.51	-0.727	0.00000	-0.334	0.03505	-1.611	0.00000	0.00000	pubmed
ENSMUSG00000050471	Fam118b	277.74±24.54	195.37±11.89	195.85±3.74	138.26±27.14	-0.507	0.01011	-0.504	0.01378	-1.009	0.00000	0.00000	pubmed
ENSMUSG00000050854	Tmem125	158.99±3.79	130.84±8.63	157.78±1.09	92.37±5.48	-0.281	0.28778	-0.011	0.98784	-0.784	0.00002	0.00002	pubmed
ENSMUSG00000054435	Gimap4	399.32±193.25	239.14±74.24	246.99±124.04	114.27±8.38	-0.740	0.17855	-0.693	0.23039	-1.805	0.00001	0.00001	pubmed
ENSMUSG00000056144	Trim34a	100.97±11.55	80.22±12.02	98.61±19.39	49.17±7.93	-0.332	0.37637	-0.033	0.95987	-1.041	0.00005	0.00005	pubmed
ENSMUSG00000058063	Trim31	1387.41±12.91	950.47±9.76	1161.83±70.14	591.29±76.38	-0.546	0.00003	-0.256	0.13189	-1.231	0.00000	0.00000	pubmed
ENSMUSG00000060477	Irak2	460.87±26.53	319.42±13.67	401.89±12.61	189.87±37.26	-0.529	0.03384	-0.197	0.57134	-1.281	0.00000	0.00000	pubmed
ENSMUSG00000063286	Gm8995	220.31±65.9	158.16±63.73	150.82±70.25	77.7±5.53	-0.478	0.43078	-0.546	0.35301	-1.503	0.00016	0.00016	pubmed
ENSMUSG00000066258	Trim12a	234.41±27.57	213.19±11.82	216.28±32.78	133.47±14.8	-0.137	0.67856	-0.115	0.73288	-0.814	0.00001	0.00001	pubmed
ENSMUSG00000068246	Apol9b	54.2±24.64	54±2.2	57.3±13.2	24.23±4.78	-0.004	0.99729	0.080	0.89782	-1.166	0.00018	0.00018	pubmed
ENSMUSG00000068735	Trp53i11	643.68±44.07	535.54±47.28	473.41±73.01	331.47±57.51	-0.265	0.36655	-0.443	0.07894	-0.957	0.00000	0.00000	pubmed
ENSMUSG00000070327	Rnf213	1345.47±202.24	1118.51±178.87	882.61±57.82	553.45±22.33	-0.266	0.38287	-0.608	0.01023	-1.282	0.00000	0.00000	pubmed
ENSMUSG00000073399	Trim40	548.13±16.25	484.64±40.58	336.8±18.34	237.17±56.04	-0.178	0.62904	-0.703	0.00348	-1.210	0.00000	0.00000	pubmed

Ensgene	Symbol	HP	HD	ZP	ZD	LFC HD-HP	FDR HD-HP	LFC ZP-HP	FDR ZP-HP	LFC ZD-HP	FDR ZD-HP	Ratio	Links
ENSMUSG00000074261	Erich4	442.41±22.7	329.01±17.57	386.4±38.86	215.65±54.69	-0.427	0.15272	-0.195	0.61182	-1.038	0.00000	0.00000	pubmed
ENSMUSG00000078942	Naip6	2031.65±171.44	1487.84±70.67	1529.45±200.59	934.62±100.45	-0.449	0.01570	-0.410	0.04092	-1.121	0.00000	0.00000	pubmed
ENSMUSG00000079563	Pglyrp2	28.96±10.4	18.11±4.97	18.04±6.93	8.98±0.87	-0.672	0.05726	-0.672	0.05507	-1.678	0.00000	0.00000	pubmed
ENSMUSG00000079659	Tmem243	378.57±40.86	352.08±7.13	348.85±10.52	246.34±39.66	-0.104	0.67870	-0.118	0.62148	-0.622	0.00001	0.00001	pubmed
ENSMUSG00000085683	9130409J20Rik	1341.65±134.89	786±42.84	1054.95±115.67	494.82±113.43	-0.771	0.00083	-0.347	0.26658	-1.440	0.00000	0.00000	pubmed
